# Characteristic gene alterations in primary gastrointestinal T- and NK-cell lymphomas

**DOI:** 10.1038/s41375-018-0309-4

**Published:** 2019-01-23

**Authors:** Gunho Lee, Hyang Joo Ryu, Ji Woon Choi, Hyundeok Kang, Woo Ick Yang, In Seok Yang, Mi-kyoung Seo, Sangwoo Kim, Sun Och Yoon

**Affiliations:** 1 Department of Biomedical Systems Informatics, Yonsei University College of Medicine, Seoul, Republic of Korea; 2 Graduate Program for Nanomedical Science, Yonsei University, Seoul, Republic of Korea; 3 Department of Pathology, Yonsei University College of Medicine, Severance Hospital, Seoul, Republic of Korea; 4 Department of Pathology, Yonsei University Wonju College of Medicine, Wonju, Republic of Korea; 5 Brain Korea 21 PLUS Project for Medical Science, Yonsei University College of Medicine, Seoul, Republic of Korea

**Keywords:** Lymphoma, Haematological cancer

## To the Editor:

Systemic T and natural killer (NK) cell lymphomas (systemic TNKLs) are malignancies stemming from lymphocytes of T- and NK-cell lineage that preferentially occur in East Asians. Despite an aggressive nature and poor patient outcomes, their rarity and histological heterogeneity have limited the development of effective therapeutic options. Several subtypes have been described for TNKLs according to their cellular origin and site of occurrence: these include angioimmunoblastic T-cell lymphoma (lymph nodes) and extranodal NK/T-cell lymphoma (ENKTL) (nasal/paranasal sites of the head/neck). Some TNKLs, such as enteropathy-associated T-cell lymphoma or monomorphic epitheliotropic intestinal T-cell lymphoma (MEITL), are frequently found along the gastrointestinal (GI) tract and are known to be more aggressive, with patients experiencing bleak clinical outcomes [1]. While this might indicate a site-specific preference in the formation and progression of TNKLs, the molecular biology underlying such preference has not been fully elucidated. In this study, we sought to investigate genetic alterations in primary GI-TNKLs in a comparative manner to characterize the molecular features thereof and to gain insights into the site-specific tumorigenesis of TNKLs.

From Severance Hospital Cancer Registry data, 18 primary GI-TNKLs were collected: GI-TNKL was defined according to the definitions proposed by Lewin et al. and the fourth revision of World Health Organization classification [1, 2]. The 18 cases consisted of six MEITLs, six ENKTLs, three anaplastic large cell lymphomas, and three intestinal T-cell lymphomas not otherwise specified (ITCL-NOS). In addition, 28 cases of non-GI- TNKL were collected for comparative analysis. The complete list of samples, clinical/pathological features, and overall workflow for sample collection are described in the accompanying Supplementary Data (Supplementary Tables 1 and 2, Supplementary Figures 1–3, and Supplementary Appendix).

Initially, we conducted whole-exome sequencing (WES) analysis for six GI-TNKL samples (three MEITLs and three ENKTLs) to obtain a rough profile of somatic mutations. After assessment of the data quality (Supplementary Table 3), we applied the Genome Analysis Toolkit best practice pipeline on the WES data to discover somatic variants (Supplementary Figure 4 and Supplementary Appendix) and identified 230 genes with somatic mutations. We also conducted a literature survey to obtain additional gene variants: mutations in 187 lymphoma-related genes were reported in previous genomic studies (Supplementary Figure 5A). Finally, we constructed a targeted panel of 417 genes for deeper analysis of GI- and non-GI-TNKLs (Supplementary Table 4).

Using the targeted panel, we conducted deep targeted sequencing (~900×) for 46 TNKL samples (18 GI- and 28 non-GI-TNKLs) (Supplementary Figure 5B and Supplementary Appendix). At this stage, we applied more stringent filtering for genes from large public germline databases and frequent false positive genes (referred to as “blacklist” genes) (Supplementary Figure 4, Supplementary Table 5, and Supplementary Appendix). This variant analysis identified 880 nonsynonymous somatic mutations at 833 unique sites (19.1 total and 18.1 unique mutations per patient) (Supplementary Tables 6 and 7). We assumed that a high mutation load (8.02/Mb) resulted from the targeted sequencing. Mutation spectrums, base-substitution frequency (C > T enriched), and Ti/Tv ratio (~2.6) were similar between GI- and non-GI-TNKLs and reflected the typical characteristics of cancers (Supplementary Figures 6 and 7).

**Fig. 1:**
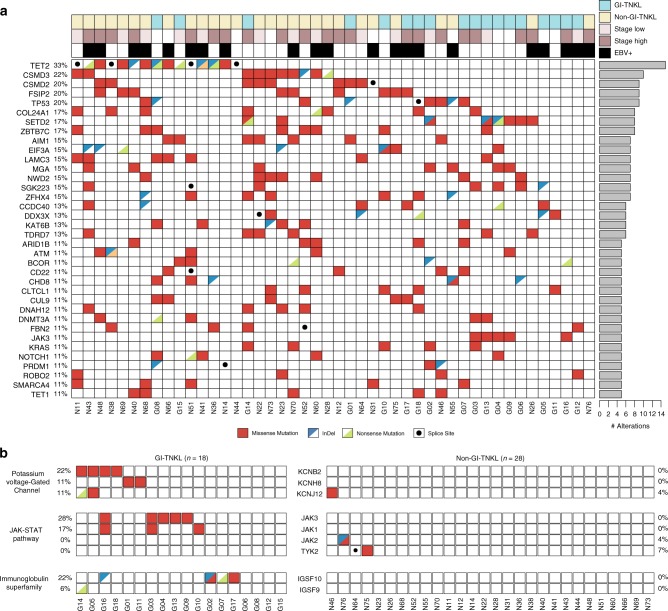
Genomic landscape of alterations in GI-TNKL and non-GI-TNKL. **a** Distribution of well-known somatic alterations in 46 GI-TNKL and non-GI-TNKL patients. Sites of origin (GI-TNKL = sky blue, non-GI-TNKL = yellow), stages (high = brown, low = pink), and EBV positivity (black) are displayed along the top. Individual patients and cancer type (GI-TNKL [G] or non-GI-TNKL [N]) are indicated at the bottom. Frequencies of the mutations in the study population are displayed on the left side of the table. Bars on the right show the total numbers of alterations in each gene. Mutation types are indicated in different colors: missense (red), indel (blue triangle), nonsense (green triangle), or splice site (black dot). **b** Alteration landscape showing recurrent distributions of mutations in key pathways discovered in GI-TNKL patients

Inspecting the genetic landscape of nonsynonymous mutations (Fig. 1a), we discovered that *TET2* was the most frequently mutated gene in all TNKL samples (15/46, 33%), followed by *CSMD3* (10/46, 22%), *CSMD2* (10/46, 20%), *FSIP2* (10/46, 20%), and *TP53* (9/46, 20%). Frequent indels, nonsense mutations, and splice site variants in *TET2* (10/15, 66%) and *TP53* (4/9, 44%) implied that mutations in these tumor suppressor genes may act in a loss-of-function manner. *SETD2* mutations were also observed in 17% of samples, corresponding to previous reports [3].

We further analyzed the site-specificity of the detected mutations (Fig. 1b). While previously known mutant genes were highly present in non-GI-TNKLs (46, 32, and 25% for *TET2*, *CSMD3*, and *CSMD2*, respectively), we were able to find a few novel mutations enriched in GI-TNKLs that are important in three different biological functions: the voltage-gated potassium (Kv^+^) channel, the JAK-STAT pathway, and the immunoglobulin superfamily (see below).

**Fig. 2:**
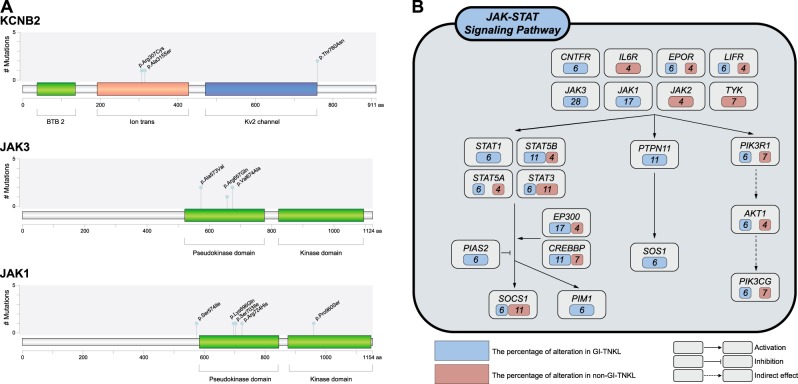
*KCNB2*, *JAK3*, and *JAK1* lollipop plot and the JAK-STAT signaling pathway. **a** *KCNB2*, *JAK3*, and *JAK1* lollipop plots. The diagrams represent the protein domains of *KCNB2, JAK3*, and *JAK1* genes. Mutations are plotted along the *x*-axis, and the numbers of the mutations presented along the *y*-axis. Blue dots indicate individual missense mutations. **b** The schematic diagram represents genomic alterations found in the JAK-STAT signaling pathway. The blue boxes (GI-TNKL cases) and red boxes (non-GI-TNKL) indicate the variant frequencies of mutations found in the pathway. Each arrow represents the type of interaction

With regard to Kv^+^ channel pathway genes, eight mutations (seven missense and one nonsense single nucleotide variants) in *KCNB2* (4/18, 22%), *KCNH8* (2/18, 11%), and *KCNJ12* (2/18, 11%) were discovered in six GI-TNKL samples. These genes encode Kv^+^ channel proteins that mediate transmembrane potassium transport in excitable membranes, which is primarily activated in brain and smooth muscle cells of the GI-tract [4]. Further analysis demonstrated that the *KCNB2* mutations were located in the ion transport and Kv2 channel domains (Fig. 2a). In silico protein modeling and multiple sequence alignment predicted that one of the *KCNB2* mutations (Arg307Cys) results in a critical defect in the function of voltage-gated channels (Supplementary Figures 8, 9 and Supplementary Appendix), while the others remained inconclusive. Compared to normal tonsil tissue, non-GI-TNKL tissue, and GI-TNKL tissue with wild-type KCNB2, GI-TNKL tissue with KCNB2 mutation exhibited lower levels of *KCNB2* mRNA upon reverse transcription PCR, although statistical significance was not observed (Supplementary Figure 10 and Supplementary Appendix). Immunohistochemical protein expression was correlated with mRNA expression of *KCNB2*. Also, low expression of KCNB2 protein was found to be related with an inferior overall survival rate in systemic mature T- and NK-cell lymphomas regardless of GI or non-GI site (Supplementary Table 8, Supplementary Figure 11 and Supplementary Appendix).

Associations between altered regulation of the Kv^+^ channel and cancer development have been continuously reported, such as mutations of *KCNH1* in breast cancer and acute myeloid leukemia [5, 6], of *KCNH2* in glioblastoma [7], and of *KCNA3* in pancreatic cancer [8]. In T cells, aberrations of Kv^+^ channels have been shown to induce changes in intracellular K^+^ and Ca^+^ concentrations and to impair T-cell receptor-driven Akt-mTOR signaling and Ca2^+^ signaling pathways, thereby triggering T-cell activation [9]. In addition, such aberrations may induce a blockade of T-cell effector function by eliciting an ionic checkpoint, which results in immune suppression within the tumor microenvironment [9]. Thus, we conjectured that the newly found mutations in the Kv^+^ channel family genes might be a potential driving mechanism of T-cell lymphoma and cancer immunity. Moreover, we suspect that the site-specificity of the mutations may mirror differences in the tumor microenvironment, considering the known functional roles of Kv^+^ channels in the GI tract, including electrolyte and substrate transport, cell migration, cell proliferation, and apoptosis [10].

The JAK-STAT pathway is a well-known mutation target in systematic TNKLs [11–14]. In the present study, mutations in the JAK-STAT pathway showed GI specificity by presenting only in *JAK3* (5/18, 28%) and *JAK1* (3/18, 17%). Most previous studies have reported *JAK2* mutations only. The newly found *JAK3* and *JAK1* mutations were located in the pseudokinase and kinase domains (Fig. 2a). Further, we found more GI-specific mutations in the downstream genes of JAK-STAT pathways, including *STAT1*, *PTPN11*, and *SOS1*, which might indicate GI-TNKL-specific aberrations in the pathway (Fig. 2b). Although further study should follow, these findings suggest that differences in the tumor microenvironment might be related with the vulnerability of GI-TNKLs to genetic mutations in *JAK3* and *JAK1*.

 Mutations in *IGSF9* (1/18, 6%) and *IGSF10* (4/18, 22%), members of the immunoglobulin superfamily, were found exclusively in GI-TNKLs. So far, no strong associations have been reported between immunoglobulin function and TNKLs, except one recent study that showed a recurrent *IGSF10* mutation in familial gastric and colorectal cancer [15]. We expect that further in-depth studies can test the vulnerability of resident cells, such as mucosa epithelial cells and immune T cells, in the milieu of the GI-tract to *IGSF10* mutations.

Finally, we conducted direct Sanger sequencing to confirm the presence of mutations in *KCNB2*, *JAK3*, and *JAK1* (Supplementary Table 9 and Supplementary Appendix). In seven cases in which genomic DNA were available, nine mutations were validated (Supplementary Figure 12 and Supplementary Appendix).

In conclusion, we noted characteristic mutations of *KCNB2*, as well as *JAK3*, *JAK1*, and *IGSF10*, in GI-TNKLs. Although more comprehensive work is needed, the present findings provide some insights into understanding the physiological and pathological links between these genes and GI-TNKL genesis and into the potential for targeted therapy against tumor cells and the tumor microenvironment associated therewith.

## Supplementary information

Supplementary appendix

Supplementary figure and table legends

Supplementary figure 1

Supplementary figure 2

Supplementary figure 3

Supplementary figure 4

Supplementary figure 5

Supplementary figure 6

Supplementary figure 7

Supplementary figure 8

Supplementary figure 9

Supplementary figure 10

Supplementary figure 11

Supplementary figure 12

Supplementary table 1

Supplementary table 2

Supplementary table 3

Supplementary table 4

Supplementary table 5

Supplementary table 6

Supplementary table 7

Supplementary table 8

Supplementary table 9
