# Supplementary material for: Characteristic gene alterations in primary gastrointestinal T- and NK-cell lymphomas
Source: Leukemia. 2019 Jan 23;33(7):1797–832. doi: 10.1038/s41375-018-0309-4 (PMC6755973; doi:10.1038/s41375-018-0309-4)
Supplement: Supplementary file 1 — Supplementary appendix [file 41375_2018_309_MOESM1_ESM.docx]

**Supplementary Appendix**

**Characteristic Gene Alterations in Primary Gastrointestinal T and NK Cell Lymphomas**

Gunho Lee^1,2^*, Hyang Joo Ryu^3^*, Ji Woon Choi^4^, Hyundeok Kang^1,5^, Woo Ick Yang^3^, In Seok Yang^1^, Mi-kyoung Seo^1,5^, Sangwoo Kim^1,5**^, Sun Och Yoon^3**^

^1^Department of Biomedical Systems Informatics, Yonsei University College of Medicine, Seoul, Korea

^2^Graduate Programs for Nanomedical Science, Yonsei University, Seoul, Korea

^3^Department of Pathology, Yonsei University College of Medicine, Severance Hospital, Seoul, Korea

^4^Department of Pathology, Yonsei University Wonju College of Medicine, Wonju, Korea

^5^Brain Korea 21 PLUS Project for Medical Sciences, Yonsei University College of Medicine, Seoul, Korea

*These authors contributed equally to the work as first authors.

**These authors contributed equally to the work as corresponding authors.

**Contents**

Materials and Methods

Results

**Materials and Methods**

**Selection of primary gastrointestinal T and NK cell lymphomas**

From the Severance Hospital Cancer Registry Data, 30 cases of systemic TNKL affecting the GI tract were retrieved among 451 cases of overall TNKL diagnosed between January 2001 and February 2016. Among them, 27 cases satisfied the criteria for primary GI lymphoma as defined by Lewin et al. (1): these patients presented with GI symptoms and/or a tumor predominantly located in the GI tract (1). For all selected cases, H&E staining; immunohistochemistry for CD3, CD4, CD8, CD56, CD30, CD103, granzyme B, TIA-1, and ALK; and in situ hybridization for EBV-encoded RNA were performed. Histopathologic features were reviewed by experienced hematopathologists (S.O.Y, J.W.C, and W.I.Y), and histologic classification was undertaken according to the 2008 World Health Organization classification criteria and the revised 4^th^ WHO classification(2-4).

As well known in Asian data about enteropathy-associated T cell lymphoma (EATL) (5),our cohort of EATL also included only type II enteropathy-associated T-cell lymphoma according to 2008 WHO classification. Type II EATLs are now classified as monomorphic epitheliotropic intestinal T-cell lymphoma (MEITL) in accordance with the recently revised WHO classification ; therefore, we chose to use the term MEITL in the present study for the sake of simplicity and uniformity. In addition, cases showing the histology of peripheral T cell lymphoma, not otherwise specified (PTCL-NOS), which primarily involve the gastrointestinal tract but could not be categorized as MEITL or other T/NK cell lymphoma subtypes, were defined as intestinal T cell lymphoma, not otherwise specified (ITCL-NOS) according to the recently revised WHO classification(2-4).

Cases of non-GI-tract TNKL were included as a control and validation cohort. All clinical information, including survival data, was collected from the Severance Hospital Cancer Registry Data and medical records. The study was approved by the Institutional Review Board of Severance Hospital (Protocol No. 4-2016-0397). Overall workflow and detailed information are summarized in Supplementary Figure 1 and Supplementary Tables 1 and 2.

**Tissue microarray preparation, immunohistochemistry, in situ hybridization, and T cell receptor gene clonality assay**

One to three different representative tumor areas per sample were selected for tissue microarray (TMA) construction. Core tissues 3 mm in diameter were taken from donor tissue blocks and arranged in recipient TMA blocks using a trephine apparatus.

Immunohistochemistry was performed on 4-μm tissue sections with a Ventana Bench Mark XT Autostainer (Ventana Medical Systems, Tucson, AZ, USA) as described previously(6). Primary antibodies included those for CD3 (dilution 1:200; LabVision, Fremont, CA, USA), CD4 (RTU; clone CD4-1F6; Novocastra, Leica, Newcastle Upon Tyne, UK), CD8 (RTU; clone C8/144B; Dako, Glostrup, Denmark), CD56 (dilution 1:100; clone CD564; Novocastra, Leica,), CD30 (dilution 1:50; clone Ber-H2, Dako), CD103 (dilution 1:50; polyclonal, Novus Biologics, Littleton, USA), granzyme B (dilution 1:50; clone GrB-7, Dako), TIA-1 (dilution 1:100; clone 2G9A10F5, Beckman Coulter, Marseille, France), ALK (dilution 1:50; clone ALK1, Dako), Ki-67 (dilution 1:150; clone MIB-1, Dako), and KCNB2 (dilution 1:300; polyclonal; Aviva Systems Biology, CA, USA). In situ hybridisation for EBV-encoded RNAs was performed with digoxigenin-labelled probes (Novocastra, Newcastle, UK) and a Ventana Bench Mark XT Autostainer (Ventana XT, AZ). Positivity for CD3, CD4, CD8, CD56, CD30, CD103, granzyme B, TIA-1, ALK, or EBV-encoded RNA was defined as expression in ≥30% of tumor cells, respectively(7).

KCNB2 expression was semiquantitatively calculated by the H-score method with a total score ranging from 0-300(8). KCNB2 expression was observed in the cytoplasmic membrane, cytoplasm, and nucleus of tumor and normal endothelial cells. The predominant intensity score between cytoplasm and nuclear staining (0, no staining; 1, weak or barely detectable staining; 2, distinct brown staining; 3, strong dark brown staining) was multiplied by the percentage of positive cells (0-100%). Cut-off values for high and low expression were determined in accordance with the mean value.

For T cell receptor gene clonality assay, DNA was extracted first from formalin-fixed paraffin-embedded (FFPE) tumor tissue blocks. T cell receptor-gamma, -beta, and -delta genes were then assayed with IdentiClone *TCRG, TCRB,* and *TCRD* Gene Clonality Assay Kits (Invivoscribe, CA, USA), which are manufactured based on the BIOMED-2 study for PCR-based clonality testing(9). The PCR products were finally analyzed by gel capillary electrophoresis.

**Whole exome sequencing analysis**

After reviewing demographic features of the primary GI-TNKLs and assessing tissue availability for further molecular analysis, three cases of MEITL and three cases of extranodal NK/T-cell lymphoma (ENKTL), which were the most common subtypes among the primary GI-TNKLs, were selected for sequencing analysis (Supplementary Table 1). Clonality assays for T cell receptor-gamma, -beta, and -delta genes were performed to confirm the recorded histopathologic diagnoses (summarized in Supplementary Figure 3). As these six cases involved radical surgery, matching normal bowel wall and tumor tissue was available. To avoid contamination, normal bowel wall specimens were selected at a distance at least 3 cm from the tumor margin, and were confirmed microscopically to be tumor cell-free. Genomic DNA was extracted from the FFPE tissue blocks of the matching normal bowel wall and tumor tissue, and exome regions were captured using the Agilent SureSelect Human All Exon V5 probe set (Agilent, CA, USA). Whole exome sequencing was performed with an Illumina HiSeq 2500 sequencer (Illumina, CA, USA). All of these experiments were performed at Macrogen (Seoul, Korea).

For analysis of the exome sequencing data (Supplementary Table 3), we followed the GATK (Genome Analysis Toolkit) Best Practice for somatic short variant discovery v3.5 (Supplementary Figure 4) (10). Quality filtering and adaptor stripping were applied using FastQC v0.11.2 (11). The raw sequence reads in FASTQ files were then mapped to the UCSC hg19 human reference using BWA-mem v0.7.10 (12). PCR artificial duplicates were excluded using Picard (<http://broadinstitute.github.io/picard>). Indel realignment and base quality score recalibration were performed using GATK. Mutect2 from GATK version 3.5 was used to call somatic single nucleotide variants and indels. Variants were further filtered using the following cut-off values: (i) read-depth < 30, (ii) mutant allele count < 5, and (iii) allele frequency in normal population (from dbSNP) > 0.1 (13). The functional impacts of the variants were annotated using SnpEff v4.3 and SnpSift. Only variants that were predicted to be of ‘moderate’ or ‘high’ functional impact were used for further analysis (14).

**Targeted deep sequencing analysis**

For targeted deep sequencing analysis (Supplementary Table 3), a customized panel of 417 lymphoma-associated genes was designed and utilized for targeted sequencing (Supplementary Table 4 for the entire list of genes, Supplementary Figure 5A) of 46 T/NK cell lymphoma samples (18 primary GI-TNKLs and 28 non-GI-TNKLs occurring in lymph nodes and the nasal cavity) (Supplementary Figure 5B). gDNA from the samples was extracted using the GENEREAD FFPE KIT (QIAGEN) and captured by Agilent SureSelect XT Custom Kits (Agilent, CA, USA). Paired-end sequencing (2 x 100 bp) was performed using an Illumina HiSeq 2500 sequencer (Illumina, CA, USA) with an expected read depth of 1000.

Variant analysis was conducted by a process similar to that for whole exome sequencing (Supplementary Figure 4). However, a much stricter filtering process was applied to remove germ line SNPs without control samples. First, we excluded variants that were present in >1% of the human population using the Exome Aggregation Consortium (<http://exac.broadinstitute.org/>) (15) and Korean Reference Genome Project (152.99.75.168/KRGDB/menuPages/intro.jsp). Second, we further utilized FLAGS (16), MutSig CV false variants (17), and the false positive gene list from mass genome (http://massgenomics.org/2013/06/ngs-false-positives.html) to eliminate well-known false positive genes (Supplementary Table 5). Third, all remaining variants were manually inspected by sequence analysis experts using the Integrative Genomics Viewer (18).

**Validation Sanger sequencing**

To validate genetic alterations in *KCNB2*, *JAK3*, and *JAK1*, direct Sanger sequencing was performed. The tested samples and primer sequences used are summarized in Supplementary Table 9. Genomic DNA was extracted from FFPE samples using ReliaPrep™ FFPE gDNA Miniprep System (Promega, WI, USA). PCR was then performed with i-Star Taq DNA polymerase (iNtRON Biotechnology, Korea) usingthe following cycling parameters: 94°C for 2 min, 40 cycles of 94°C for 20 sec, 54°C for 10 sec, and 72°C for 20 sec. PCR products were purified with mega quick-spin total fragment DNA Purification kits (iNtRON Biotechnology). Sanger sequencing experiments were performed with an ABI Prism 3730 XL DNA sequencer (Thermo Fisher Scientific) by Xenotech (Daejeon, Korea)

**Gene expression analysis**

RNA was isolated using TRIzol reagent (Thermo Fisher Scientific, MA, USA), and cDNA was constructed using a cDNA synthesis kit (11754- 050, Invitrogen). Synthesized primers (Integrated DNA Technologies, IA, USA) for KCNB2 were as follows: forward, CTGGAAGTGTGCGACGACTA; reverse, GGCAGCAGGACTCCAAGTAG. Quantitative PCR was performed using a QuantStudio 3D Real-Time PCR A28132 system (Applied Biosystems; Thermo Fisher Scientific) with the following cycling parameters: 95°C for 2 min, 40 cycles of 95°C for 5 sec, and 60°C for 30 min. Relative mRNA expression levels were normalized to the GAPDH level by the comparative method (2-ddCt).

**Preparation of a mutant Kv^+^ channel model**

The SWISS-MODEL (19) was used to generate models for wild-type and R307C/S315A mutant structures of the voltage-gated potassium (Kv^+^) channel protein conferred by the expression of KCNB2. Briefly, we searched appropriate models by inputting amino-acid sequences of α subunits of wild-type and mutant proteins; selected a crystal structure of the Kv1.2-2.1 paddle chimera channel (PDB ID: 2R9R) (20) with highest sequence identity as a template structure, and then built the final models for use in structural analysis. Molecular visualization of the model was conducted using PyMOL version 0.99 (21).

**Statistical analysis**

The chi-square test, Fisher exact test, two-sample t-test, or one-way ANOVA test were used to analyze differences between evaluated variables. Overall survival was measured from date of initial diagnosis to date of death or last follow-up. The Kaplan-Meier method was used to analyze survival rates, and differences therein were compared using the log-rank test. The Cox proportional hazards model was implemented for multivariate analysis. Two-sided *P*-values <0.05 were considered statistically significant. Statistical analyses were conducted using IBM SPSS 23 software for Windows (IBM Corp, Armonk, NY, USA).

**Results**

**Survival analyses according to KCNB2 protein expression**

Protein expression of KCNB2 was analyzed in a cohort of systemic TNKLs of GI and non-GI sites (n=131). In univariate Kaplan-Meier and Cox hazards analysis, low expression of KCNB2 tended to be related to inferior overall survival (Supplementary Figure 11 and supplementary Table 8). Moreover, among the systemic TNKLs, those at GI sites showed inferior overall survival than those at non-GI sites. Age at diagnosis, histologic subtypes, and Ann-Arbor stage also revealed prognostic value for systemic TNKLs (Supplementary Table 8). In multivariate Cox analysis, low expression of KCNB2 exhibited a tendency to independently predict poor overall survival for systemic TNKLs, although statistical significance was not observed (Supplementary Table 8).

**Potential influence of KCNB mutations on Kv^+^ channels**

The monomeric structure of the α subunit of Kv^+^ channels (PDB ID: 2R9R) (20) consists of three domains: an intracellular domain, voltage sensor domain, and pore-forming domain (22). In the voltage sensor domain, positively charged residues (R1-R4, K5, and R6; light blue blocks in Supplementary Figure 9) are located in a helix (S4), which has been shown to play an important role in voltage sensing (22). These residues are counterbalanced by negatively charged residues (red blocks in Supplementary Figure 9) in other helices, and are separated by two hydrophobic residues, as shown in multiple sequence alignment (light pink blocks in Supplementary Figure 9).

Based on the known protein model of Kv^+^ channels, we predicted the functional effects of the observed mutations in two positions of KCNB2: the 307th (R307) and 315th (A315) residues. First, we generated wild-type and mutant models of KCNB2 (Supplementary Figure 8). Interactions from N202 through R307 and E237 to R310 were observed in the wild-type model of KCNB2 (Supplementary Figure 8A, C), wherein N202 is an uncharged residue with polarity that makes it possible to interact with other residues via hydrogen bonding. However, in one mutant model, the altered structure of R307C led to disconnection of the normal interactions between R307 and E237 and between R307 and N202 (Supplementary Figure 8B, D). Such mutation in R307 might cause a critical defect in the function of KCNB2. Meanwhile, the A315 residue is located in the C-terminal of helix S4 and is surrounded by hydrophobic residues at L266, I269, L312, and L321 (Supplementary Figure 8E, F), which have been found to be evolutionary conserved (light pink blocks in Supplementary Figure 9). In the mutant model, the A315S mutation elicited a change in the side chain from hydrophobic to polar characteristics at the position. Considering the amino-acid change itself, one would suspected that it might be sufficient to affect protein function. However, the results from multiple sequence alignment revealed that a Ser residue is found at the corresponding position of A315 in other types of Kv^+^ channels (Supplementary Figure 9). Therefore, A315S mutation might have limited influence on the function of Kv^+^ channels.

**Code availability**

Computer codes and scripts for analysis of WES and targeted sequencing are available on request.

**Reference**

1. Lewin KJ, Ranchod M, Dorfman RF. Lymphomas of the gastrointestinal tract. A study of 117 cases presenting with gastrointestinal disease. Cancer. 1978;42(2):693-707.

2. Swerdlow S, Campo E, Harris N, Jaffe E, Pileri S, Stein H, et al. WHO Classification of Tumors of Hematopoietic and Lymphoid Tissues 4th Ed.(2008). 2008.

3. Swerdlow SH, Campo E, Harris NL, Jaffe ES, Pileri S, Stein H, et al. WHO classification of tumours of haematopoietic and lymphoid tissues (Revised 4th edition). Lyon: International Agency for Research on Cancer; 2017.

4. Swerdlow SH, Campo E, Pileri SA, Harris NL, Stein H, Siebert R, et al. The 2016 revision of the World Health Organization classification of lymphoid neoplasms. Blood. 2016;127(20):2375-90.

5. Delabie J, Holte H, Vose JM, Ullrich F, Jaffe ES, Savage KJ, et al. Enteropathy-associated T-cell lymphoma: clinical and histological findings from the international peripheral T-cell lymphoma project. Blood. 2011:blood-2011-02-335216.

6. Choi CH, Park YH, Lim JH, Choi SJ, Kim L, Park IS, et al. Prognostic implication of semi-quantitative immunohistochemical assessment of CD20 expression in diffuse large B-cell lymphoma. Journal of pathology and translational medicine. 2016;50(2):96.

7. Hans CP, Weisenburger DD, Greiner TC, Gascoyne RD, Delabie J, Ott G, et al. Confirmation of the molecular classification of diffuse large B-cell lymphoma by immunohistochemistry using a tissue microarray. Blood. 2004;103(1):275-82.

8. Park E, Park SY, Kim H, Sun P-L, Jin Y, Cho SK, et al. Membranous insulin-like growth factor-1 receptor (IGF1R) expression is predictive of poor prognosis in patients with epidermal growth factor receptor (EGFR)-mutant lung adenocarcinoma. Journal of pathology and translational medicine. 2015;49(5):382.

9. Van Dongen J, Langerak A, Brüggemann M, Evans P, Hummel M, Lavender F, et al. Design and standardization of PCR primers and protocols for detection of clonal immunoglobulin and T-cell receptor gene recombinations in suspect lymphoproliferations: report of the BIOMED-2 Concerted Action BMH4-CT98-3936. Leukemia. 2003;17(12):2257.

10. McKenna A, Hanna M, Banks E, Sivachenko A, Cibulskis K, Kernytsky A, et al. The Genome Analysis Toolkit: a MapReduce framework for analyzing next-generation DNA sequencing data. Genome research. 2010.

11. Andrews S. FastQC: a quality control tool for high throughput sequence data. 2010.

12. Li H, Durbin R. Fast and accurate long-read alignment with Burrows–Wheeler transform. Bioinformatics. 2010;26(5):589-95.

13. Sherry ST, Ward M-H, Kholodov M, Baker J, Phan L, Smigielski EM, et al. dbSNP: the NCBI database of genetic variation. Nucleic acids research. 2001;29(1):308-11.

14. Cingolani P, Platts A, Wang LL, Coon M, Nguyen T, Wang L, et al. A program for annotating and predicting the effects of single nucleotide polymorphisms, SnpEff: SNPs in the genome of Drosophila melanogaster strain w1118; iso-2; iso-3. Fly. 2012;6(2):80-92.

15. Lek M, Karczewski KJ, Minikel EV, Samocha KE, Banks E, Fennell T, et al. Analysis of protein-coding genetic variation in 60,706 humans. Nature. 2016;536(7616):285.

16. Shyr C, Tarailo-Graovac M, Gottlieb M, Lee JJ, van Karnebeek C, Wasserman WW. FLAGS, frequently mutated genes in public exomes. BMC medical genomics. 2014;7(1):64.

17. Lawrence MS, Stojanov P, Polak P, Kryukov GV, Cibulskis K, Sivachenko A, et al. Mutational heterogeneity in cancer and the search for new cancer-associated genes. Nature. 2013;499(7457):214.

18. Robinson JT, Thorvaldsdóttir H, Winckler W, Guttman M, Lander ES, Getz G, et al. Integrative genomics viewer. Nature biotechnology. 2011;29(1):24.

19. Biasini M, Bienert S, Waterhouse A, Arnold K, Studer G, Schmidt T, et al. SWISS-MODEL: modelling protein tertiary and quaternary structure using evolutionary information. Nucleic acids research. 2014;42(W1):W252-W8.

20. Long SB, Tao X, Campbell EB, MacKinnon R. Atomic structure of a voltage-dependent K+ channel in a lipid membrane-like environment. nature. 2007;450(7168):376.

21. DeLano WL. The PyMOL molecular graphics system. <http://www> pymol org. 2002.

22. Kuang Q, Purhonen P, Hebert H. Structure of potassium channels. Cellular and molecular life sciences. 2015;72(19):3677-93.
