# Supplementary material for: Characteristic gene alterations in primary gastrointestinal T- and NK-cell lymphomas
Source: Leukemia. 2019 Jan 23;33(7):1797–832. doi: 10.1038/s41375-018-0309-4 (PMC6755973; doi:10.1038/s41375-018-0309-4)
Supplement: Supplementary file 2 — Supplementary figure and table legends [file 41375_2018_309_MOESM2_ESM.docx]

**Supplementary Legends**

**Characteristic Gene Alterations in Primary Gastrointestinal T and NK Cell Lymphomas**

Gunho Lee^1,2^*, Hyang Joo Ryu^3^*, Ji Woon Choi^4^, Hyundeok Kang^1,5^, Woo Ick Yang^3^, In Seok Yang^1^, Mi-kyoung Seo^1,5^, Sangwoo Kim^1,5**^, Sun Och Yoon^3**^

^1^Department of Biomedical Systems Informatics, Yonsei University College of Medicine, Seoul, Korea

^2^Graduate Programs for Nanomedical Science, Yonsei University, Seoul, Korea

^3^Department of Pathology, Yonsei University College of Medicine, Severance Hospital, Seoul, Korea

^4^Department of Pathology, Yonsei University Wonju College of Medicine, Wonju, Korea

^5^Brain Korea 21 PLUS Project for Medical Sciences, Yonsei University College of Medicine, Seoul, Korea

*These authors contributed equally to the work as first authors.

**These authors contributed equally to the work as corresponding authors.

**Contents**

Supplementary Figure Legends

Supplementary Tables

Supplemental Reference

**Supplementary Figure Legends**

**Supplementary Fig. 1. Overall workflow of the study**.

After comprehensive epidemiology and histopathology analysis, primary gastrointestinal T and NK cell lymphomas (1⁰ GI TNKL; GI L) were defined. After whole-exome sequencing (WES) of tumor tissue (T) and matched normal intestine tissue (mN) of representative cases of 1⁰ GI-TNKL, target sequencing and/or direct Sanger sequencing was performed for tumor samples (T) of 1⁰ GI-TNKL and other systemic mature T and NK cell lymphomas arising from various nodal and extranodal sites (non-GI-TNKL; non-GI L). These samples were also analyzed by reverse transcription PCR (RT-PCR) to assess gene expression according to the relevant gene mutation. In the RT-PCR step, normal lymphoid tissue of palatine tonsils (N) obtained from cancer-free individuals were added as controls. To assess protein expression of relevant gene mutations, immunohistochemistry (IHC) was performed. In the IHC step, additional tumor tissues were added to assess the prognostic value of the altered protein markers.

Primary cutaneous T and NK cell lymphoma (1⁰ Cutaneous) cases were excluded from the study.

**Supplementary Fig. 2. Representative images of primary gastrointestinal T and NK cell lymphomas.**

The results of H&E staining, immunohistochemistry, and EBV in situ hybridization were captured.

Cases showing infiltration of CD3^+/-^, CD4^-^, CD56^+^ (or CD8^+^), cytotoxic molecules (granzyme B, TAI-1)^+^, and EBV-encoded RNAs^+^ pleomorphic cells of small to medium size with germline T cell receptor gene rearrangement were defined as extranodal NK/T cell lymphoma, nasal type (A). Cases showing infiltration of CD3^+^, CD4^-^, CD8^+^, CD56+, cytotoxic molecules (granzyme B, TAI-1)^+^, and EBV-encoded RNAs^-^ monomorphic cells of small to medium size with clonal T cell receptor gene rearrangement and increased CD8^+^ intraepithelial lymphocytes were defined as monomorphic epitheliotropic intestinal T cell lymphoma (B). Cases showing infiltration of CD30^+^, CD3^-/+^, CD4^+^, CD8^-^, cytotoxic molecules (granzyme B, TAI-1)^+^, and EBV-encoded RNAs^-^ pleomorphic cells of large size with clonal T cell receptor gene rearrangement were defined as anaplastic large cell lymphoma. According to ALK1 expression status, anaplastic large cell lymphoma cases were subclassified into ALK-negative (C) or ALK-positive type. Cases showing histology of peripheral T cell lymphoma, not otherwise specified (PTCL-NOS) that primarily involved the gastrointestinal tract, but could not be categorized as MEITL or other T/NK cell lymphoma subtypes, were defined as intestinal T cell lymphoma, not otherwise specified.

**Supplementary Fig. 3. T cell receptor clonality assay.**

T cell receptor-gamma (TCRG), -beta (TCRB), and –delta (TCRD) gene clonality assay based on BIOMED-2 PCR testing was performed for the six cases that underwent whole-exome sequencing. T cell monoclonality was identified in all monomorphic epitheliotropic intestinal T-cell lymphomas (MEITLs), suggesting T cell origin. None of the three extranodal NK/T cell lymphomas of nasal type (ENKTLs) showed T cell clonality, indicating NK cell origin.

**Supplementary Fig. 4. Workflow of whole-exome sequencing and targeted deep sequencing.**

We used the GATK best pipeline and an additional in-house filter to identify novel variants.

**Supplementary Fig. 5. Flow chart of gene and sample selection in targeted deep sequencing.**

(A) We selected 417 genes for targeted deep sequencing. These genes were noted as somatic mutations in the six GI-TNKLs that underwent whole exome sequencing and in gene sets selected from previous studies. The 417 selected genes are listed in Supplementary Table 4.

(B) Flow chart of sample selection for targeted deep sequencing.

**Supplementary Fig. 6. Somatic mutations identified by whole-exome sequencing and targeted deep sequencing of GI-TNKL and non-GI-TNKL.**

(A) Number of somatic mutations discovered from the GI-TNKL and non-GI-TNKL patients.

(B) In both groups, a C > T substitution was identified most often across the mutation spectrum.

**Supplementary Fig. 7. Summary of somatic mutations in GI-TNKL and non-GI-TNKL, including variant classification, class of SNV, and distribution of transition and transversion.**

**Supplementary Fig. 8. Structural differences between wild type and mutant KCNB2 models in the voltage sensor domain.**

(A) Interactions of R310, E237, R307, and N202 in the wild-type model (see also C). (B) Disconnected interactions of E237, C307, and N202 in the R307C mutant model (see also D). (E) Centered view on A315 in the wild-type model. Note the residue is surrounded by hydrophobic residues at L266, I269, L312, and L321. (F) Centered view on S315 in the A315S mutant model.

**Supplementary Fig. 9. Multiple sequence alignment of human KCNB1, human KCNB2, paddle-chimaera channel, rat Kv2.1, rat Kv1.2, Shaker Kv, KvAP, and KcsA transmembrane regions.**

Secondary structure elements are represented on the top of sequences. Positively charged, negatively charged, and non-polar residues are highlighted by three boxes in light blue, red, and light pink, respectively. R307, R310, N202, and A317 are further displayed to show structural differences between the wild-type and mutant KCNB2 models (Supplementary Fig. 8 for details). Note that we adopted the results from Long SB et al. (1) to align the amino-acid sequences of KCNB1 and KCNB2.

**Supplementary Fig. 10. Representative images of KCNB2 immunohistochemistry (A-D) and KCNB2 expression (E and F).**

(A and B) Cases showing strong diffuse expression and (C and D) cases showing negative or focal, weak expression of KCNB2 protein. (E) When comparing the median value of KCNB2 mRNA expression in each group, relatively lower levels KCNB2 mRNA are seen in the GI TNKL with KCNB2 mutation group, compared to the normal tonsil, non-GI-TNKL, and GI-TNKL with wild-type KCNB2 groups, upon reverse transcription PCR (RT-PCR). (F) Positive correlation between KCNB2 mRNA expression and KCNB2 protein expression was noted. KCNB2 mRNA expression was log2 fold-change normalized to GAPDH (2-ddCt) upon reverse transcription PCR (RT-PCR). KCNB2 protein expression was scored by H-score on a scale of 0 to 300 upon immunohistochemistry.

**Supplementary Fig. 11. Kaplan-Meier analysis of overall survival according to KCNB2 protein expression in systemic mature T and NK cell lymphomas.**

By the log-rank test, low expression of KCNB2 protein was shown to be related to inferior overall survival in patients with systemic mature T and NK cell lymphomas regardless of GI or non-GI sites. KCNB2 protein expression was scored by H-score on a scale of 0 to 300 upon immunohistochemistry.

**Supplementary Fig. 12. *KCNB2*, *JAK3*, and *JAK1* missense mutations confirmed by Sanger sequencing.**

(A) p.Arg307Cys, p.Ala315Ser, and p.Thr760Asn mutations of *KCNB2* in patients G14, G16, G05, and G18.

(B) p.Ala573Val mutation of *JAK3* in patients G09 and G16.

(C) p.Arg724His, p.Ser703Ile, and p.Pro960Ser mutations of *JAK1* in patients G03, G10, and G16.

**Supplementary Tables**

**Supplementary Table 1. Information on each patient included for sequencing analysis**

**Supplementary Table 2. Clinical and pathological features of the patients**

**Supplementary Table 3. Summary of exome and targeted deep sequencing statistics (N=46)**

**Supplementary Table 4. Targeted deep sequencing gene list (417 Genes)**

**Supplementary Table 5. False-positive gene list**

**Supplementary Table 6. GI-TNKL somatic mutations**

**Supplementary Table 7. non-GI-TNKL somatic mutations**

**Supplementary Table 8. Univariate and multivariate Cox analysis for overall survival in patients with systemic mature T and NK cell lymphomas**

**Supplementary Table 9. Primer sets used for Sanger sequencing**

**Supplemental Reference**

1. Long SB, Tao X, Campbell EB, MacKinnon R. Atomic structure of a voltage-dependent K+ channel in a lipid membrane-like environment. nature 2007; 450, 376.
