# Supplementary figures and images for: Characteristic gene alterations in primary gastrointestinal T- and NK-cell lymphomas
Source: Leukemia. 2019 Jan 23;33(7):1797–832. doi: 10.1038/s41375-018-0309-4 (PMC6755973; doi:10.1038/s41375-018-0309-4)

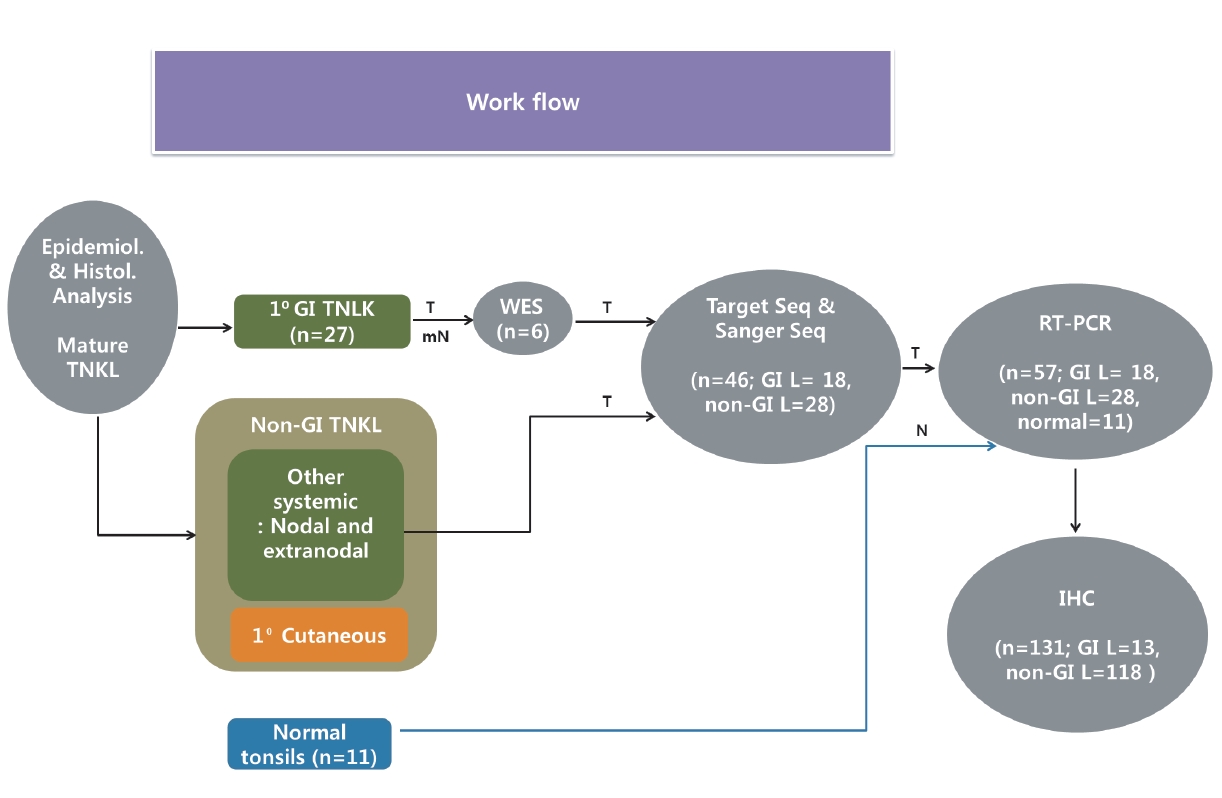

Supplement: Supplementary file 3 — Supplementary figure 1 [file 41375_2018_309_MOESM3_ESM.jpg]

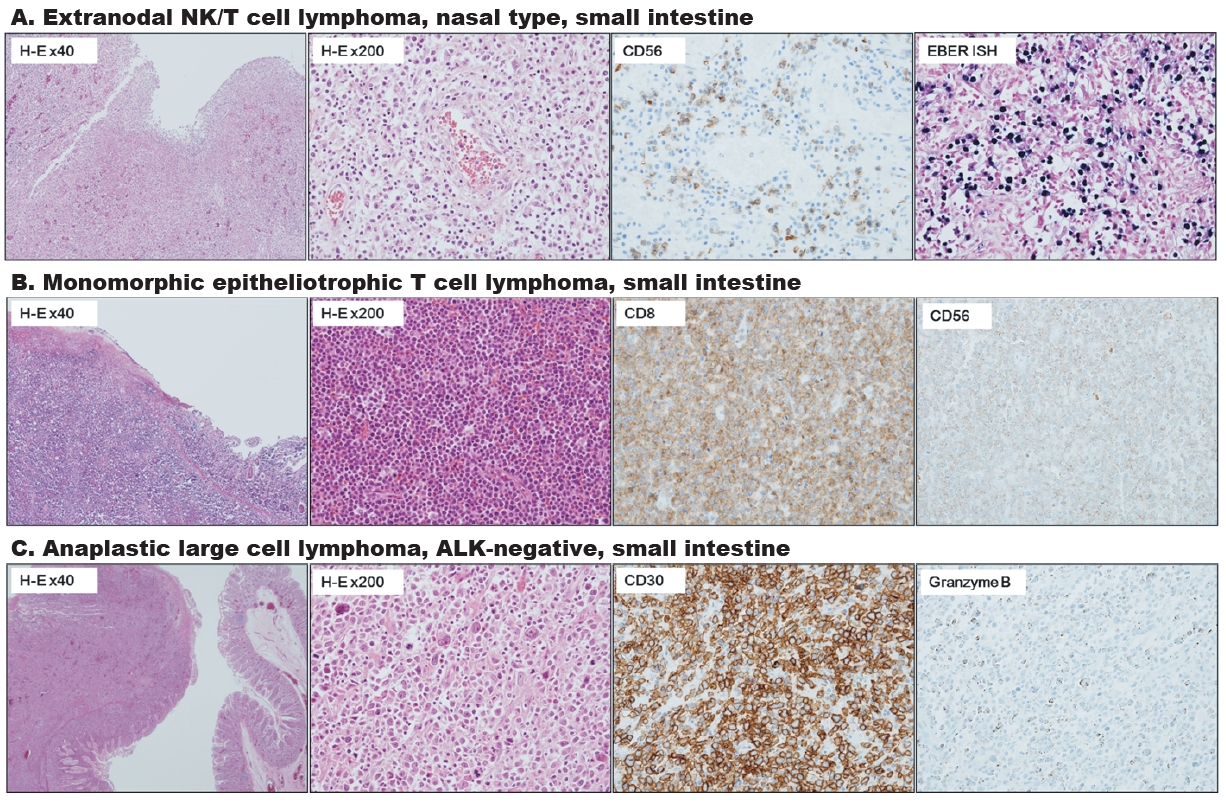

Supplement: Supplementary file 4 — Supplementary figure 2 [file 41375_2018_309_MOESM4_ESM.jpg]

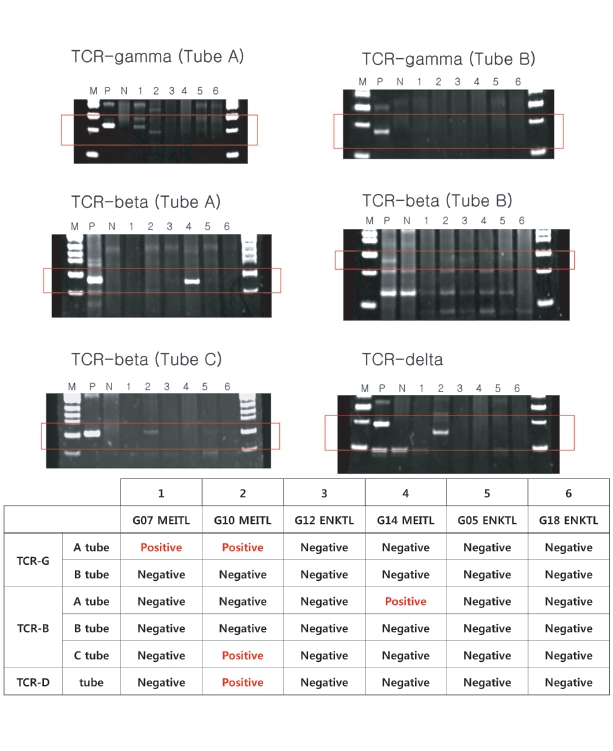

Supplement: Supplementary file 5 — Supplementary figure 3 [file 41375_2018_309_MOESM5_ESM.jpg]

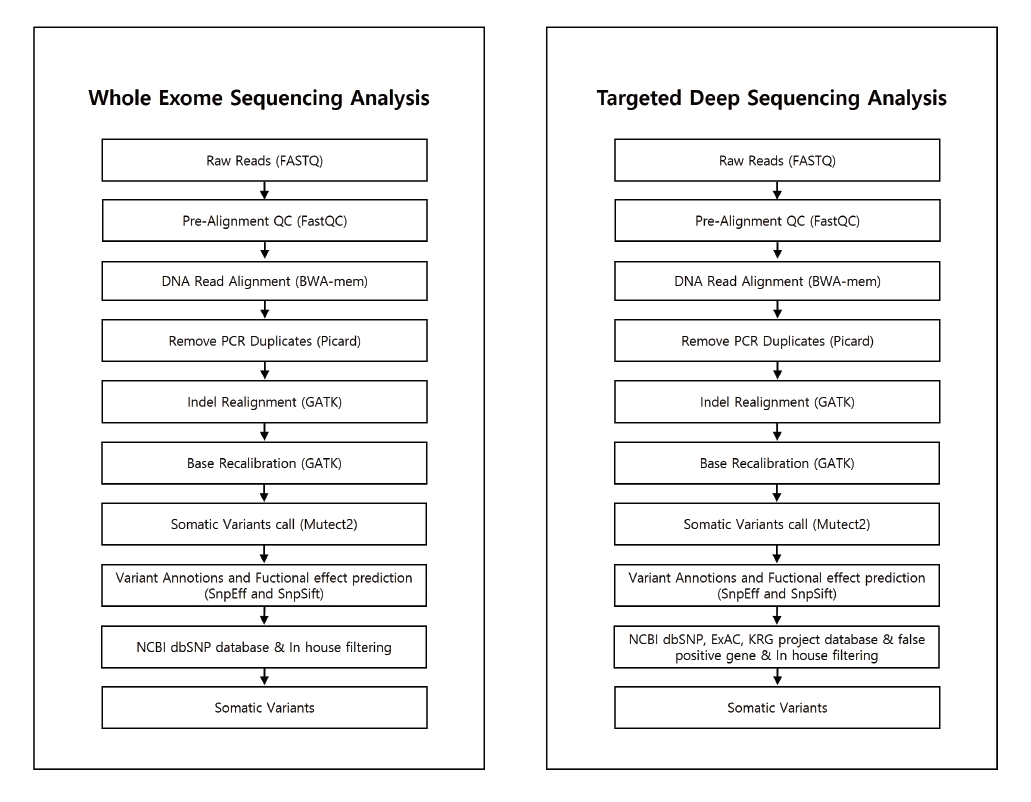

Supplement: Supplementary file 6 — Supplementary figure 4 [file 41375_2018_309_MOESM6_ESM.jpg]

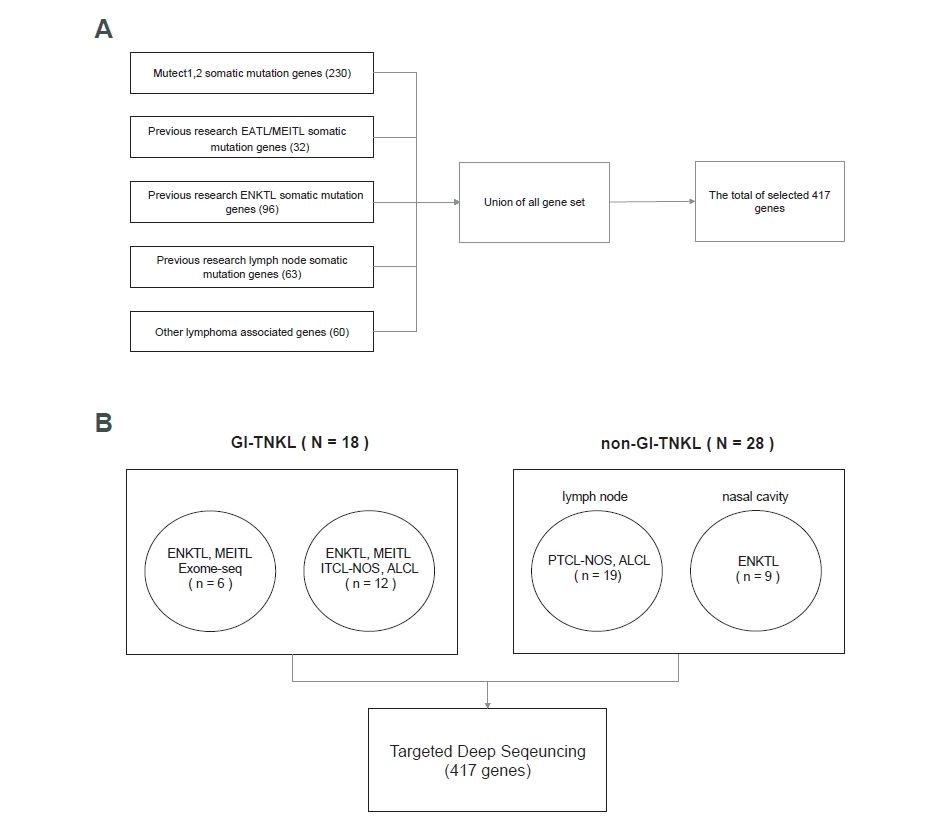

Supplement: Supplementary file 7 — Supplementary figure 5 [file 41375_2018_309_MOESM7_ESM.jpg]

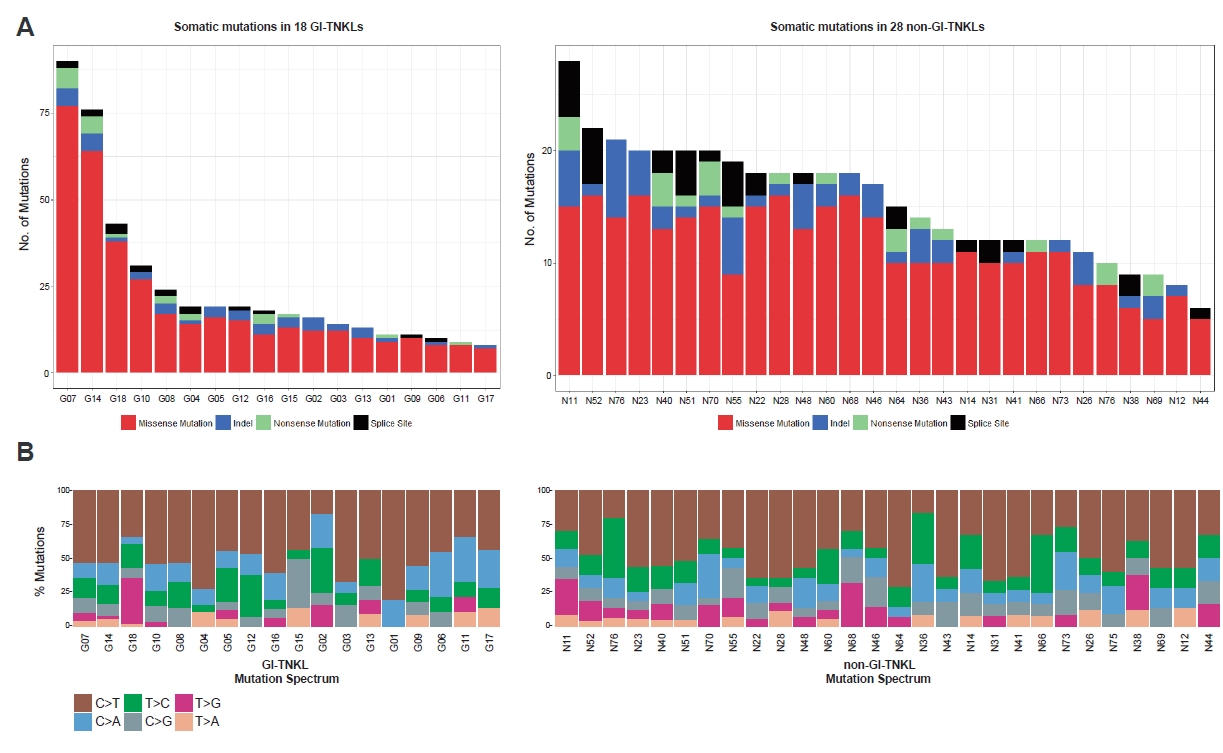

Supplement: Supplementary file 8 — Supplementary figure 6 [file 41375_2018_309_MOESM8_ESM.jpg]

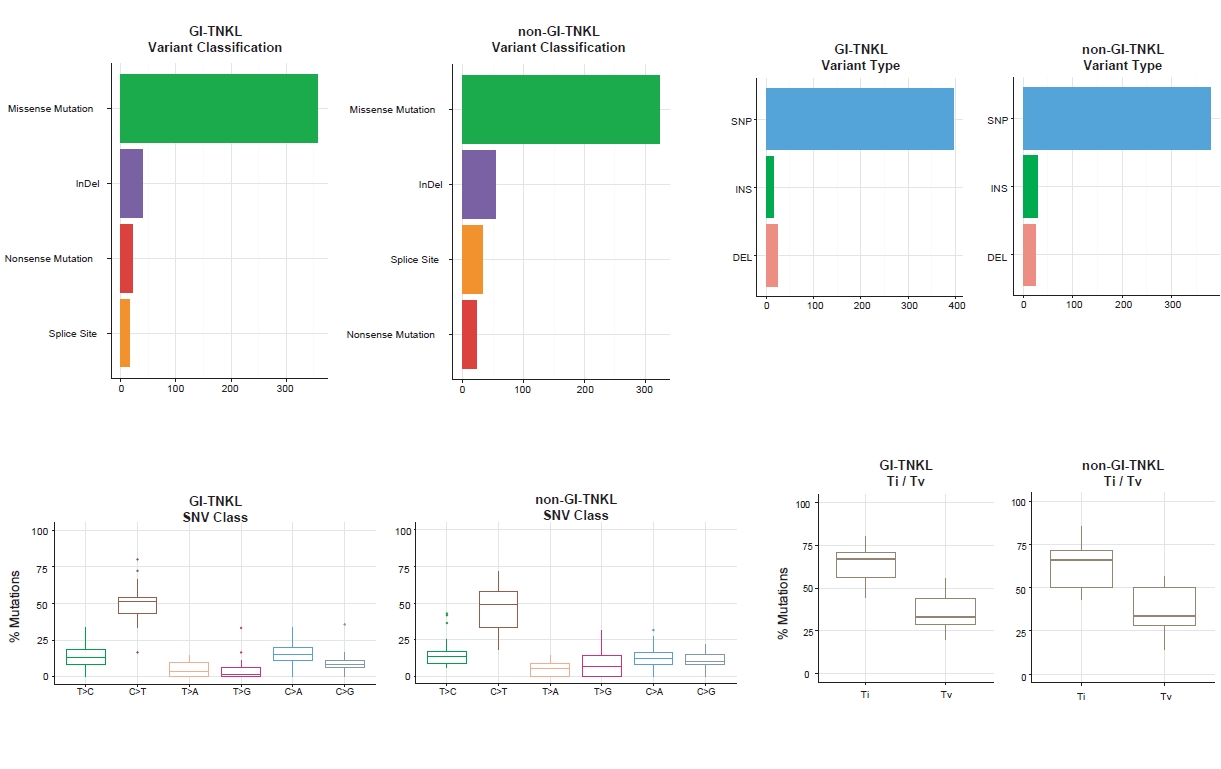

Supplement: Supplementary file 9 — Supplementary figure 7 [file 41375_2018_309_MOESM9_ESM.jpg]

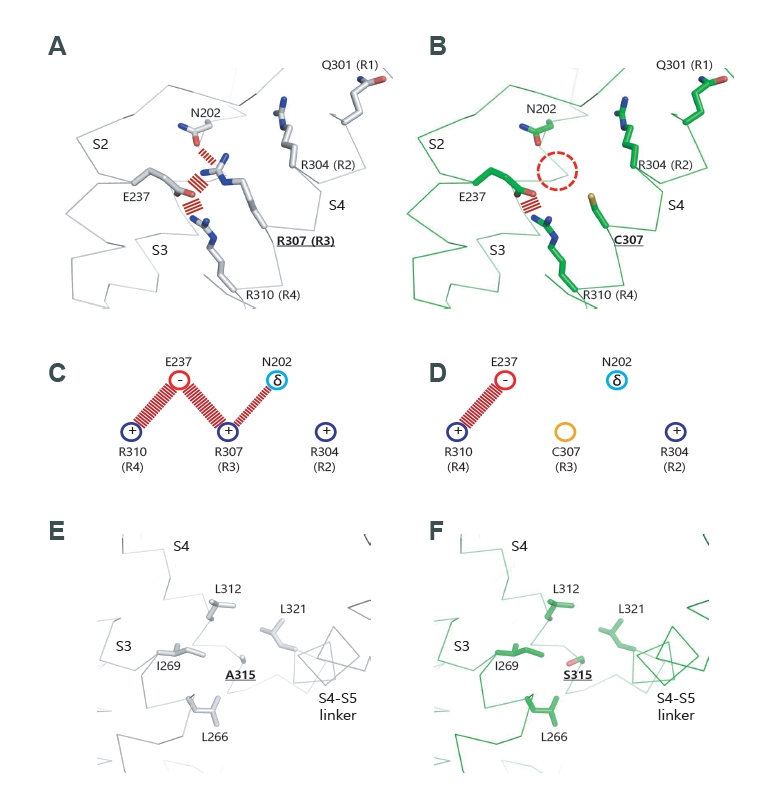

Supplement: Supplementary file 10 — Supplementary figure 8 [file 41375_2018_309_MOESM10_ESM.jpg]

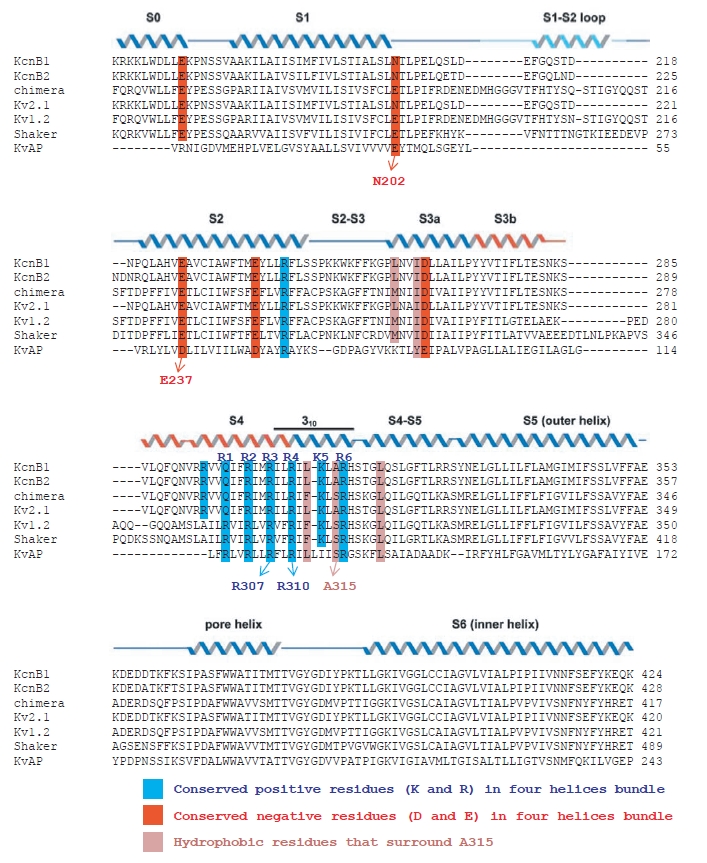

Supplement: Supplementary file 11 — Supplementary figure 9 [file 41375_2018_309_MOESM11_ESM.jpg]

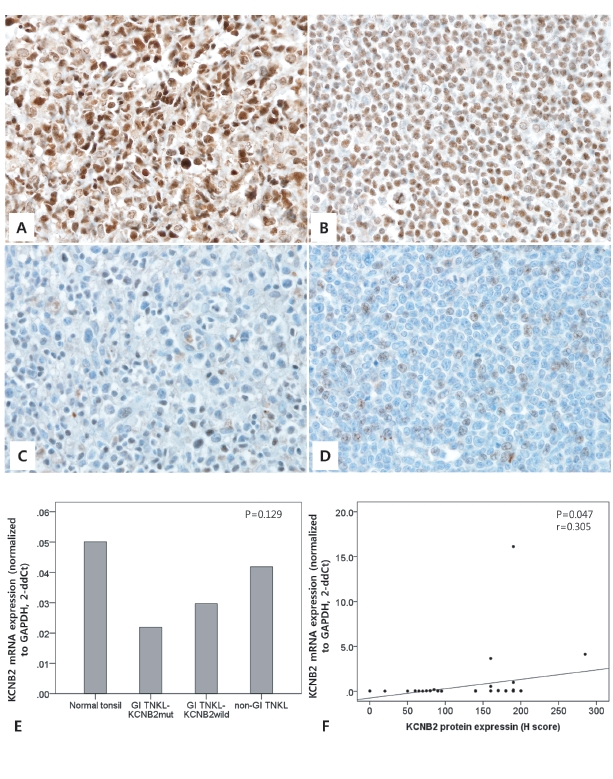

Supplement: Supplementary file 12 — Supplementary figure 10 [file 41375_2018_309_MOESM12_ESM.jpg]

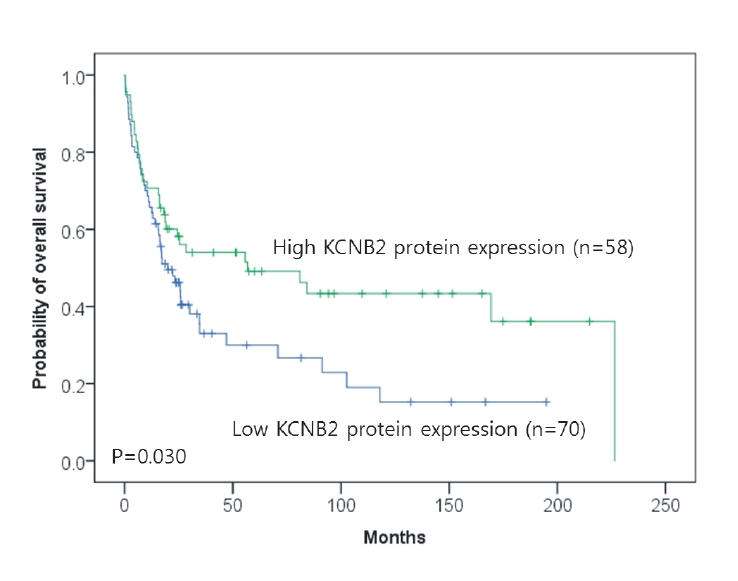

Supplement: Supplementary file 13 — Supplementary figure 11 [file 41375_2018_309_MOESM13_ESM.jpg]

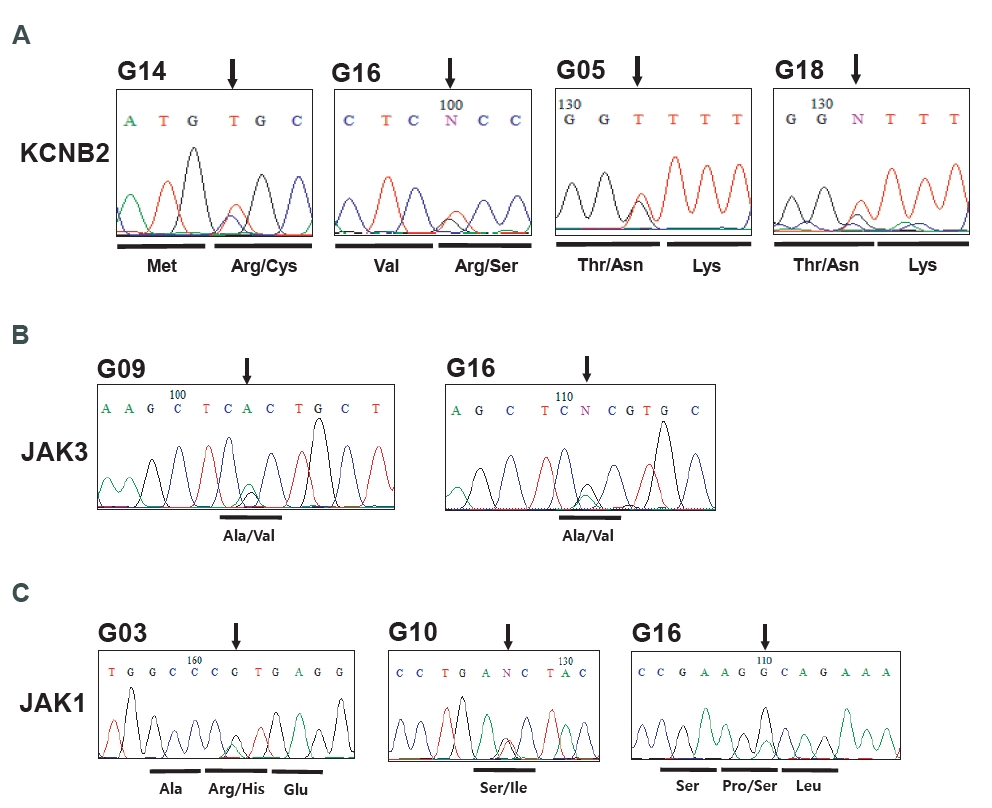

Supplement: Supplementary file 14 — Supplementary figure 12 [file 41375_2018_309_MOESM14_ESM.jpg]
