# Supplementary material for: Characteristic gene alterations in primary gastrointestinal T- and NK-cell lymphomas
Source: Leukemia. 2019 Jan 23;33(7):1797–832. doi: 10.1038/s41375-018-0309-4 (PMC6755973; doi:10.1038/s41375-018-0309-4)
Supplement: Supplementary file 15 — Supplementary table 1 [file 41375_2018_309_MOESM15_ESM.pdf]

Supplementary Table 1. Information on each patient included for sequencing analysis

Target seq ID	WES ID	Sex	Age	Primary sties	Procedure	Diagnosis
G01		M	60	Stomach	biopsy	ALCL, ALK-
G02		F	52	Colon	resection	MEITL
G03		M	46	Small bowel	resection	MEITL
G04		M	73	Small bowel	resection	ITCL-NOS
G05	10_ENKL	M	63	Small bowel	resection	ENKTL
G06		F	57	Small bowel	resection	MEITL
G07	03_EATL	M	58	Stomach	resection	MEITL
G08		F	47	Small bowel	resection	ALCL, ALK-
G09		M	59	Duodenum	biopsy	ENKTL
G10	06_EATL	M	41	Small bowel	resection	MEITL
G11		M	66	Colon	biopsy	ENKTL
G12	08_ENKL	M	36	Colon	resection	ENKTL
G13		M	50	Colon	biopsy	ITCL-NOS
G14	09_EATL	F	45	Small bowel	resection	MEITL
G15		M	75	Stomach	biopsy	ALCL, ALK-
G16		F	78	Small bowel	resection	ENKTL
G17		F	62	Stomach	biopsy	ITCL-NOS
G18	12_ENKL	M	60	Colon	resection	ENKTL
N11		M	34	Lymph node	excision	PTCL-NOS
N12		F	39	Lymph node	excision	ALCL, ALK-
N14		M	64	Lymph node	excision	PTCL-NOS
N22		M	26	Lymph node	excision	ALCL, ALK-
N23		M	63	Lymph node	excision	PTCL-NOS
N26		F	60	Lymph node	biopsy	PTCL-NOS
N28		M	58	Lymph node	excision	PTCL-NOS
N31		F	59	Lymph node	biopsy	ALCL, ALK-
N36		M	65	Lymph node	excision	PTCL-NOS
N38		M	74	Lymph node	excision	PTCL-NOS
N40		M	68	Lymph node	excision	PTCL-NOS
N41		M	70	Lymph node	excision	PTCL-NOS
N43		M	71	Lymph node	excision	PTCL-NOS
N44		M	85	Lymph node	biopsy	PTCL-NOS
N46		M	68	Lymph node	excision	ALCL, ALK-
N48		M	67	Lymph node	biopsy	PTCL-NOS
N51		M	61	Lymph node	excision	PTCL-NOS
N52		M	42	Lymph node	biopsy	ALCL, ALK-
N55		M	64	Lymph node	biopsy	ALCL, ALK-
N60		F	46	Nasal cavity	biopsy	ENKTL
N64		M	73	Nasal cavity	biopsy	ENKTL
N66		F	40	Nasal cavity	biopsy	ENKTL
N68		M	65	Nasal cavity	biopsy	ENKTL
N69		F	67	Nasal cavity	biopsy	ENKTL
N70		M	72	Nasal cavity	biopsy	ENKTL
N73		M	63	Nasal cavity	excision	ENKTL
N75		M	74	Nasal cavity	biopsy	ENKTL
N76		M	53	Nasal cavity	biopsy	ENKTL

ENKTL, extranodal NK/T cell lymphoma of nasal type; MEITL, monomorphic epitheliotropic intestinal T-cell lymphoma; ALCL, anaplastic large cell lymphoma; ALK-, ALK-negative; ALK+, ALK-positive;
ITCL-NOS, intestinal T cell lymphoma, not otherwise specified; PTCL, peripheral T cell lymphoma; NOS, not otherwise specified
