# Supplementary material for: Characteristic gene alterations in primary gastrointestinal T- and NK-cell lymphomas
Source: Leukemia. 2019 Jan 23;33(7):1797–832. doi: 10.1038/s41375-018-0309-4 (PMC6755973; doi:10.1038/s41375-018-0309-4)
Supplement: Supplementary file 16 — Supplementary table 2 [file 41375_2018_309_MOESM16_ESM.pdf]

Supplementary Table 2. Clinical and pathological features of the patients

	Targeted sequencing (n=46)[a] n (%)	Immunohistochemistry (n=131)[b] n (%)
Sex		
Male	34 (73.9%)	84 (64.1%)
Female	12 (26.1%)	47 (35.9%)
Age		
mean ± s.d. (years)	59.0 ± 13.1	56.4 ± 17.9
Location		
GI	18 (39.1%)	13 (9.9%)
LN	19 (41.3%)	78 (59.5%)
Nasal	9 (19.6%)	31 (23.7%)
Others		9 (6.9%)
Subtype[c]		
AITL		19 (14.5%)
ALCL, ALK-	9 (19.6%)	14 (10.7%)
ALCL, ALK+	0	8 (6.1%)
MEITL	6 (13.0%)	6 (4.6%)
ENKTL	15 (32.6%)	35 (26.7%)
ITCL-NOS	3 (6.5%)	2 (1.5%)
PTCL-NOS	16 (28.3%)	47 (35.9%)
Ann Arbor stage		
I-II	14 (30.4%)	33 (25.2%)
III-IV	22 (47.8%)	67 (51.1%)
Unknown	10 (21.7%)	31 (23.7%)
EBV[d]		
Negative	17 (37.0%)	37 (28.2%)
Positive	21 (45.7%)	41 (31.3%)
Unknown	8 (17.4%)	53 (40.4%)
Clinical outcome		
Alive	15 (32.6%)	47 (35.9%)
Dead	31 (67.4%)	84 (64.1%)

a. Tissue samples from these patients were used in target sequencing and RT-PCR. Cases undergoing whole exome sequencing (n=6) were included.
b. Tissue samples from these patients were used in immunohistochemical analysis. Most cases undergoing target sequencing were included in IHC, although five cases of GI-TNKL were lost due to lack of tissue.
c. AITL, angioimmunoblastic T cell lymphoma; ALCL, anaplastic large cell lymphoma; ALK-, ALK-negative; ALK+, ALK-positive; MEITL, monomorphic epitheliotropic intestinal T cell lymphoma; ENKTL, extranodal NK/T-cell lymphoma of nasal type; ITCL-NOS, intestinal T cell lymphoma, not otherwise specified; PTCL-NOS, peripheral T cell lymphoma, not otherwise specified
d. EBV positivity was defined as when ≥30% of tumor cells showed positive signals upon in situ hybridisation for EBV-encoded RNAs (EBERs).
