# Supplementary material for: Characteristic gene alterations in primary gastrointestinal T- and NK-cell lymphomas
Source: Leukemia. 2019 Jan 23;33(7):1797–832. doi: 10.1038/s41375-018-0309-4 (PMC6755973; doi:10.1038/s41375-018-0309-4)
Supplement: Supplementary file 17 — Supplementary table 3 [file 41375_2018_309_MOESM17_ESM.pdf]

Supplementary Table 3. Summary of Exome and Targeted Deep Sequencing Statistics (N=46)

Sample	Number of reads	Mapped reads	Mapped re	Coverage	Mean Mapping Quality
exome03N	81,689,287	81,524,443	99.80%	164	55.49
exome03T	152,062,915	151,416,961	99.58%	305	55.72
exome06N	67,972,848	67,871,838	99.85%	137	55.73
exome06T	165,472,278	164,944,252	99.68%	333	55.4
exome08N	71,631,724	71,523,150	99.85%	144	55.66
exome08T	125,062,249	124,511,032	99.56%	251	55.73
exome09N	73,901,486	73,789,781	99.85%	149	55.5
exome09T	146,146,309	145,536,734	99.58%	293	55.42
exome10N	74,365,695	74,214,516	99.80%	149	55.71
exome10T	126,599,746	126,059,631	99.57%	254	55.67
exome12N	74,362,945	74,214,219	99.80%	149	55.59
exome12T	140,023,939	139,380,577	99.54%	281	55.55
G01	39,674,043	39,551,909	99.69%	1182	57.41
G02	33,947,646	33,850,627	99.71%	875	57.26
G03	31,733,819	31,635,505	99.69%	847	57.22
G04	30,951,924	30,873,611	99.75%	846	57.1
G05	29,472,853	29,391,605	99.72%	844	57.27
G06	42,722,358	42,565,665	99.63%	1201	57.32
G07	33,716,528	33,607,375	99.68%	914	57.28
G08	37,429,990	37,321,105	99.71%	805	56.97
G09	29,356,206	28,985,987	98.74%	779	57.16
G10	35,099,234	35,005,603	99.73%	955	57.26
G11	39,429,428	39,305,847	99.69%	1213	57.47
G12	33,569,736	33,483,290	99.74%	915	57.29
G13	38,978,726	38,876,908	99.74%	964	57.06
G14	37,132,782	37,030,021	99.72%	988	57.33
G15	42,886,888	42,758,355	99.70%	1247	57.29
G16	34,069,643	33,973,662	99.72%	897	57.14
G17	38,694,508	38,554,164	99.64%	1106	57.27
G18	38,149,641	38,045,529	99.73%	1058	57.18
N11	35,081,798	34,991,331	99.74%	835	56.93
N12	26,260,891	26,174,865	99.67%	620	57.21
N14	27,502,721	27,413,977	99.68%	772	57.24
N22	27,185,268	27,095,954	99.67%	785	57.33
N23	30,243,771	30,144,523	99.67%	698	57.01
N26	28,060,233	27,974,934	99.70%	647	57.07
N28	28,553,189	28,419,339	99.53%	619	57.12
N31	28,958,179	28,875,058	99.71%	812	57.27
N36	28,805,690	28,715,360	99.69%	795	57.32
N38	40,542,009	40,423,709	99.71%	945	56.96
N40	30,285,692	30,205,985	99.74%	740	57.22
N41	26,001,624	25,920,079	99.69%	763	57.32
N43	28,464,850	28,366,704	99.66%	692	57.19
N44	25,694,205	25,615,702	99.69%	670	57.37
N46	42,084,895	41,927,979	99.63%	1002	57.06
N48	29,696,903	29,614,154	99.72%	718	57.18
N51	27,371,042	27,296,154	99.73%	696	57.1
N52	27,964,552	27,870,953	99.67%	626	57.12
N55	26,100,068	26,024,367	99.71%	653	57.07
N60	44,870,939	44,694,969	99.61%	880	56.89
N64	53,560,048	53,338,214	99.59%	1309	56.97
N66	30,221,014	30,071,363	99.50%	737	57.11
N68	26,812,107	26,719,748	99.66%	705	57.18
N69	52,538,437	52,361,128	99.66%	1344	57.2
N70	33,721,517	33,631,060	99.73%	932	57.24
N73	59,465,676	59,239,699	99.62%	1668	57.35
N75	30,199,779	30,037,984	99.46%	848	57.26
N76	35,099,294	35,010,937	99.75%	991	57.24
