# Supplementary material for: Characteristic gene alterations in primary gastrointestinal T- and NK-cell lymphomas
Source: Leukemia. 2019 Jan 23;33(7):1797–832. doi: 10.1038/s41375-018-0309-4 (PMC6755973; doi:10.1038/s41375-018-0309-4)
Supplement: Supplementary file 18 — Supplementary table 4 [file 41375_2018_309_MOESM18_ESM.pdf]

Supplementary Table 4. Targeted Deep Sequencing Gene List (417 Genes)

WDR64	*FRMPD3*	*AKT1*	*CTNNB1*	*PAK3*	*PRKD1*	*EPOR*	*MBD1*	*PHIP*	*ABCF3*	*MAGI2*	*MRPL40*	*FBN2*	*AKAP14*
BOC	*DSPP*	*RASA2*	*KIT*	*RBMXL3*	*EIF4A1*	*HDAC6*	*ERG*	*MUC2*	*NAA15*	*NPTX2*	*ASPHD2*	*RAB44*	*PCDHGA9*
PKD2	*PKHD1*	*NFKB1*	*FAS*	*SLC26A6*	*DDX25*	*IFNA14*	*BCR*	*ID3*	*CYP2A13*	*NRCAM*	*UPK3A*	*RFPL4B*	*CUL9*
SPINK5	*ZNF462*	*SMCHD1*	*IL2RG*	*COL24A1*	*DDX55*	*IFNA4*	*MYH9*	*MYC*	*CYP26C1*	*ZBED6CL*	*CLCN4*	*CHN2*	*DDX11*
GPRC6A	*DDX3X*	*RYR2*	*FBXW10*	*HSPA6*	*DDX60L*	*IL2RB*	*PCLO*	*GNA13*	*GRTP1*	*EPB41L4B*	*NYX*	*AVL9*	*OR6S1*
PPP1R9A	*ELF4*	*PTCHD4*	*CHEK2*	*DNAH12*	*DHX30*	*IL6R*	*ARID1B*	*BCL2*	*FUT3*	*PARD3*	*LHX4*	*MUC3A*	*CBLN3*
ANK3	*CTNNA2*	*MYO18B*	*CCNB1*	*PCDHB1*	*DHX58*	*IL7R*	*APC*	*PIM1*	*SGK223*	*TACR2*	*THSD7B*	*PURG*	*FOXB1*
FIBIN	*CD53*	*FNDC1*	*CCNB2*	*MAP3K5*	*WDR17*	*KDM5C*	*DST*	*BTG1*	*TMEM179*	*CHST3*	*AOX1*	*EYA1*	*KRTAP4-11*
TP53	*PEX16*	*DLGAP3*	*CCNG2*	*ZFHX4*	*NTRK3*	*LEP*	*BANK1*	*MEF2B*	*KCNJ12*	*CHST1*	*YIPF7*	*KCNB2*	*KRT38*
EPB41L3	*EP300*	*JAK2*	*PFAS*	*MUC4*	*TRMT12*	*LIFR*	*CSMD2*	*CD79B*	*SUCO*	*FAM181B*	*COL11A2*	*SLC24A2*	*CXADR*
JAK1	*POU4F2*	*STAT1*	*PAICS*	*OR4N2*	*CACNA2D*	*NSD1*	*TUBGCP6*	*FAT1*	*NBPF1*	*CEP164*	*DDHD2*	*TEK*	*GAB3*
FSIP2	*DMWD*	*STAT5A*	*HIST1H2A*	*RUNX1T1*	*INTS8*	*PIAS2*	*KDM6A*	*HIST1H1C*	*FCN3*	*TMPRSS1*	*IDO2*	*TDRD7*	*PROK1*
FAT4	*MEGF6*	*ATM*	*HIST1H4C*	*LZTS2*	*KCNH8*	*PIK3CB*	*TET1*	*HIST1H1E*	*FPGT-TN*	*OR8G1*	*PABPC1*	*LAMC3*	*EFCAB12*
MUC5B	*DNAH9*	*CCR4*	*ELL3*	*HECW1*	*DNAH5*	*PIK3R1*	*ARID2*	*TTN*	*FCRL3*	*NOP2*	*SCRIB*	*OR2AG2*	*HOXA6*
FAT3	*HLA-DRB*	*PRF1*	*CASP1*	*DNAJC6*	*MGAT4C*	*RERE*	*TNFAIP3*	*IDH1*	*DDX59*	*SUPT16H*	*OR5R1*	*NRXN2*	*TRAPPC9*
SLC26A9	*STAT3*	*IDH2*	*CASP4*	*DNM1*	*PTPRD*	*SUZ12*	*CHD8*	*PRRX1*	*PLEKHA6*	*CLMN*	*ACTN1*	*CADM1*	*BAIAP3*
SLC8A1	*STAT5B*	*DNMT3A*	*CASP8*	*MUC17*	*RYR3*	*ATR*	*ZAP70*	*CLTCL1*	*CAPN14*	*SLC30A4*	*AHNAK2*	*PRB1*	*CHAF1A*
NCKAP5	*JAK3*	*RHOA*	*STK17A*	*XIRP2*	*TACC2*	*FLT4*	*NF1*	*MUC1*	*SERTAD2*	*DENND4A*	*ALOX15*	*SLC41A2*	*KANK3*
SETD2	*NRAS*	*SYK*	*BIRC5*	*PREX1*	*B2M*	*SYNE1*	*TNFRSF1*	*SOS1*	*WDR12*	*CERS3*	*CCDC40*	*DNAJA2*	*HSPB6*
MAGI1	*BRAF*	*TP63*	*CCR7*	*MGA*	*CD58*	*SBDS*	*TRAF3*	*FGF23*	*PTPRG*	*DDX19B*	*ZBTB7C*	*CA5A*	*MUM1L1*
CHRM2	*TET2*	*CDKN2A*	*CNTFR*	*ASXL3*	*PIK3CG*	*FGFR1*	*FOXO1*	*FZD4*	*IGSF10*	*SLFN12*	*ZNF613*	*CDC42EP*	*GRIA3*
CSMD3	*TYK2*	*CARD11*	*IL22*	*MSN*	*PTCH1*	*IKBKB*	*BCORL1*	*PPP2R5B*	*GPR149*	*SLC14A2*	*RBFOX2*	*RASAL3*	*MIR17HG*
KRAS	*SOCS1*	*PLCG1*	*IL21*	*MGAM*	*PTGS2*	*KAT6B*	*SETBP1*	*RP1L1*	*HRG*	*RBFA*	*NBPF10*	*CYP4F12*	*TNFSF10*
PCDH17	*FYN*	*ARID1A*	*TNFRSF8*	*CHPF2*	*HOXA10*	*KMT2D*	*FBXW7*	*NCAPD3*	*NWD2*	*HNRNPM*	*KIRREL*	*C19orf68*	*KMT2A*
MYH4	*NOTCH1*	*SMARCA4*	*BATF3*	*ATP10B*	*PTPN11*	*BCL11B*	*COL6A3*	*ATP2B1*	*AMTN*	*PTGER1*	*IGSF9*	*KIR2DS4*	*NBN*
SLC39A11	*EZH2*	*PRDM1*	*TMOD1*	*CIITA*	*DSG1*	*THBS1*	*LRRK1*	*ADSSL1*	*GUCY1A3*	*EMR2*	*XCL2*	*KIAA1755*	*NLRP2*
ADAM33	*CD247*	*ATG5*	*CD28*	*LMCD1*	*ANKRD30*	*MYH11*	*XRCC6BP*	*BRD7*	*MAP7*	*CD22*	*CYBRD1*	*RTEL1*	*KMT2B*
POF1B	*GNAI2*	*AIM1*	*MROH9*	*RGS7*	*BCOR*	*CDH11*	*ALPP*	*FUT6*	*WDR27*	*FCGBP*	*ITIH1*	*CLDN8*	
ROBO2	*CREBBP*	*HACE1*	*NCOA2*	*CHST10*	*MLL3*	*PER1*	*EIF3A*	*ZNF708*	*SNX13*	*ZSCAN4*	*FBXL5*	*TMEM47*	
MICALCL	*SH2B3*	*FOXO3*	*VPS13C*	*COL4A1*	*STAT6*	*ZNF521*	*KIF7*	*RHOQ*	*RAPGEF5*	*BANF2*	*ANP32C*	*NRK*	
