# Supplementary material for: Characteristic gene alterations in primary gastrointestinal T- and NK-cell lymphomas
Source: Leukemia. 2019 Jan 23;33(7):1797–832. doi: 10.1038/s41375-018-0309-4 (PMC6755973; doi:10.1038/s41375-018-0309-4)
Supplement: Supplementary file 19 — Supplementary table 5 [file 41375_2018_309_MOESM19_ESM.pdf]

Supplementary Table 5. False Positive Gene List

FLAGS TOP 100 Genes	MutSigCV	MassGenomomis
TTN	*OR2G6*	*LOC*
MUC16	*OR4C6*	*ENS*
OBSCN	*OR4M2*	*FAM*
AHNAK2	*OR5L2*	*GOL*
SYNE1	*OR2T4*	*PRA*
FLG	*OR5D18*	*NBP*
MUC5B	*OR4A15*	*POT*
DNAH17	*OR6F1*	*DEF*
PLEC	*OR2T33*	*OR2*
DST	*OR4S2*	*MUC*
SYNE2	*OR11L1*	*KRT*
NEB	*OR4M1*	*WAS*
HSPG2	*OR5T1*	*ANK*
LAMA5	*OR8J3*	*TRI*
AHNAK	*OR51B2*	*OR1*
HMCN1	*OR8H2*	*FRG*
USH2A	*OR9G9*	
DNAH11	*OR4N2*	
MACF1	*OR10G9*	
MUC17	*OR5I1*	
DNAH5	*OR14A16*	
GPR98	*OR2M2*	
FAT1	*OR5B12*	
PKD1	*OR5M9*	
MDN1	*OR4C11*	
RNF213	*OR1C1*	
RYR1	*OR4N4*	
DNAH2	*OR5J2*	
DNAH3	*OR2G3*	
DNAH8	*OR10G8*	
DNAH1	*OR5W2*	
DNAH9	*OR2T3*	
ABCA13	*OR10AG1*	
APOB	*OR4K1*	
SRRM2	*OR2M7*	
CUBN	*OR4C12*	
SPTBN5	*OR4D5*	
PKHD1	*OR2T1*	
LRP2	*OR5F1*	
FBN3	*OR2T8*	
CDH23	*OR4C13*	
DNAH10	*OR5K1*	
FAT4	*OR4K5*	
RYR3	*OR2B11*	
PKHD1L1	*OR5L1*	

FAT2	*OR2L8*
CSMD1	*OR2T12*
PCNT	*OR2T34*
COL6A3	*OR8H1*
FRAS1	*OR5D16*
FCGBP	*OR10Q1*
DNAH7	*OR2M3*
RP1L1	*OR6K3*
PCLO	*OR5T3*
ZFHX3	*OR14C36*
COL7A1	*OR5AC2*
LRP1B	*OR52J3*
FAT3	*OR4Q3*
EPPK1	*OR10A4*
VPS13C	*OR4C16*
HRNR	*OR8B2*
MKI67	*OR5D14*
MYO15A	*OR5H6*
STAB1	*OR8I2*
ZAN	*OR2T2*
UBR4	*OR4A16*
VPS13B	*OR52E6*
LAMA1	*OR6N1*
XIRP2	*OR2AK2*
BSN	*OR2L2*
KMT2C	*OR4D11*
ALMS1	*OR2A5*
CELSR1	*OR51S1*
TG	*OR9A2*
LAMA3	*OR51L1*
DYNC2H1	*OR56A4*
KMT2D	*OR52E2*
BRCA2	*OR6M1*
CMYA5	*OR2T11*
SACS	*OR5M11*
STAB2	*OR4C46*
AKAP13	*OR6K2*
UTRN	*OR2B3*
VWF	*OR2T6*
VPS13D	*OR56A1*
ANK3	*OR5B2*
FREM2	*OR4K15*
PKD1L1	*OR5AS1*
LAMA2	*OR8A1*
ABCA7	*OR4C3*
LRP1	*OR4D2*
ASPM	*OR8K3*
MYOM2	*OR8J1*
PDE4DIP	*OR4F6*

TACC2	*OR8H3*
MUC2	*OR1J4*
TEP1	*OR52A5*
HELZ2	*OR8B4*
HERC2	*OR51I1*
ABCA4	
