# Supplementary material for: Characteristic gene alterations in primary gastrointestinal T- and NK-cell lymphomas
Source: Leukemia. 2019 Jan 23;33(7):1797–832. doi: 10.1038/s41375-018-0309-4 (PMC6755973; doi:10.1038/s41375-018-0309-4)
Supplement: Supplementary file 20 — Supplementary table 6 [file 41375_2018_309_MOESM20_ESM.pdf]

Supplementary Table 6. GI-TNKL Somatic Mutations

Patient ID	Chromosome	Start	End	Ref	Alt	Cosmic	Variant allele frequency	Mutation type	Transcription ID	Gene	Amino acid change	SnpEff Impact	MutationTaster_pred	PROVEAN_pred	Polyphen2_HDIV_pred	Polyphen2_HVAR_pred	SIFT_pred	FILTER_ExAC	AF_TOTAL	AF_AFR	AF_AMR	AF_EAS	AF_FIN	AF_NFE	AF_SAS	AF_OTH	AF_KoreaRef
G14*	chr3	183908812	183908812	GA	G	.	0.439	Indel	NM_018358.2	ABCF3	p.Glu473fs	HIGH															
G10*	chr14	69341590	69341590	C	T	COSM46591	0.181	Missense_Mutation	NM_001130004.1	ACTN1	p.Glu911Lys	MODERATE	D	D	D	P	D	.	.	.	.	.	.	.	.	.	.
G14*	chr20	3655249	3655249	G	A	COSM37995	0.583	Missense_Mutation	NM_025220.4	ADAM33	p.Arg168Trp	MODERATE	N	D	B	B	D	PASS	2.48E-05	0	8.65E-05	0	0	3.01E-05	0	0.00111	.
G07*	chr19	14875409	14875409	G	A	.	0.250	Missense_Mutation	NM_013447.3	ADGRE2	p.Ala307Val	MODERATE															
G02	chr14	105208272	105208272	T	C		0.475	Missense_Mutation	NM_199165.2	ADSSL1	p.Val337Ala	MODERATE	D	D	D	D	D	PASS	1.66E-05	0	0	0.000232	0	0	0	0	.
G07*	chr14	105207521	105207521	G	GC	COSM37348	0.228	Indel	NM_199165.2	ADSSL1	p.Lys291fs	HIGH															
G01	chr6	106967902	106967902	G	A	COSM38572	0.442	Missense_Mutation	NM_001624.3	AIM1	p.Arg532His	MODERATE	N	N	B	B	T	PASS	0.0005523	0.001058	0.000173	0.005086	0	0.00015	0	0.00221	0.00273
G15	chr6	106991468	106991468	G	A		0.518	Missense_Mutation	NM_001624.3	AIM1	p.Gly1271Ser	MODERATE	D	D	D	D	D	PASS	0.0001319	0	0	0.001733	0	0	0	0	0.00455
G14*	chr6	106991468	106991468	G	A		0.452	Missense_Mutation	NM_001624.3	AIM1	p.Gly1271Ser	MODERATE	D	D	D	D	D	PASS	0.0001319	0	0	0.001733	0	0	0	0	0.00455
G14*	chrX	119037346	119037346	A	T		0.403	Missense_Mutation	NM_178813.5	AKAP14	p.Ile54Phe	MODERATE	N	N	B	B	T	.	.	.	.	.	.	.	.	.	.
G06	chr14	105239419	105239419	T	C	COSM17487	0.455	Missense_Mutation	NM_001014431.1	AKT1	p.Asp323Gly	MODERATE	D	D	P	P	T	.	.	.	.	.	.	.	.	.	.
G08	chr4	71396910	71396910	G	A		0.544	Missense_Mutation	NM_212557.3	AMTN	p.Arg171His	MODERATE	N	N	B	B	T	PASS	0.0002891	9.64E-05	8.66E-05	0.002568	0	0.000165	0	0.00112	0.00455
G07*	chr4	71396769	71396769	C	T	.	0.233	Missense_Mutation	NM_212557.3	AMTN	p.Thr124Met	MODERATE															
G06	chr18	14828289	14828289	C	T		0.373	Missense_Mutation	NM_001145029.1	ANKRD30B	p.Ser800Phe	MODERATE	N	N	D	D	T	PASS	0.0001031	0	0	0.001613	0	0	0.000127	0	0.00455
G07*	chr18	14779986	14779986	G	A	COSM13035	0.214	Missense_Mutation	NM_001145029.1	ANKRD30B	p.Arg483Gln	MODERATE															
G14*	chr2	242142778	242142778	C	T	.	0.286	Nonsense_Mutation	NM_001001891.3	ANO7	p.Gln306*	HIGH															
G14*	chr4	165118829	165118829	C	T	COSM29620	0.291	Missense_Mutation	NM_012403.1	ANP32C	p.Arg12Gln	MODERATE	.	.	.	.	.	.	.	.	.	.	.	.	.	.	.
G10*	chr2	201515695	201515695	A	C		0.378	Splice_Site	NM_001159.3	AOX1		HIGH	D	.	.	.	.	.	.	.	.	.	.	.	.	.	.
G07*	chr6	157100591	157100591	A	G		0.423	Missense_Mutation	NM_020732.3	ARID1B	p.Met510Val	MODERATE	N	N	B	B	T	PASS	6.93E-05	0	0	0.000634	0	0	9.83E-05	0	0.00045
G18*	chr6	157527451	157527451	G	A		0.474	Missense_Mutation	NM_020732.3	ARID1B	p.Asp1726As	MODERATE	D	N	P	B	T	PASS	5.77E-05	0	0	0.000809	0	0	0	0	0.00136
G01	chr12	46244661	46244661	C	T		0.377	Nonsense_Mutation	NM_152641.2	ARID2	p.Gln919*	HIGH	A	.	.	.	.	.	.	.	.	.	.	.	.	.	.
G07*	chr22	26830365	26830365	G	A		0.436	Missense_Mutation	NM_020437.4	ASPHD2	p.Gly262Arg	MODERATE	D	N	D	D	T	.	.	.	.	.	.	.	.	.	.
G04	chr18	31318494	31318494	C	T		0.423	Missense_Mutation	NM_030632.2	ASXL3	p.Pro376Ser	MODERATE	N	N	P	B	D	.	.	.	.	.	.	.	.	.	.
G09	chr11	108163382	108163382	C	G		0.701	Missense_Mutation	NM_000051.3	ATM	p.Phe1491Le	MODERATE	N	N	B	B	T	.	.	.	.	.	.	.	.	.	.
G16	chr5	159992486	159992486	G	A		0.398	Nonsense_Mutation	NM_025153.2	ATP10B	p.Arg1454*	HIGH	D	.	.	.	.	PASS	3.32E-05	0.000102	0	0	0	3.00E-05	6.07E-05	0	.
G07*	chr12	89984839	89984839	G	GCCCAGGTTTTCTTCTA		0.193	Indel	NM_001682.2	ATP2B1	p.Leu1196fs	HIGH	.	.	.	.	.	.	.	.	.	.	.	.	.	.	.
G14*	chr7	32584393	32584393	T	G		0.486	Splice_Site	NM_015060.2	AVL9		HIGH	D	.	.	.	.	.	.	.	.	.	.	.	.	.	.
G16	chr15	45003780	45003780	ACT	A	COSM14457	0.450	Indel	NM_004048.2	B2M	p.Leu15fs	HIGH	.	.	.	.	.	.	.	.	.	.	.	.	.	.	.
G18*	chr15	45007887	45007887	ATAGT	A		0.219	Indel	NM_004048.2	B2M	p.Val113fs	HIGH	.	.	.	.	.	.	.	.	.	.	.	.	.	.	.
G18*	chr16	1388628	1388628	G	T		0.464	Missense_Mutation	NM_003933.4	BAIAP3	p.Glu61Asp	MODERATE	N	N	B	B	T	PASS	1.47E-05	0	0	0.000207	0	0	0	0	.
G18*	chr16	1395359	1395359	G	C		0.211	Missense_Mutation	NM_003933.4	BAIAP3	p.Val719Leu	MODERATE	D	N	P	B	T	.	.	.	.	.	.	.	.	.	.
G07*	chr20	17705785	17705785	G	T		0.393	Missense_Mutation	NM_001159495.1	BANF2	p.Gly46Cys	MODERATE	D	D	D	D	D	PASS	8.24E-06	0	0	0	0	1.50E-05	0	0	.
G17	chr14	99642373	99642373	G	A		0.483	Missense_Mutation	NM_138576.3	BCL11B	p.Pro267Leu	MODERATE	D	D	D	D	D	.	.	.	.	.	.	.	.	.	.
G15	chrX	39921444	39921444	T	C	COSM16510	0.372	Missense_Mutation	NM_001123385.1	BCOR	p.Asn1459Se	MODERATE	D	.	.	.	.	PASS	1.90E-05	0.000177	0	0	0	0	0	0	.
G02	chrX	39933661	39933661	GGCT	G		0.456	Indel	NM_001123385.1	BCOR	p.Gln312del	MODERATE	.	.	.	.	.	PASS	0.0002779	0	0	0.003508	0	0	0	0	.
G16	chrX	39933072	39933072	C	T		0.400	Nonsense_Mutation	NM_001123385.1	BCOR	p.Trp509*	HIGH	A	.	.	.	.	.	.	.	.	.	.	.	.	.	.
G18*	chr22	23523764	23523764	A	G		0.172	Missense_Mutation	NM_004327.3	BCR	p.Gln206Arg	MODERATE	D	N	D	P	D	.	.	.	.	.	.	.	.	.	.
G07*	chr3	112998269	112998269	C	T	COSM31186	0.564	Missense_Mutation	NM_001301861.1	BOC	p.Arg664Trp	MODERATE	D	D	D	D	D	PASS	1.66E-05	9.87E-05	0	0	0	1.51E-05	0	0	.
G07*	chr16	50353883	50353883	GATATCAC	G		0.248	Indel	NM_001173984.2	BRD7	p.Thr602_Ile6	MODERATE	.	.	.	.	.	.	.	.	.	.	.	.	.	.	.
G12*	chr19	48697974	48697974	C	A		0.477	Missense_Mutation	NM_199341.3	C19orf68	p.Thr218Asn	MODERATE	N	N	D	P	D	PASS	0.0001716	0	0	0.005155	0	0.000307	0	0	.
G14*	chr19	48698904	48698904	T	C	.	0.491	Missense_Mutation	NM_199341.3	C19orf68	p.Leu528Ser	MODERATE															
G04	chr16	87960553	87960553	T	A		0.490	Splice_Site	NM_001739.1	CA5A		HIGH	D	.	.	.	.	.	.	.	.	.	.	.	.	.	.
G14*	chr16	87921785	87921785	G	A	.	0.408	Missense_Mutation	NM_001739.1	CA5A	p.Arg290Trp	MODERATE															
G14*	chr7	81695822	81695822	T	C		0.544	Missense_Mutation	NM_000722.3	CACNA2D1	p.Asn226Ser	MODERATE	N	N	B	B	T	PASS	5.04E-05	0	0	0.000703	0	0	0	0	0.00045
G13	chr11	115061638	115061638	C	T		0.500	Missense_Mutation	NM_001301043.1	CADM1	p.Ala379Thr	MODERATE	D	N	.	.	T	PASS	3.87E-05	0	0.000134	0	0.00021	2.41E-05	0	0.00175	.
G18*	chr11	115047223	115047223	A	C		0.153	Missense_Mutation	NM_001301043.1	CADM1	p.Ser463Ala	MODERATE	D	.	.	.	.	.	.	.	.	.	.	.	.	.	.
G14*	chr11	115102187	115102187	C	T	COSM43823	0.456	Missense_Mutation	NM_001301043.1	CADM1	p.Asp150Asn	MODERATE															
G07*	chr2	31420155	31420155	G	A		0.533	Missense_Mutation	NM_001145122.1	CAPN14	p.Pro279Ser	MODERATE	D	D	D	D	D	.	.	.	.	.	.	.	.	.	.
G12*	chr14	24898052	24898052	C	T	.	0.148	Missense_Mutation	NM_001039771.2	CBLN3	p.Gly70Glu	MODERATE															
G04	chr17	78064013	78064013	A	T		0.702	Nonsense_Mutation	NM_001243342.1	CCDC40	p.Lys970*	HIGH	N	.	.	.	.	.	.	.	.	.	.	.	.	.	.
G17	chr17	78063997	78063997	C	A		0.498	Missense_Mutation	NM_001243342.1	CCDC40	p.His964Gln	MODERATE	N	N	.	.	T	.	.	.	.	.	.	.	.	.	.
G05*	chr17	78064146	78064146	G	A	COSM49890	0.133	Missense_Mutation	NM_001243342.1	CCDC40	p.Arg1014Ly	MODERATE	N	N	.	.	D	.	.	.	.	.	.	.	.	.	.
G17	chr17	78069105	78069105	G	A		0.441	Missense_Mutation	NM_017950.3	CCDC40	p.Arg959His	MODERATE	D	D	B	B	T	PASS	2.51E-05	0.000103	0	0.000116	0	1.52E-05	0	0	.
G08	chr15	59399552	59399552	G	A		0.172	Missense_Mutation	NM_004701.3	CCNB2	p.Gly19Glu	MODERATE	N	N	B	B	T	.	.	.	.	.	.	.	.	.	.
G09	chr15	59406644	59406644	G	T		0.832	Splice_Site	NM_004701.3	CCNB2	p.Gly90Val	MODERATE	N	N	B	B	T	.	.	.	.	.	.	.	.	.	.
G07*	chr19	35837063	35837063	G	T		0.462	Missense_Mutation	NM_001771.3	CD22	p.Glu779Asp	MODERATE	N	N	P	B	T	.	.	.	.	.	.	.	.	.	.
G18*	chr2	204594421	204594421	T	C		0.119	Missense_Mutation	NM_006139.3	CD28	p.Trp154Arg	MODERATE	D	D	D	D	D	.	.	.	.	.	.	.	.	.	.
G14*	chr1	111441753	111441753	GGATGTCC	G		0.407	Indel	NM_000560.3	CD53	p.Met200_Al	MODERATE	.	.	.	.	.	.	.	.	.	.	.	.	.	.	.
G18*	chr1	117087139	117087139	T	G		0.279	Missense_Mutation	NM_001779.2	CD58	p.Glu53Ala	MODERATE	.	.	.	.	.	.	.	.	.	.	.	.	.	.	.
G18*	chr17	71282453	71282453	C	T		0.497	Missense_Mutation	NM_012121.4	CDC42EP4	p.Glu63Lys	MODERATE	D	N	P	B	D	PASS	1.66E-05	0	0	0.000116	0	1.51E-05	0	0	.
G14*	chr17	71282610	71282610	G	T		0.434	Missense_Mutation	NM_012121.4	CDC42EP4	p.Ser10Arg	MODERATE	D	N	P	P	T	.	.	.	.	.	.	.	.	.	.
G08	chr16	65038679	65038679	G	A		0.396	Missense_Mutation	NM_001797.3	CDH11	p.Arg32Trp	MODERATE	D	N	D	P	D	PASS	9.10E-05	0.000337	0	0	0	8.94E-05	8.47E-05	0	0.00045
G07*	chr11	117263745	117263745	G	T		0.455	Missense_Mutation	NM_014956.4	CEP164	p.Arg840Leu	MODERATE	D	D	D	D	D	PASS	0.0001813	0	8.65E-05	0	0	0.000315	0	0.0011	.
G07*	chr11	117267311	117267311	G	A		0.550	Missense_Mutation	NM_014956.4	CEP164	p.Asp1088As	MODERATE	N	N	P	P	D	PASS	8.25E-06	0	8.65E-05	0	0	0	0	0.00111	.
G10*	chr4	175223294	175223294	TCATAGC	T	.	0.111	Indel	NM_001145314.1	CEP44	p.Ile66_Ala67	MODERATE															
G07*	chr15	100942983	100942983	C	G		0.409	Missense_Mutation	NM_001290341.2	CERS3	p.Asp374His	MODERATE	N	N	D	B	T	.	.	.	.	.	.	.	.	.	.
G16	chr19	4409194	4409194	ACAATAA	A		0.423	Indel	NM_005483.2	CHAF1A	p.Leu136fs	HIGH	.	.	.	.	.	.	.	.	.	.	.	.	.	.	.
G18*	chr19	4433151	4433151	G	C		0.198	Missense_Mutation	NM_005483.2	CHAF1A	p.Arg763Pro	MODERATE	D	D	D	P	D	.	.	.	.	.	.	.	.	.	.
G11	chr19	4433351	4433351	C	T		0.111	Nonsense_Mutation	NM_005483.2	CHAF1A	p.Gln830*	HIGH	A	.	.	.	.	.	.	.	.	.	.	.	.	.	.
G08	chr14	21854022	21854022	G	T	COSM29646	0.111	Missense_Mutation	NM_001170629.1	CHD8	p.Pro2499His	MODERATE	N	N	B	B	T	.	.	.	.	.	.	.	.	.	.
G06	chr14	21860015	21860015	CCTT	C	COSM20299	0.503	Indel	NM_001170629.1	CHD8	p.Lys2287del	MODERATE	.	.	.	.	.	PASS	4.98E-05	0	0	0.000696	0	0	0	0	.
G14*	chr7	29440251	29440251	C	T		0.421	Missense_Mutation	NM_001293069.1	CHN2	p.Thr203Ile	MODERATE	D	N	P	D	D	PASS	8.25E-06	0	0	0	0	0	6.06E-05	0	.
G14*	chr7	136699836	136699836	T	C		0.663	Missense_Mutation	NM_000739.2	CHRM2	p.Phe75Ser	MODERATE	D	D	P	B	D	.	.	.	.	.	.	.	.	.	.
G07*	chr11	45672248	45672248	C	T		0.377	Missense_Mutation	NM_003654.5	CHST1	p.Val76Met	MODERATE	D	N	D	D	D	.	.	.	.	.	.	.	.	.	.
G07*	chr10	73767321	73767321	G	A	COSM45961	0.657	Missense_Mutation	NM_004273.4	CHST3	p.Gly178Ser	MODERATE	D	N	D	D	T	.	.	.	.	.	.	.	.	.	.
G10*	chr16	10989244	10989244	A	G		0.405	Missense_Mutation	NM_001286402.1	CIITA	p.Gln53Arg	MODERATE	N	N	B	B	T	.	.	.	.	.	.	.	.	.	.
G07*	chrX	10174530	10174530	G	A	.	0.128	Missense_Mutation	NM_001830.3	CLCN4	p.Val230Met	MODERATE															
G14*	chr21	31588106	31588106	G	T		0.324	Missense_Mutation	NM_199328.2	CLDN8	p.Phe46Leu	MODERATE	N	N	B	B	T	.	.	.	.	.	.	.	.	.	.
G09	chr14	95669250	95669250	T	A		0.378	Missense_Mutation	NM_024734.3	CLMN	p.Glu812Asp	MODERATE	N	N	B	B	T	PASS	1.74E-05	0	0	0.000116	0	0	8.28E-05	0	.
G07*	chr14	95688074	95688074	C	T		0.359	Missense_Mutation	NM_024734.3	CLMN	p.Arg93Gln	MODERATE	D	N	D	D	D	PASS	4.12E-05	0	0.000173	0	0	0	0.000182	0.00221	.
G11	chr22	19209588	19209588	A	T		0.469	Missense_Mutation	NM_007098.3	CLTCL1	p.Ile816Asn	MODERATE	N	D	P	P	D	PASS	7.45E-05	0	0	0.001043	0	0	0	0	0.00091

G10*	chr22	19220073	19220073	T	C		0.337	Missense_Mutation	NM_007098.3	CLTCL1	p.Ile524Val	MODERATE	N	N	B	B	T	.	.	.	.	.	.	.	.	.	.
G18*	chr22	19196578	19196578	G	A		0.455	Missense_Mutation	NM_007098.3	CLTCL1	p.Ala1099Va	MODERATE	D	D	D	D	D	PASS	0.0001635	0	0.000152	0.00035	0	0.000228	0	0.00162	0.00182
G15	chr9	34552265	34552265	C	G		0.522	Missense_Mutation	NM_001207011.1	CNTFR	p.Gly338Arg	MODERATE	.	N	P	B	T	PASS	0.0001562	0	0	0.0053	0	0	0	0	.
G03	chr6	33138141	33138141	G	T		0.456	Missense_Mutation	NM_080680.2.6	COL11A2.4	p.Pro1182Th	MODERATE	D	D	D	D	D	.	.	.	.	.	.	.	.	.	.
G10*	chr6	33156206	33156206	C	T		0.187	Missense_Mutation	NM_080680.2.6	COL11A2.4	p.Arg180His	MODERATE	N	N	B	B	T	PASS	6.89E-05	0.000109	0	0	0	9.48E-05	6.15E-05	0	.
G03	chr1	86210393	86210393	C	T		0.445	Missense_Mutation	NM_152890.5	COL24A1	p.Arg1543Gl	MODERATE	.	.	.	.	.	PASS	0.000174	0	0.000778	0.001044	0	1.50E-05	6.06E-05	0.01002	0.00182
G18*	chr1	86200556	86200556	G	A		0.465	Missense_Mutation	NM_152890.5	COL24A1	p.Thr1625Ile	MODERATE	.	.	.	.	.	PASS	0.0003478	0	0	0.004521	0	4.50E-05	0	0	0.00636
G14*	chr1	86249804	86249804	C	T		0.429	Missense_Mutation	NM_152890.5	COL24A1	p.Gly1405Ser	MODERATE	.	.	.	.	.	.	.	.	.	.	.	.	.	.	.
G03	chr16	3843601	3843601	A	AATTCCCACTG		0.390	Indel	NM_004380.2	CREBBP	p.Val335fs	HIGH	.	.	.	.	.	.	.	.	.	.	.	.	.	.	.
G18*	chr16	3900622	3900622	T	G		0.108	Missense_Mutation	NM_004380.2	CREBBP	p.Gln158His	MODERATE	D	N	B	B	T	.	.	.	.	.	.	.	.	.	.
G01	chr1	34190962	34190962	G	A		0.453	Missense_Mutation	NM_001281956.1	CSMD2	p.Leu895Phe	MODERATE	D	N	P	P	D	PASS	0.0001567	0	0	0.002197	0	0	0	0	0.00364
G01	chr1	34554704	34554704	G	A		0.492	Missense_Mutation	NM_001281956.1	CSMD2	p.Thr93Met	MODERATE	D	N	D	D	D	PASS	4.95E-05	0	0	0.000231	0.00015	4.50E-05	0	0	0.00136
G14*	chr1	33985195	33985195	C	T		0.622	Missense_Mutation	NM_001281956.1	CSMD2	p.Asp3607As	MODERATE	D	D	D	P	D	PASS	0.0001318	0	0	0.001387	0	4.50E-05	0	0	0.00545
G14*	chr8	113241040	113241040	C	A	.	0.294	Missense_Mutation	NM_198123.1	CSMD3	p.Val3637Leu	MODERATE															
G12*	chr2	80136918	80136918	A	C	COSM54153	0.135	Missense_Mutation	NM_001282597.2	CTNNA2	p.Asn351His	MODERATE															
G10*	chr2	80136918	80136918	A	C	COSM54153	0.110	Missense_Mutation	NM_001282597.2	CTNNA2	p.Asn351His	MODERATE															
G08	chr6	43153850	43153850	G	C		0.589	Missense_Mutation	NM_015089.3	CUL9	p.Ser303Thr	MODERATE	D	N	B	B	T	PASS	8.24E-05	0	0	0.00104	0	1.50E-05	0	0	0.00136
G17	chr6	43171290	43171290	A	G		0.520	Missense_Mutation	NM_015089.3	CUL9	p.Ile1329Val	MODERATE	D	N	P	B	T	PASS	0.000174	0	0	0.002431	0	0	0	0	0.00591
G12*	chr21	18919404	18919404	G	A	.	0.116	Missense_Mutation	NM_001338.4	CXADR	p.Glu35Lys	MODERATE															
G14*	chr2	172411097	172411097	G	A		0.448	Missense_Mutation	NM_001127383.1	CYBRD1	p.Asp138Asn	MODERATE	D	N	B	B	D	.	.	.	.	.	.	.	.	.	.
G15	chr10	94828401	94828401	C	T		0.559	Missense_Mutation	NM_183374.2	CYP26C1	p.Leu506Phe	MODERATE	N	N	P	P	D	PASS	6.51E-05	0	0	0.000165	0	9.02E-05	0	0	0.00136
G12*	chr10	94825892	94825892	GC	G	.	0.161	Indel	NM_183374.2	CYP26C1	p.Pro349fs	HIGH															
G12*	chr10	94828149	94828149	C	A		0.504	Missense_Mutation	NM_183374.2	CYP26C1	p.Arg422Ser	MODERATE	N	N	B	B	T	PASS	3.63E-05	0	0	0.000358	0	0	0	0	0.00091
G14*	chr19	41595958	41595958	C	T	COSM38231	0.103	Missense_Mutation	NM_000766.4	CYP2A13	p.Ala117Val	MODERATE															
G14*	chr19	15784490	15784490	T	C		0.523	Missense_Mutation	NM_023944.3	CYP4F12	p.Phe51Leu	MODERATE	N	D	D	D	D	.	.	.	.	.	.	.	.	.	.
G10*	chr8	38099779	38099779	C	T	COSM10995	0.427	Missense_Mutation	NM_001164232.1	DDHD2	p.Arg242Cys	MODERATE	D	D	D	D	D	.	.	.	.	.	.	.	.	.	.
G07*	chr16	70359480	70359480	C	G		0.375	Missense_Mutation	NM_007242.5	DDX19B	p.Leu166Val	MODERATE	D	N	P	P	T	.	.	.	.	.	.	.	.	.	.
G08	chr11	125775454	125775454	C	T		0.510	Missense_Mutation	NM_013264.4	DDX25	p.Ser46Phe	MODERATE	N	N	P	B	D	PASS	4.51E-05	0	0	0.001608	0	0	0	0	0.00091
G11	chrX	41205640	41205640	A	G		0.191	Missense_Mutation	NM_001356.4	DDX3X	p.Ser492Gly	MODERATE	D	N	B	B	D	.	.	.	.	.	.	.	.	.	.
G05*	chrX	41206251	41206251	T	TG		0.203	Indel	NM_001356.4	DDX3X	p.Arg587fs	HIGH	.	.	.	.	.	.	.	.	.	.	.	.	.	.	.
G18*	chrX	41203558	41203558	C	T	COSM29649	0.493	Nonsense_Mutation	NM_001356.4	DDX3X	p.Arg311*	HIGH	A	.	.	.	.	.	.	.	.	.	.	.	.	.	.
G07*	chr1	200613638	200613638	C	A		0.348	Missense_Mutation	NM_001031725.5	DDX59	p.Arg535Ile	MODERATE	.	.	.	.	.	.	.	.	.	.	.	.	.	.	.
G07*	chr15	66030142	66030142	G	C		0.472	Missense_Mutation	NM_001320835.1	DENND4A	p.Leu315Val	MODERATE	D	N	B	B	T	.	.	.	.	.	.	.	.	.	.
G18*	chr19	46294275	46294275	T	A	.	0.111	Missense_Mutation	NM_004943.1	DMWD	p.Asn171Ile	MODERATE															
G18*	chr3	57440556	57440556	A	C		0.170	Missense_Mutation	NM_001291661.1	DNAH12	p.Ile1134Met	MODERATE	N	N	B	B	D	.	.	.	.	.	.	.	.	.	.
G14*	chr3	57443471	57443471	C	T		0.494	Missense_Mutation	NM_001291661.1	DNAH12	p.Asp1105As	MODERATE	N	N	B	B	T	PASS	5.08E-05	0	0	0	0	0	0.000127	0	.
G14*	chr16	47001483	47001483	C	A	.	0.410	Missense_Mutation	NM_005880.3	DNAJA2	p.Gln173His	MODERATE															
G07*	chr1	65871793	65871793	G	A		0.389	Missense_Mutation	NM_001256864.1	DNAJC6	p.Arg823His	MODERATE	.	.	.	.	.	PASS	6.65E-05	0	8.64E-05	0	0	0.000106	0	0.00111	.
G07*	chr9	130982486	130982486	C	T		0.366	Nonsense_Mutation	NM_001288739.1	DNM1	p.Gln239*	HIGH	A	.	.	.	.	.	.	.	.	.	.	.	.	.	.
G03	chr2	25472564	25472564	C	T		0.464	Missense_Mutation	NM_153759.3	DNMT3A	p.Gly12Arg	MODERATE	N	N	P	B	D	.	.	.	.	.	.	.	.	.	.
G13	chr2	25523072	25523072	C	T	COSM46411	0.294	Missense_Mutation	NM_022552.4	DNMT3A	p.Arg38His	MODERATE	D	N	D	D	D	PASS	2.53E-05	9.89E-05	0.000176	0	0	0	0	0.00235	.
G08	chr2	25467132	25467132	C	T	COSM15830	0.299	Nonsense_Mutation	NM_022552.4	DNMT3A	p.Trp581*	HIGH	A	.	.	.	.	PASS	2.67E-05	0	0	0	0.00018	1.62E-05	6.31E-05	0	.
G18*	chr3	129137128	129137128	G	A		0.181	Missense_Mutation	NM_207307.2	EFCAB12	p.Thr217Ile	MODERATE	N	D	P	B	D	.	.	.	.	.	.	.	.	.	.
G04	chr10	120833042	120833042	C	T		0.470	Missense_Mutation	NM_003750.2	EIF3A	p.Met96Ile	MODERATE	N	N	B	B	D	PASS	0.0003386	0	0	0.004743	0	0	0	0	0.00182
G10*	chr10	120801536	120801536	G	A	COSM20554	0.453	Missense_Mutation	NM_003750.2	EIF3A	p.Pro1166Ser	MODERATE	D	N	P	B	T	PASS	2.47E-05	0	0	0.000347	0	0	0	0	0.00227
G10*	chr10	120801696	120801696	CCCTCGCC	C		0.284	Indel	NM_003750.2	EIF3A	p.Met1103_G	MODERATE	.	.	.	.	.	.	.	.	.	.	.	.	.	.	.
G18*	chrX	129208616	129208616	T	G	.	0.105	Missense_Mutation	NM_001127197.1	ELF4	p.Thr63Pro	MODERATE															
G08	chr22	41568668	41568668	G	A	COSM88793	0.154	Splice_Site	NM_001429.3	EP300		HIGH	D	.	.	.	.	.	.	.	.	.	.	.	.	.	.
G18*	chr22	41513487	41513487	T	G		0.124	Missense_Mutation	NM_001429.3	EP300	p.Leu131Val	MODERATE	N	N	D	P	T	.	.	.	.	.	.	.	.	.	.
G14*	chr22	41573587	41573587	CCGATGAC	C		0.546	Indel	NM_001429.3	EP300	p.Met1959fs	HIGH	.	.	.	.	.	.	.	.	.	.	.	.	.	.	.
G07*	chr18	5397301	5397301	G	A		0.284	Missense_Mutation	NM_012307.3	EPB41L3	p.Ala866Val	MODERATE	D	N	B	B	D	PASS	2.47E-05	0	0	0.000116	0	3.00E-05	0	0	0.00045
G07*	chr9	111970310	111970310	G	A		0.369	Missense_Mutation	NM_019114.4	EPB41L4B	p.Ser591Leu	MODERATE	N	N	P	B	D	PASS	2.49E-05	0.000204	0	0	0	1.50E-05	0	0	.
G14*	chr9	111956630	111956630	G	T		0.648	Missense_Mutation	NM_019114.4	EPB41L4B	p.Thr698Asn	MODERATE	N	N	B	B	D	PASS	0.0002319	0	0	0.003246	0	0	0	0	0.00409
G05*	chr19	11491837	11491837	C	A	COSM57501	0.543	Missense_Mutation	NM_000121.3	EPOR	p.Gly212Cys	MODERATE	N	D	D	P	T	PASS	0.0001229	0	0	0.003367	0	0	0	0	0.00409
G14*	chr8	72267117	72267117	G	C	.	0.241	Missense_Mutation	NM_000503.5	EYA1	p.Ser8Arg	MODERATE															
G02	chr11	82444354	82444354	G	A		0.500	Missense_Mutation	NM_175885.3	FAM181B	p.Pro140Ser	MODERATE	N	N	D	D	T	.	.	.	.	.	.	.	.	.	0.00636
G07*	chr11	82443517	82443517	C	A		0.544	Missense_Mutation	NM_175885.3	FAM181B	p.Gly419Trp	MODERATE	N	N	D	P	D	.	.	.	.	.	.	.	.	.	.
G17	chr10	90768657	90768657	G	GA		0.111	Indel	NM_000043.5	FAS	p.Ile117fs	HIGH	.	.	.	.	.	.	.	.	.	.	.	.	.	.	.
G12*	chr5	127704928	127704928	T	C		0.490	Missense_Mutation	NM_001999.3	FBN2	p.Asn732Ser	MODERATE	D	N	B	B	T	.	.	.	.	.	.	.	.	.	.
G14*	chr5	127595416	127595416	C	T		0.447	Missense_Mutation	NM_001999.3	FBN2	p.Ala2824Th	MODERATE	D	D	D	D	D	.	.	.	.	.	.	.	.	.	.
G14*	chr4	15627326	15627326	T	C		0.512	Missense_Mutation	NM_012161.3	FBXL5	p.Lys467Glu	MODERATE	N	N	B	B	T	.	.	.	.	.	.	.	.	.	.
G14*	chr17	18647637	18647637	G	A		0.608	Missense_Mutation	NM_001267585.1	FBXW10	p.Arg27Gln	MODERATE	N	N	B	B	T	PASS	8.24E-05	0	0	0.000809	0	0	0.000182	0	0.00091
G07*	chr1	27699631	27699631	C	T		0.404	Nonsense_Mutation	NM_003665.3	FCN3	p.Trp130*	HIGH	A	.	.	.	.	.	.	.	.	.	.	.	.	.	.
G07*	chr1	157660155	157660155	C	T		0.422	Missense_Mutation	NM_001320333.1	FCRL3	p.Gly527Glu	MODERATE	.	.	.	.	.	PASS	5.77E-05	0	0	0	0	0.000105	0	0	.
G07*	chr12	4479543	4479543	G	A	COSM40418	0.577	Missense_Mutation	NM_020638.2	FGF23	p.Pro241Leu	MODERATE	N	N	B	B	T	PASS	1.66E-05	0	0	0	0	3.01E-05	0	0	.
G07*	chr11	27016446	27016446	C	T		0.376	Nonsense_Mutation	NM_203371.1	FIBIN	p.Gln125*	HIGH	D	.	.	.	.	.	.	.	.	.	.	.	.	.	.
G02	chr6	159655131	159655131	T	G		0.469	Missense_Mutation	NM_032532.2	FNDC1	p.Leu1196Ar	MODERATE	N	N	P	B	D	PASS	1.92E-05	0	0	0.000248	0	0	0	0	.
G18*	chr6	159653724	159653724	C	T	COSM46515	0.511	Missense_Mutation	NM_032532.2	FNDC1	p.Ser727Phe	MODERATE	N	D	P	P	D	PASS	0.0001524	0	0	0.002103	0	0	0	0	0.00273
G12*	chr15	60297392	60297392	G	A	.	0.106	Missense_Mutation	NM_012182.2	FOXB1	p.Arg77Gln	MODERATE															
G15	chr13	41240050	41240050	C	CGCG		0.455	Indel	NM_002015.3	FOXO1	p.Ala100dup	MODERATE	.	.	.	.	.	.	.	.	.	.	.	.	.	.	.
G07*	chr1	74905192	74905192	C	G		0.322	Missense_Mutation	NM_001112808.2	FPGT-TNNI3K	p.Pro747Ala	MODERATE	.	.	.	.	.	.	.	.	.	.	.	.	.	.	.
G07*	chr1	75009593	75009593	A	C		0.397	Missense_Mutation	NM_001112808.2	FPGT-TNNI3K	p.Tyr926Ser	MODERATE	.	.	.	.	.	.	.	.	.	.	.	.	.	.	.
G12*	chrX	106808281	106808281	C	G		0.171	Missense_Mutation	NM_032428.1	FRMPD3	p.Ser460Arg	MODERATE	N	N	.	.	T	.	.	.	.	.	.	.	.	.	.
G17	chr2	186665624	186665624	A	T		0.113	Missense_Mutation	NM_173651.3	FSIP2	p.Glu3864Va	MODERATE	N	D	.	.	D	.	.	.	.	.	.	.	.	.	.
G10*	chr2	186655237	186655237	C	T		0.464	Missense_Mutation	NM_173651.3	FSIP2	p.Pro1125Leu	MODERATE	N	D	.	.	D	.	.	.	.	.	.	.	.	.	.
G12*	chr19	5844084	5844084	A	G	.	0.103	Missense_Mutation	NM_000149.3	FUT3	p.Ile256Thr	MODERATE															
G07*	chr19	5831815	5831815	A	G	.	0.131	Missense_Mutation	NM_000150.2	FUT6	p.Ile255Thr	MODERATE															
G05*	chr11	86665923	86665923	G	A		0.423	Missense_Mutation	NM_012193.3	FZD4	p.His69Tyr	MODERATE	A	D	B	B	D	PASS	0.0005621	0	0	0.004267	0.00034	0.000372	0.000194	0	0.00864
G07*	chr11	86662214	86662214	C	A		0.231	Missense_Mutation	NM_012193.3	FZD4	p.Lys528Asn	MODERATE	D	N	D	D	T	.	.	.	.	.	.	.	.	.	.
G12*	chrX	153906478	153906478	C	T	.	0.102	Missense_Mutation	NM_001081573.2	GAB3	p.Asp581Asn	MODERATE															
G03	chr3	50294174	50294174	C	G		0.397	Missense_Mutation	NM_002070.3	GNAI2	p.Gln205Glu	MODERATE	D	D	D	D	D	.	.	.	.	.	.	.	.	.	.
G07*	chr3	154055541	154055541	G	C		0.597	Missense_Mutation	NM_001038705.1	GPR149	p.Gln715Glu	MODERATE	D	N	D	D	D	.	.	.	.	.	.	.	.	.	.
G10*	chr3	154146993	154146993	C	A		0.462	Missense_Mutation	NM_001038705.1	GPR149	p.Val138Leu	MODERATE	N	N	B	B	T	.	.	.	.	.	.	.	.	.	.
G07*	chr6	117130645	117130645	C	A		0.675	Missense_Mutation	NM_148963.3	GPRC6A	p.Met110Ile	MODERATE	N	N	B	B	T	.	.	.	.	.	.	.	.	.	.

G18*	chrX	122551326	122551326	T	C		0.416	Missense_Mutation	NM_000828.4	GRIA3	p.Phe525Ser	MODERATE	D	D	D	D	D	.	.	.	.	.	.	.	.	.	.
G12*	chr13	113979972	113979972	A	ACT	.	0.123	Indel	NM_001286732.1	GRTP1	p.Cys309fs	HIGH															
G07*	chr7	76941192	76941192	AATGAAGT	A	.	0.145	Indel	NM_017439.3	GSAP	p.Glu805_Ile8	MODERATE															
G12*	chr4	156651343	156651343	G	A	COSM41230	0.450	Missense_Mutation	NM_000856.5	GUCY1A3	p.Gly678Asp	MODERATE	D	N	P	B	T	PASS	8.28E-06	0	0	0.000116	0	0	0	0	.
G07*	chr4	156618029	156618029	A	C		0.386	Missense_Mutation	NM_000856.5	GUCY1A3	p.Thr4Pro	MODERATE	D	N	B	B	T	.	.	.	.	.	.	.	.	.	.
G14*	chr7	43484794	43484794	G	A	COSM38065	0.466	Missense_Mutation	NM_015052.4	HECW1	p.Ala675Thr	MODERATE	N	N	B	B	T	PASS	1.72E-05	0	8.77E-05	0	0	0	6.13E-05	0.00117	.
G18*	chr6	32548544	32548544	T	G	COSM13191	0.137	Missense_Mutation	NM_002124.3.3	HLA-DRB1	p.Ile248Leu	MODERATE															
G07*	chr19	8550541	8550541	G	A		0.388	Missense_Mutation	NM_005968.4	HNRNPM	p.Arg410His	MODERATE	D	N	D	P	D	.	.	.	.	.	.	.	.	.	.
G14*	chr7	27213523	27213523	G	T	COSM56568	0.400	Missense_Mutation	NM_018951.3	HOXA10	p.Pro135Thr	MODERATE															
G18*	chr7	27186929	27186929	G	A		0.171	Splice_Site	NM_024014.3	HOXA6	p.Ala147Val	MODERATE	D	N	P	B	D	.	.	.	.	.	.	.	.	.	.
G07*	chr3	186389555	186389555	C	T	COSM36959	0.692	Nonsense_Mutation	NM_000412.4	HRG	p.Arg179*	HIGH	A	.	.	.	.	PASS	0.0001236	0.000865	0.000519	0	0	0	0	0.00662	.
G14*	chr1	161495814	161495814	A	G	.	0.237	Missense_Mutation	NM_002155.4	HSPA6	p.Asn456Asp	MODERATE															
G18*	chr19	36246569	36246569	C	T		0.294	Missense_Mutation	NM_144617.2	HSPB6	p.Val113Ile	MODERATE	D	N	B	B	T	.	.	.	.	.	.	.	.	.	.
G10*	chr8	39840207	39840207	G	C		0.431	Missense_Mutation	NM_194294.2	IDO2	p.Val131Leu	MODERATE	D	N	B	B	T	.	.	.	.	.	.	.	.	.	.
G16	chr3	151154921	151154921	C	CCTGT		0.469	Indel	NM_178822.4	IGSF10	p.Pro2477fs	HIGH	.	.	.	.	.	.	.	.	.	.	.	.	.	.	.
G02	chr3	151163162	151163162	A	G		0.491	Missense_Mutation	NM_178822.4	IGSF10	p.Ile1536Thr	MODERATE	N	N	B	B	T	PASS	0.0016061	9.61E-05	0	0.002196	0.01497	0.001064	0.000121	0	0.00591
G17	chr3	151164199	151164199	C	A		0.496	Missense_Mutation	NM_178822.4	IGSF10	p.Met1190Ile	MODERATE	N	N	B	B	T	PASS	8.24E-06	0	0	0.000116	0	0	0	0	.
G02	chr3	151164933	151164933	CTAA	C		0.508	Indel	NM_178822.4	IGSF10	p.Leu945del	MODERATE	.	.	.	.	.	PASS	8.24E-05	0	0	0.001156	0	0	0	0	.
G07*	chr3	151163622	151163622	C	A		0.683	Nonsense_Mutation	NM_178822.4	IGSF10	p.Glu1383*	HIGH	A	.	.	.	.	.	.	.	.	.	.	.	.	.	.
G14*	chr1	159899591	159899591	G	C		0.584	Nonsense_Mutation	NM_001135050.1	IGSF9	p.Tyr721*	HIGH	.	.	.	.	.	.	.	.	.	.	.	.	.	.	.
G07*	chr8	42147720	42147720	A	G		0.823	Missense_Mutation	NM_001556.2	IKBKB	p.Met83Val	MODERATE	D	N	P	B	D	PASS	0.0001987	0	0	0.002722	0	0	0	0	0.00136
G01	chr8	95877770	95877770	C	A		0.507	Missense_Mutation	NM_017864.3	INTS8	p.Pro705Thr	MODERATE	D	.	.	.	.	PASS	5.77E-05	0	0	0.000809	0	0	0	0	0.00182
G14*	chr3	52817343	52817343	G	A	.	0.426	Missense_Mutation	NM_002215.3	ITIH1	p.Asp405Asn	MODERATE															
G16	chr1	65304237	65304237	G	A	COSM49905	0.313	Missense_Mutation	NM_001320923.1	JAK1	p.Pro960Ser	MODERATE	.	.	.	.	.	.	.	.	.	.	.	.	.	.	.
G03	chr1	65310517	65310517	C	T	COSM41751	0.391	Missense_Mutation	NM_001320923.1	JAK1	p.Arg724His	MODERATE	.	.	.	.	.	.	.	.	.	.	.	.	.	.	.
G16	chr1	65311225	65311225	T	G		0.103	Missense_Mutation	NM_001320923.1	JAK1	p.Lys696Gln	MODERATE	.	.	.	.	.	.	.	.	.	.	.	.	.	.	.
G16	chr1	65316521	65316521	C	A		0.239	Missense_Mutation	NM_001320923.1	JAK1	p.Ser574Ile	MODERATE	.	.	.	.	.	.	.	.	.	.	.	.	.	.	.
G10*	chr1	65311203	65311203	C	A	COSM30594	0.441	Missense_Mutation	NM_001320923.1	JAK1	p.Ser703Ile	MODERATE	.	.	.	.	.	.	.	.	.	.	.	.	.	.	.
G03	chr19	17945918	17945918	A	G	COSM32731	0.703	Missense_Mutation	NM_000215.3	JAK3	p.Val674Ala	MODERATE	D	D	D	D	D	.	.	.	.	.	.	.	.	.	.
G13	chr19	17945918	17945918	A	G	COSM32731	0.981	Missense_Mutation	NM_000215.3	JAK3	p.Val674Ala	MODERATE	D	D	D	D	D	.	.	.	.	.	.	.	.	.	.
G04	chr19	17945969	17945969	C	T	COSM11481	0.423	Missense_Mutation	NM_000215.3	JAK3	p.Arg657Gln	MODERATE	D	D	D	D	D	.	.	.	.	.	.	.	.	.	.
G09	chr19	17948006	17948006	G	A	COSM34215	0.755	Missense_Mutation	NM_000215.3	JAK3	p.Ala573Val	MODERATE	D	D	D	D	D	.	.	.	.	.	.	.	.	.	.
G16	chr19	17948006	17948006	G	A	COSM34215	0.537	Missense_Mutation	NM_000215.3	JAK3	p.Ala573Val	MODERATE	D	D	D	D	D	.	.	.	.	.	.	.	.	.	.
G18*	chr19	8389400	8389400	T	G		0.152	Missense_Mutation	NM_198471.2	KANK3	p.Glu772Ala	MODERATE	D	D	D	D	D	.	.	.	.	.	.	.	.	.	.
G08	chr10	76781848	76781848	C	G		0.119	Missense_Mutation	NM_012330.3	KAT6B	p.Asp1077Gl	MODERATE	N	N	B	B	T	.	.	.	.	.	.	.	.	.	.
G07*	chr10	76732361	76732361	T	C		0.778	Missense_Mutation	NM_012330.3	KAT6B	p.Ile342Thr	MODERATE	D	D	D	D	D	PASS	0.0002389	0	8.64E-05	0.002196	0	6.00E-05	0.000303	0.0011	0.005
G16	chr8	73848533	73848533	G	T		0.473	Missense_Mutation	NM_004770.2	KCNB2	p.Ala315Ser	MODERATE	D	D	D	D	D	.	.	.	.	.	.	.	.	.	.
G05*	chr8	73849869	73849869	C	A		0.460	Missense_Mutation	NM_004770.2	KCNB2	p.Thr760Asn	MODERATE	N	N	B	B	T	PASS	0.0001491	0	0	0.001972	0	0	0	0	0.00545
G18*	chr8	73849869	73849869	C	A		0.543	Missense_Mutation	NM_004770.2	KCNB2	p.Thr760Asn	MODERATE	N	N	B	B	T	PASS	0.0001491	0	0	0.001972	0	0	0	0	0.00545
G14*	chr8	73848509	73848509	C	T	COSM11015	0.577	Missense_Mutation	NM_004770.2	KCNB2	p.Arg307Cys	MODERATE	D	D	D	D	D	.	.	.	.	.	.	.	.	.	.
G01	chr3	19492810	19492810	G	A	COSM33343	0.186	Missense_Mutation	NM_144633.2	KCNH8	p.Arg580His	MODERATE	D	D	P	P	T	PASS	4.95E-05	0	0	0.000694	0	0	0	0	.
G11	chr3	19575088	19575088	G	A	COSM29471	0.445	Missense_Mutation	NM_144633.2	KCNH8	p.Gly941Arg	MODERATE	N	N	P	B	T	PASS	0.0001401	9.61E-05	0	0.000464	0	3.00E-05	0.000606	0	0.00091
G14*	chr17	21319092	21319092	C	A		0.174	Nonsense_Mutation	NM_021012.4	KCNJ12	p.Tyr146*	HIGH	D	.	.	.	.	PASS	0.0001407	0	0	0.001852	0	1.51E-05	0	0	.
G05*	chr17	21319728	21319728	T	A	.	0.125	Missense_Mutation	NM_021012.4	KCNJ12	p.Ser358Arg	MODERATE															
G11	chr20	36851951	36851951	C	A		0.495	Missense_Mutation	NM_001029864.1	KIAA1755	p.Gly753Trp	MODERATE	N	D	D	D	D	PASS	1.78E-05	0	0	0.000119	0	1.60E-05	0	0	0.00045
G15	chr20	36856566	36856566	C	T	COSM49101	0.100	Missense_Mutation	NM_001029864.1	KIAA1755	p.Ala650Thr	MODERATE	D	D	D	D	D	.	.	.	.	.	.	.	.	.	.
G14*	chr20	36869755	36869755	C	G	.	0.159	Missense_Mutation	NM_001029864.1	KIAA1755	p.Ala260Pro	MODERATE															
G16	chr15	90190925	90190925	G	A		0.507	Nonsense_Mutation	NM_198525.2	KIF7	p.Gln488*	HIGH	A	.	.	.	.	.	.	.	.	.	.	.	.	.	.
G15	chr19	55351095	55351095	T	A		0.541	Missense_Mutation	NM_012314.5	KIR2DS4	p.Ser195Thr	MODERATE	.	.	.	.	.	.	.	.	.	.	.	.	.	.	.
G14*	chr1	158063228	158063228	G	A	COSM33998	0.672	Missense_Mutation	NM_018240.6	KIRREL	p.Arg524His	MODERATE	.	.	.	.	.	PASS	4.12E-05	9.61E-05	0	0.000116	0	4.50E-05	0	0	.
G17	chr11	118307445	118307445	G	A		0.473	Missense_Mutation	NM_001197104.1	KMT2A	p.Gly73Glu	MODERATE	D	N	B	B	D	PASS	0.0005271	0	0	0.007629	0	0	0	0	0.00409
G11	chr11	118353185	118353185	C	A		0.400	Missense_Mutation	NM_001197104.1	KMT2A	p.Pro1354His	MODERATE	D	N	D	P	D	.	.	.	.	.	.	.	.	.	0.00091
G03	chr19	36218411	36218411	C	T	COSM39709	0.462	Missense_Mutation	NM_014727.2	KMT2B	p.Ala1397Va	MODERATE	N	N	B	B	T	PASS	3.41E-05	0.000108	0	0.000236	0	0	6.10E-05	0	0.00091
G03	chr12	25398281	25398281	C	T	COSM11401	0.262	Missense_Mutation	NM_033360.3	KRAS	p.Gly13Asp	MODERATE	D	D	B	B	D	.	.	.	.	.	.	.	.	.	.
G02	chr12	25398285	25398285	C	A	COSM11401	0.402	Missense_Mutation	NM_033360.3	KRAS	p.Gly12Cys	MODERATE	D	D	D	D	D	PASS	1.98E-05	0	0	0.000126	0	1.87E-05	0	0	.
G10*	chr12	25398284	25398284	C	T	COSM11353	0.427	Missense_Mutation	NM_033360.3	KRAS	p.Gly12Asp	MODERATE	D	D	B	B	D	PASS	1.98E-05	0.000111	0	0	0	1.87E-05	0	0	.
G14*	chr12	25378649	25378649	T	G	.	0.375	Missense_Mutation	NM_033360.3	KRAS	p.Lys117Gln	MODERATE															
G07*	chr17	39394482	39394482	T	C	COSM15210	0.104	Missense_Mutation	NM_031963.2	KRTAP9-8	p.Ile60Thr	MODERATE															
G13	chr9	133911635	133911635	G	A		0.439	Missense_Mutation	NM_006059.3	LAMC3	p.Gly298Ser	MODERATE	D	D	D	D	D	PASS	9.41E-05	0.000107	0	0.000354	0	0.00011	0	0	0.00045
G08	chr9	133914377	133914377	A	G		0.552	Missense_Mutation	NM_006059.3	LAMC3	p.Gln368Arg	MODERATE	N	N	B	B	T	PASS	0.0001491	0	0	0.002087	0	0	0	0	0.00273
G13	chr9	133948215	133948215	C	T	COSM11060	0.520	Missense_Mutation	NM_006059.3	LAMC3	p.Ala1137Va	MODERATE	N	N	P	B	T	PASS	1.14E-05	0	0	0.000149	0	0	0	0	.
G10*	chr1	180235528	180235528	C	T		0.406	Splice_Site	NM_033343.3	LHX4	p.Arg84Cys	MODERATE	.	.	.	.	.	PASS	9.08E-05	0	0	0.000232	0	0.00012	6.06E-05	0	0.00045
G14*	chr5	38486079	38486079	C	T		0.492	Missense_Mutation	NM_001127671.1	LIFR	p.Arg780His	MODERATE	N	N	B	B	T	.	.	.	.	.	.	.	.	.	.
G13	chr3	8578876	8578876	T	C		0.534	Missense_Mutation	NM_014583.3	LMCD1	p.Ile46Thr	MODERATE	D	D	P	B	T	PASS	0.000191	0	0	0.002551	0	1.51E-05	0	0	0.00864
G07*	chr6	44185284	44185284	C	T	.	0.372	Missense_Mutation	NM_001315494.1	LOC10192972	p.Arg26Cys	MODERATE															
G18*	chr15	101606320	101606320	A	C		0.137	Missense_Mutation	NM_024652.4	LRRK1	p.His1893Pro	MODERATE	N	N	B	B	T	.	.	.	.	.	.	.	.	.	.
G14*	chr15	101549225	101549225	G	C		0.521	Missense_Mutation	NM_024652.4	LRRK1	p.Glu316Gln	MODERATE	D	N	P	B	T	PASS	4.98E-05	0	0	0.000696	0	0	0	0	0.00045
G07*	chr10	102763751	102763751	G	A		0.608	Missense_Mutation	NM_001318099.1	LZTS2	p.Arg299Gln	MODERATE	N	N	B	B	T	PASS	8.51E-06	0	0	0	0	1.56E-05	0	0	.
G05*	chr3	65365010	65365010	C	T		0.482	Missense_Mutation	NM_001033057.1	MAGI1	p.Arg974Gln	MODERATE	D	.	.	.	.	PASS	0.0038639	0.000109	0	0.002823	0.02478	0.003669	0.001095	0	0.00773
G14*	chr3	65365231	65365231	T	A		0.531	Splice_Site	NM_001033057.1	MAGI1		HIGH	D	.	.	.	.	.	.	.	.	.	.	.	.	.	.
G01	chr7	77649102	77649102	G	T		0.326	Missense_Mutation	NM_012301.3	MAGI2	p.Leu1300Ile	MODERATE	D	N	P	P	T	PASS	4.17E-05	0	0	0.000582	0	0	0	0	0.00136
G07*	chr7	77708272	77708272	G	A		0.273	Missense_Mutation	NM_012301.3	MAGI2	p.Pro1233Leu	MODERATE	D	D	D	D	D	.	.	.	.	.	.	.	.	.	.
G14*	chr6	137018474	137018474	T	A		0.426	Missense_Mutation	NM_005923.3	MAP3K5	p.Leu286Phe	MODERATE	D	N	P	P	D	.	.	.	.	.	.	.	.	.	.
G07*	chr6	136742901	136742901	G	A		0.426	Missense_Mutation	NM_001198609.1	MAP7	p.Ala57Val	MODERATE	D	N	B	B	T	.	.	.	.	.	.	.	.	.	.
G05*	chr1	3421997	3421997	C	T		0.505	Missense_Mutation	NM_001409.3	MEGF6	p.Arg681His	MODERATE	N	N	B	B	T	PASS	9.79E-06	0	0	0	0	1.77E-05	0	0	.
G07*	chr1	3432038	3432038	G	C		0.291	Missense_Mutation	NM_001409.3	MEGF6	p.Leu220Val	MODERATE	N	N	B	B	T	.	.	.	.	.	.	.	.	.	.
G02	chr15	41962104	41962104	A	G		0.659	Missense_Mutation	NM_001164273.1	MGA	p.Thr338Ala	MODERATE	N	N	B	B	T	PASS	0.0005094	0	0	0.006193	0	0	0.000488	0	0.00773
G09	chr15	42035256	42035256	G	A		0.153	Missense_Mutation	NM_001164273.1	MGA	p.Val1700Me	MODERATE	D	N	B	B	T	PASS	4.98E-05	0.000102	0	0.000464	0	0	6.08E-05	0	0.00136
G04	chr15	42042313	42042313	G	A		0.702	Missense_Mutation	NM_001164273.1	MGA	p.Asp2170As	MODERATE	N	N	B	B	T	.	.	.	.	.	.	.	.	.	.
G05*	chr7	141755395	141755395	A	G		0.555	Missense_Mutation	NM_004668.2	MGAM	p.Met1118Va	MODERATE	N	D	B	B	T	PASS	2.48E-05	0	0	0.000348	0	0	0	0	0.00136
G02	chr1	170928613	170928613	C	T	COSM34782	0.640	Missense_Mutation	NM_001163629.1	MROH9	p.Pro55Ser	MODERATE	.	.	.	.	.	.	.	.	.	.	.	.	.	.	.
G07*	chr1	170967438	170967438	A	T		0.311	Missense_Mutation	NM_025063.2	MROH9	p.Gln540Leu	MODERATE	.	.	.	.	.	.	.	.	.	.	.	.	.	.	.
G07*	chr22	19423399	19423399	A	G		0.252	Missense_Mutation	NM_003776.3	MRPL40	p.Thr179Ala	MODERATE	D	D	D	D	D	.	.	.	.	.	.	.	.	.	.
G10*	chr1	155161042	155161042	G	A	.	0.103	Missense_Mutation	NM_001204286.1	MUC1	p.Pro171Leu	MODERATE															

G18*	chr7	100549540	100549540	A	T	.	0.111	Missense_Mutation	NM_005960.1	MUC3A	p.Ser41Cys	MODERATE															
G12*	chr7	100550478	100550478	C	A	.	0.164	Nonsense_Mutation	NM_005960.1	MUC3A	p.Tyr353*	HIGH															
G14*	chr7	100551346	100551346	C	G	.	0.613	Missense_Mutation	NM_005960.1	MUC3A	p.Leu643Val	MODERATE															
G12*	chr3	195510011	195510011	C	T	COSM3759	0.118	Missense_Mutation	NM_018406.6	MUC4	p.Ala2814Th	MODERATE															
G18*	chrX	105450251	105450251	T	C	.	0.107	Missense_Mutation	NM_001171020.1	MUM1L1	p.Ser276Pro	MODERATE															
G06	chr16	15818834	15818834	C	T		0.403	Missense_Mutation	NM_001040114.1	MYH11	p.Val1299Ile	MODERATE	D	N	B	B	T	PASS	3.35E-05	9.91E-05	0	0.000233	0	1.52E-05	0	0	0.00045
G08	chr17	10367997	10367997	C	G		0.631	Splice_Site	NM_017533.2	MYH4		HIGH	D	.	.	.	.	PASS	1.65E-05	0	0	0.000232	0	0	0	0	0.00091
G14*	chr17	10369718	10369718	C	T		0.482	Missense_Mutation	NM_017533.2	MYH4	p.Glu74Lys	MODERATE	D	D	B	B	D	.	.	.	.	.	.	.	.	.	.
G08	chr22	26219615	26219615	T	C		0.429	Missense_Mutation	NM_001318245.1	MYO18B	p.Trp889Arg	MODERATE	N	N	B	B	T	PASS	8.28E-06	0	0	0	0	1.50E-05	0	0	.
G08	chr22	26231363	26231363	T	C		0.239	Missense_Mutation	NM_001318245.1	MYO18B	p.Leu1055Pro	MODERATE	D	D	D	D	D	PASS	8.28E-05	0	0	0.001159	0	0	0	0	0.00409
G08	chr22	26291140	26291140	C	T		0.384	Missense_Mutation	NM_001318245.1	MYO18B	p.Arg1522Cy	MODERATE	D	D	D	D	D	PASS	9.93E-06	0	0	0	0	1.77E-05	0	0	.
G04	chr22	26422880	26422880	G	A	COSM5742	0.515	Missense_Mutation	NM_001318245.1	MYO18B	p.Glu2315Ly	MODERATE	N	N	B	B	T	PASS	8.58E-06	0	0	0.000117	0	0	0	0	0.00182
G09	chr4	140283025	140283025	G	A		0.433	Missense_Mutation	NM_057175.3	NAA15	p.Glu563Lys	MODERATE	D	N	B	B	T	PASS	8.29E-06	0	0	0.000116	0	0	0	0	0.00182
G14*	chr4	140258100	140258100	CAT	C		0.423	Indel	NM_057175.3	NAA15	p.His80fs	HIGH	.	.	.	.	.	PASS	8.30E-06	0	0	0	0	0	6.09E-05	0	.
G18*	chr1	16891365	16891365	G	A	.	0.153	Missense_Mutation	NM_017940.4	NBPF1	p.Thr1039Me	MODERATE															
G07*	chr1	16891340	16891340	T	A	.	0.119	Missense_Mutation	NM_017940.4	NBPF1	p.Arg1047Se	MODERATE															
G09	chr11	134023210	134023210	G	A		0.828	Missense_Mutation	NM_015261.2	NCAPD3	p.Thr1434Ile	MODERATE	N	N	B	B	T	PASS	0.0001238	0	0	0.001504	0	1.50E-05	6.06E-05	0	0.00455
G09	chr11	134027913	134027913	C	T		0.920	Missense_Mutation	NM_015261.2	NCAPD3	p.Val1362Me	MODERATE	N	N	D	P	D	PASS	0.0001566	0	0.000346	0.001502	0	1.50E-05	6.07E-05	0.00441	0.00455
G07*	chr11	134026927	134026927	A	G	.	0.106	Missense_Mutation	NM_015261.2	NCAPD3	p.Ile1413Thr	MODERATE															
G14*	chr2	133542220	133542220	G	C	.	0.442	Missense_Mutation	NM_207363.2	NCKAP5	p.Pro722Ala	MODERATE															
G11	chr8	71060537	71060537	T	G		0.480	Missense_Mutation	NM_001321703.1	NCOA2	p.Gln859Pro	MODERATE	D	N	B	B	T	.	.	.	.	.	.	.	.	.	.
G07*	chr8	71071750	71071750	T	C		0.683	Missense_Mutation	NM_001321703.1	NCOA2	p.Met372Val	MODERATE	N	N	B	B	T	.	.	.	.	.	.	.	.	.	.
G16	chr17	29562732	29562732	T	C		0.434	Missense_Mutation	NM_001042492.2	NF1	p.Met1271Th	MODERATE	D	D	D	P	D	.	.	.	.	.	.	.	.	.	.
G07*	chr12	6670183	6670183	G	A		0.224	Missense_Mutation	NM_001258309.1	NOP2	p.Arg454Trp	MODERATE	D	D	D	D	D	.	.	.	.	.	.	.	.	.	.
G08	chr9	139391634	139391634	C	A		0.393	Missense_Mutation	NM_017617.4	NOTCH1	p.Gly2186Va	MODERATE	D	D	D	D	T	PASS	7.59E-05	0	0	0.001053	0	0	0	0	0.00227
G04	chr9	139407526	139407526	G	A		0.377	Missense_Mutation	NM_017617.4	NOTCH1	p.Thr805Met	MODERATE	D	D	D	D	D	PASS	2.52E-05	0	0	0	0	3.05E-05	6.07E-05	0	0.00182
G15	chr7	98254392	98254392	T	A		0.520	Missense_Mutation	NM_002523.2	NPTX2	p.Phe268Ile	MODERATE	D	D	D	D	D	PASS	4.97E-05	0	0	0.000698	0	0	0	0	0.00182
G07*	chr7	98247083	98247083	G	A		0.314	Missense_Mutation	NM_002523.2	NPTX2	p.Gly104Ser	MODERATE	D	N	B	B	T	.	.	.	.	.	.	.	.	.	.
G04	chr1	115258745	115258745	C	A	COSM570	0.434	Missense_Mutation	NM_002524.4	NRAS	p.Gly13Cys	MODERATE	.	.	.	.	.	.	.	.	.	.	.	.	.	.	.
G09	chr1	115258748	115258748	C	T	COSM563	0.500	Missense_Mutation	NM_002524.4	NRAS	p.Gly12Ser	MODERATE	.	.	.	.	.	.	.	.	.	.	.	.	.	.	.
G07*	chr7	107872929	107872929	C	T	COSM55431	0.282	Missense_Mutation	NM_001037132.2	NRCAM	p.Asp90Asn	MODERATE	D	N	D	P	T	PASS	8.30E-06	0	0	0	0	1.51E-05	0	0	.
G04	chrX	105167141	105167141	C	T	COSM5411	0.887	Missense_Mutation	NM_198465.3	NRK	p.Ser881Leu	MODERATE	N	D	D	D	D	.	.	.	.	.	.	.	.	.	.
G14*	chrX	105153070	105153070	G	A	.	0.414	Missense_Mutation	NM_198465.3	NRK	p.Met479Ile	MODERATE															
G14*	chr11	64434829	64434829	T	C		0.429	Missense_Mutation	NM_015080.3	NRXN2	p.Tyr564Cys	MODERATE	D	D	D	D	D	PASS	0.0001237	0	0	0	0	0.000225	0	0	.
G04	chr5	176722221	176722221	G	A		0.503	Missense_Mutation	NM_022455.4	NSD1	p.Val2618Ile	MODERATE	N	N	B	B	T	PASS	0.0001237	0	8.64E-05	0.001272	0	1.50E-05	0.000121	0.0011	0.005
G11	chr15	88576178	88576178	C	T	COSM2014	0.511	Missense_Mutation	NM_001012338.2	NTRK3	p.Asp499Asn	MODERATE	D	N	D	B	D	PASS	7.61E-05	0	0	0.000352	0	7.65E-05	0	0	0.00136
G06	chr4	37435573	37435573	G	A		0.440	Missense_Mutation	NM_001144990.1	NWD2	p.Ala213Thr	MODERATE	N	N	B	B	T	.	.	.	.	.	.	.	.	.	0.00045
G07*	chr4	37448817	37448817	A	T		0.497	Missense_Mutation	NM_001144990.1	NWD2	p.His1736Leu	MODERATE	D	N	B	B	D	.	.	.	.	.	.	.	.	.	.
G07*	chrX	41333163	41333163	C	T		0.828	Missense_Mutation	NM_022567.2	NYX	p.Arg153Cys	MODERATE	D	D	D	D	T	PASS	6.31E-05	0	0	0	0	0	0.000278	0	.
G14*	chr11	6790066	6790066	G	T		0.534	Missense_Mutation	NM_001004490.1	OR2AG2	p.Ser41Arg	MODERATE	D	N	P	P	D	.	.	.	.	.	.	.	.	.	.
G10*	chr11	56185444	56185444	G	A	COSM2510	0.369	Missense_Mutation	NM_001004744.1	OR5R1	p.Arg89Cys	MODERATE	N	D	P	P	D	PASS	4.13E-05	0	0	0	0	1.50E-05	0.000242	0	.
G04	chr11	124135688	124135688	C	T		0.130	Splice_Site	NM_001002905.1	OR8G1		HIGH	.	.	.	.	.	.	.	.	.	.	.	.	.	.	.
G04	chr11	124135725	124135725	G	T		0.113	Missense_Mutation	NM_001002905.1	OR8G1	p.Ala300Ser	MODERATE	.	.	.	.	.	.	.	.	.	.	.	.	.	.	.
G06	chr11	124135725	124135725	G	T		0.109	Missense_Mutation	NM_001002905.1	OR8G1	p.Ala300Ser	MODERATE	.	.	.	.	.	.	.	.	.	.	.	.	.	.	.
G07*	chr11	124120778	124120778	C	T		0.554	Missense_Mutation	NM_001002905.1	OR8G1	p.Ala119Val	MODERATE	.	.	.	.	.	PASS	8.28E-06	0	0	0	0	0	6.06E-05	0	.
G03	chr8	101719224	101719224	T	TGGATGAGGTCTGGCA		0.102	Indel	NM_002568.3	PABPC1	p.Phe447fs	HIGH	.	.	.	.	.	.	.	.	.	.	.	.	.	.	.
G07*	chrX	110388132	110388132	T	G		0.795	Missense_Mutation	NM_001128168.2	PAK3	p.Ser107Arg	MODERATE	D	N	P	P	D	.	.	.	.	.	.	.	.	.	.
G07*	chr10	34688325	34688325	C	T		0.484	Missense_Mutation	NM_019619.3	PARD3	p.Val275Ile	MODERATE	D	N	D	D	T	.	.	.	.	.	.	.	.	.	.
G14*	chr5	140432370	140432370	G	A		0.383	Missense_Mutation	NM_013340.3	PCDHB1	p.Glu439Lys	MODERATE	N	N	B	B	T	.	.	.	.	.	.	.	.	.	.
G12*	chr5	140782658	140782658	A	G	.	0.111	Missense_Mutation	NM_018921.2	PCDHGA9	p.Asn47Asp	MODERATE															
G14*	chr11	45935890	45935890	T	TA		0.553	Indel	NM_057174.2	PEX16	p.Tyr224fs	HIGH	.	.	.	.	.	.	.	.	.	.	.	.	.	.	.
G14*	chr17	8159874	8159874	G	A		0.489	Missense_Mutation	NM_012393.2	PFAS	p.Arg285Gln	MODERATE	N	N	B	B	T	PASS	8.25E-05	0	0	0.00081	0	4.50E-05	0	0	0.00091
G02	chr18	44392461	44392461	T	C		0.289	Missense_Mutation	NM_004671.4	PIAS2	p.Arg601Gly	MODERATE	D	N	D	D	T	.	.	.	.	.	.	.	.	.	.
G07*	chr7	106509640	106509640	C	T		0.230	Missense_Mutation	NM_001282426.1	PIK3CG	p.Ala545Val	MODERATE	D	N	B	B	T	.	.	.	.	.	.	.	.	.	.
G05*	chr5	67576532	67576532	A	G		0.524	Missense_Mutation	NM_181523.2	PIK3R1	p.Met271Val	MODERATE	N	N	B	B	T	PASS	8.25E-06	9.71E-05	0	0	0	0	0	0	0.00045
G15	chr6	37139063	37139063	G	A	COSM1161	0.488	Missense_Mutation	NM_001243186.1	PIM1	p.Glu226Lys	MODERATE	D	D	B	B	D	PASS	0.0001825	0	0	0.002438	0	1.51E-05	0	0	0.00045
G07*	chr4	88987032	88987032	G	A		0.648	Splice_Site	NM_000297.3	PKD2		HIGH	D	.	.	.	.	.	.	.	.	.	.	.	.	.	.
G08	chr1	204217975	204217975	G	A	COSM2122	0.540	Missense_Mutation	NM_014935.4	PLEKHA6	p.Arg600Cys	MODERATE	.	.	.	.	.	PASS	1.65E-05	0	0	0.000116	0	1.50E-05	0	0	0.00045
G07*	chr1	204230532	204230532	G	C		0.409	Missense_Mutation	NM_014935.4	PLEKHA6	p.Ser142Arg	MODERATE	.	.	.	.	.	.	.	.	.	.	.	.	.	.	.
G14*	chrX	84634306	84634306	C	A		0.471	Missense_Mutation	NM_001307940.1	POF1B	p.Val52Leu	MODERATE	D	N	D	D	D	.	.	.	.	.	.	.	.	.	.
G05*	chr4	147560457	147560457	T	TGGTGGCC	.	0.316	Indel	NM_004575.2	POU4F2	p.Gly56_Gly5	MODERATE															
G07*	chr7	94791274	94791274	G	C		0.200	Missense_Mutation	NM_001166160.1	PPP1R9A	p.Gly582Ala	MODERATE	D	D	P	P	D	.	.	.	.	.	.	.	.	.	.
G14*	chr11	64694270	64694270	G	A		0.374	Missense_Mutation	NM_006244.3	PPP2R5B	p.Asp96Asn	MODERATE	D	D	D	P	D	.	.	.	.	.	.	.	.	.	.
G02	chr6	106536122	106536122	C	G		0.451	Missense_Mutation	NM_001198.3	PRDM1	p.Ala30Gly	MODERATE	D	N	P	B	T	.	.	.	.	.	.	.	.	.	0.00136
G08	chr6	106536206	106536206	G	GT		0.411	Indel	NM_001198.3	PRDM1	p.Thr59fs	HIGH	.	.	.	.	.	.	.	.	.	.	.	.	.	.	.
G18*	chr6	106554373	106554373	A	G		0.571	Splice_Site	NM_001198.3	PRDM1	p.Gln634Arg	MODERATE	D	N	B	B	D	PASS	0.0001239	0	0	0.001625	0	1.50E-05	0	0	0.00455
G05*	chr20	47265913	47265913	T	G		0.551	Missense_Mutation	NM_020820.3	PREX1	p.Asp1077Al	MODERATE	D	D	D	P	D	PASS	8.24E-06	0	0	0.000116	0	0	0	0	.
G18*	chr20	47273663	47273663	G	C		0.369	Missense_Mutation	NM_020820.3	PREX1	p.Pro680Ala	MODERATE	D	D	P	B	D	.	.	.	.	.	.	.	.	.	.
G05*	chr1	110998939	110998939	G	A		0.424	Missense_Mutation	NM_032414.2	PROK1	p.Arg95His	MODERATE	.	.	.	.	.	PASS	4.12E-05	9.61E-05	0	0	0.0003	3.00E-05	0	0	.
G18*	chr1	110998864	110998864	T	G		0.176	Missense_Mutation	NM_032414.2	PROK1	p.Phe70Cys	MODERATE	.	.	.	.	.	.	.	.	.	.	.	.	.	.	.
G07*	chr1	170695392	170695392	G	A		0.340	Missense_Mutation	NM_022716.3	PRRX1	p.Arg150His	MODERATE	.	.	.	.	.	.	.	.	.	.	.	.	.	.	.
G13	chr9	98278984	98278984	C	CCTCCTCCTCCTCCT		0.111	Indel	NM_001083603.1	PTCH1	p.Gly40fs	HIGH	.	.	.	.	.	.	.	.	.	.	.	.	.	.	.
G10*	chr9	98239912	98239912	C	T	COSM1111	0.520	Missense_Mutation	NM_000264.3	PTCH1	p.Val474Ile	MODERATE	D	N	D	D	D	PASS	8.27E-06	9.71E-05	0	0	0	0	0	0	0.00045
G15	chr19	14584294	14584294	C	T		0.135	Missense_Mutation	NM_000955.2	PTGER1	p.Arg280Gln	MODERATE	N	N	P	B	T	.	.	.	.	.	.	.	.	.	.
G07*	chr19	14584205	14584205	A	G		0.381	Missense_Mutation	NM_000955.2	PTGER1	p.Trp310Arg	MODERATE	D	D	D	D	D	.	.	.	.	.	.	.	.	.	.
G14*	chr1	186644449	186644449	T	C	.	0.290	Missense_Mutation	NM_000963.3	PTGS2	p.Tyr446Cys	MODERATE															
G16	chr12	112926888	112926888	G	C	COSM1302	0.338	Missense_Mutation	NM_002834.3	PTPN11	p.Gly503Ala	MODERATE	D	D	D	D	D	.	.	.	.	.	.	.	.	.	.
G14*	chr12	112888199	112888199	C	T	COSM1301	0.457	Missense_Mutation	NM_002834.3	PTPN11	p.Ala72Val	MODERATE	D	D	D	P	D	.	.	.	.	.	.	.	.	.	.
G10*	chr9	8341247	8341247	G	C		0.888	Missense_Mutation	NM_002839.3	PTPRD	p.His1657As	MODERATE	D	D	P	P	T	.	.	.	.	.	.	.	.	.	.
G03	chr9	8504363	8504363	C	G		0.469	Missense_Mutation	NM_002839.3	PTPRD	p.Gly574Arg	MODERATE	D	D	P	P	T	PASS	3.29E-05	0	0	0.000462	0	0	0	0	0.00136
G05*	chr9	8504363	8504363	C	G		0.501	Missense_Mutation	NM_002839.3	PTPRD	p.Gly574Arg	MODERATE	D	D	P	P	T	PASS	3.29E-05	0	0	0.000462	0	0	0	0	0.00136
G05*	chr3	62263281	62263281	C	T	COSM47191	0.472	Missense_Mutation	NM_002841.3	PTPRG	p.Thr1231Me	MODERATE	D	N	D	D	T	.	.	.	.	.	.	.	.	.	0.00545
G07*	chr3	62189279	62189279	G	A		0.295	Missense_Mutation	NM_002841.3	PTPRG	p.Glu604Lys	MODERATE	D	N	P	B	T	.	.	.	.	.	.	.	.	.	.
G14*	chr8	30889616	30889616	C	T	.	0.747	Missense_Mutation	NM_001323311.1	PURG	p.Gly228Glu	MODERATE															

G14*	chr6	36690171	36690171	G	T	.	0.455	Missense_Mutation	NM_001257357.1	RAB44	p.Ala754Ser	MODERATE															
G07*	chr7	22233558	22233558	C	A		0.398	Missense_Mutation	NM_012294.3	RAPGEF5	p.Ala211Ser	MODERATE	N	N	B	B	T	.	.	.	.	.	.	.	.	.	.
G08	chr19	15564171	15564171	C	T		0.306	Missense_Mutation	NM_022904.1	RASAL3	p.Arg806Gln	MODERATE	N	N	B	B	T	.	.	.	.	.	.	.	.	.	.
G14*	chr19	15563505	15563505	C	T	.	0.413	Missense_Mutation	NM_022904.1	RASAL3	p.Gly931Asp	MODERATE															
G13	chr18	77802156	77802156	G	C		0.414	Missense_Mutation	NM_024805.2	RBFA	p.Gln171His	MODERATE	D	D	B	B	T	PASS	8.26E-06	0	0	0.000116	0	0	0	0	.
G07*	chr18	77806076	77806076	C	T		0.139	Missense_Mutation	NM_024805.2	RBFA	p.Pro318Leu	MODERATE	N	N	B	B	T	.	.	.	.	.	.	.	.	.	.
G16	chr22	36140246	36140246	G	A		0.368	Missense_Mutation	NM_001082577.1	RBFOX2	p.Thr370Met	MODERATE	D	N	D	D	D	PASS	5.90E-05	0	0	0.000701	0	0	6.33E-05	0	0.00045
G02	chr22	36142530	36142530	AAGCGGCT	A		0.486	Indel	NM_001082578.1	RBFOX2	p.Ala407_Ala	MODERATE	.	.	.	.	.	PASS	0.0010305	0.001179	0.00104	0.000696	0.00575	0.000733	0.000354	0.01367	.
G10*	chr22	36140283	36140283	C	T		0.382	Missense_Mutation	NM_001082578.1	RBFOX2	p.Arg442Gln	MODERATE	D	N	D	P	D	.	.	.	.	.	.	.	.	.	.
G05*	chrX	114424875	114424875	T	C		0.992	Missense_Mutation	NM_001145346.1	RBMXL3	p.Tyr291His	MODERATE	N	N	D	D	D	PASS	9.38E-05	0	0	0.002227	0	0	0	0	.
G14*	chr6	112671407	112671407	C	T		0.483	Missense_Mutation	NM_001013734.2	RFPL4B	p.Pro166Leu	MODERATE	N	N	B	B	T	PASS	9.08E-05	9.66E-05	0.000173	0	0	0.00012	0	0.0022	.
G12*	chr2	46803298	46803298	A	G	.	0.113	Missense_Mutation	NM_012249.3	RHOQ	p.Asn92Asp	MODERATE															
G10*	chr2	46803298	46803298	A	G	.	0.140	Missense_Mutation	NM_012249.3	RHOQ	p.Asn92Asp	MODERATE															
G18*	chrX	119249711	119249711	A	C	.	0.111	Missense_Mutation	NM_139282.2	RHOXF1	p.Ile21Arg	MODERATE															
G16	chr3	77607299	77607299	G	A	COSM73157	0.583	Splice_Site	NM_001290040.1	ROBO2	p.Arg483Gln	MODERATE	.	N	B	B	T	PASS	1.67E-05	0	0	0.000117	0	0	6.07E-05	0	0.00045
G12*	chr3	77681802	77681802	G	A	.	0.130	Missense_Mutation	NM_001290040.1	ROBO2	p.Arg1317Lys	MODERATE															
G15	chr20	62325796	62325796	C	G		0.477	Missense_Mutation	NM_001283009.1	RTEL1	p.Leu1022Va	MODERATE	D	N	D	D	D	PASS	0.0001181	0	0	0.001636	0	0	0	0	0.00409
G16	chr20	62326770	62326770	G	A		0.327	Missense_Mutation	NM_001283009.1	RTEL1	p.Gly1197Ser	MODERATE	N	N	B	B	T	PASS	8.32E-05	0.000103	0	0.000356	0	6.45E-05	0.000109	0	0.00136
G14*	chr20	62324236	62324236	A	G	.	0.284	Missense_Mutation	NM_001283009.1	RTEL1	p.Asn911Asp	MODERATE															
G07*	chr8	92998514	92998514	G	A	COSM17064	0.667	Nonsense_Mutation	NM_001198679.1	RUNX1T1	p.Arg432*	HIGH	A	.	.	.	.	.	.	.	.	.	.	.	.	.	.
G15	chr1	237777856	237777856	G	C		0.419	Missense_Mutation	NM_001035.2	RYR2	p.Val1810Leu	MODERATE	.	.	.	.	.	PASS	8.30E-05	0	0	0.001161	0	0	0	0	0.00227
G10*	chr8	144874282	144874282	G	T		0.249	Missense_Mutation	NM_182706.4	SCRIB	p.Asp1512Glu	MODERATE	D	N	D	D	D	.	.	.	.	.	.	.	.	.	.
G07*	chr2	64863285	64863285	A	C		0.398	Missense_Mutation	NM_014755.2	SERTAD2	p.Leu241Val	MODERATE	D	N	D	P	D	.	.	.	.	.	.	.	.	.	.
G15	chr18	42618459	42618459	G	C	COSM12653	0.511	Missense_Mutation	NM_015559.2	SETBP1	p.Ser1337Thr	MODERATE	N	N	P	B	D	PASS	8.25E-05	0	0	0.001159	0	0	0	0	0.00045
G04	chr3	47098547	47098547	G	A		0.346	Nonsense_Mutation	NM_014159.6	SETD2	p.Gln2243*	HIGH	A	.	.	.	.	.	.	.	.	.	.	.	.	.	.
G13	chr3	47103760	47103760	CT	C		0.287	Indel	NM_014159.6	SETD2	p.Glu2062fs	HIGH	.	.	.	.	.	.	.	.	.	.	.	.	.	.	.
G13	chr3	47103762	47103762	CTCTTGAC	C		0.201	Indel	NM_014159.6	SETD2	p.Arg2042_A	MODERATE	.	.	.	.	.	.	.	.	.	.	.	.	.	.	.
G06	chr3	47125222	47125222	C	A	COSM11862	0.232	Missense_Mutation	NM_014159.6	SETD2	p.Trp2016Cys	MODERATE	D	D	D	D	D	.	.	.	.	.	.	.	.	.	.
G13	chr3	47125235	47125235	A	T		0.452	Missense_Mutation	NM_014159.6	SETD2	p.Leu2012His	MODERATE	D	D	D	D	D	.	.	.	.	.	.	.	.	.	.
G02	chr3	47125763	47125763	GGAGGGA(	G		0.168	Indel	NM_014159.6	SETD2	p.Ser1828fs	HIGH	.	.	.	.	.	.	.	.	.	.	.	.	.	.	.
G09	chr3	47142970	47142970	C	A		0.670	Missense_Mutation	NM_014159.6	SETD2	p.Asp1665Ty	MODERATE	D	D	D	D	D	.	.	.	.	.	.	.	.	.	.
G02	chr3	47142975	47142975	G	T		0.412	Missense_Mutation	NM_014159.6	SETD2	p.Thr1663Lys	MODERATE	D	D	D	D	D	.	.	.	.	.	.	.	.	.	.
G04	chr3	47165773	47165773	ATTTTCAT	A		0.158	Indel	NM_014159.6	SETD2	p.Asn102fs	HIGH	.	.	.	.	.	.	.	.	.	.	.	.	.	.	.
G14*	chr3	47147505	47147505	C	T		0.489	Missense_Mutation	NM_014159.6	SETD2	p.Met1607Ile	MODERATE	D	D	D	D	D	.	.	.	.	.	.	.	.	.	.
G14*	chr3	47165189	47165189	G	A		0.462	Nonsense_Mutation	NM_014159.6	SETD2	p.Gln313*	HIGH	A	.	.	.	.	.	.	.	.	.	.	.	.	.	.
G03	chr8	8176424	8176424	C	T		0.544	Missense_Mutation	NM_001080826.2	SGK223	p.Cys1154Tyr	MODERATE	D	D	D	D	T	PASS	0.0004412	0	0	0.0049	0	0	0.000612	0	0.00455
G06	chr8	8233998	8233998	C	G		0.483	Missense_Mutation	NM_001080826.2	SGK223	p.Glu641Gln	MODERATE	D	N	B	B	D	.	.	.	.	.	.	.	.	.	0.00136
G04	chr8	8235227	8235227	G	A		0.431	Missense_Mutation	NM_001080826.2	SGK223	p.Ser231Phe	MODERATE	N	D	D	P	D	.	.	.	.	.	.	.	.	.	.
G05*	chr8	8234868	8234868	C	CGCCACT	.	0.215	Indel	NM_001080826.2	SGK223	p.Gly350_Ala	MODERATE															
G04	chr12	111885286	111885286	C	T	COSM43847	0.465	Missense_Mutation	NM_005475.2	SH2B3	p.Arg392Trp	MODERATE	D	D	D	D	D	PASS	1.67E-05	0	8.69E-05	0	0	1.52E-05	0	0.00112	.
G10*	chr19	52146624	52146624	G	T	.	0.107	Missense_Mutation	NM_001098612.1	SIGLEC14	p.Ala392Asp	MODERATE															
G08	chr18	43221207	43221207	A	G		0.374	Missense_Mutation	NM_001242692.1	SLC14A2	p.Tyr342Cys	MODERATE	D	N	D	D	D	.	.	.	.	.	.	.	.	.	.
G07*	chr18	43247932	43247932	G	A		0.352	Missense_Mutation	NM_001242692.1	SLC14A2	p.Gly618Ser	MODERATE	D	D	D	D	T	.	.	.	.	.	.	.	.	.	.
G03	chr9	19786836	19786836	G	A		0.516	Missense_Mutation	NM_020344.3	SLC24A2	p.Thr10Ile	MODERATE	N	N	B	B	T	PASS	9.96E-05	0	0	0.00139	0	0	0	0	0.00091
G14*	chr9	19520961	19520961	G	A	.	0.495	Missense_Mutation	NM_020344.3	SLC24A2	p.Ala556Val	MODERATE															
G01	chr3	48664355	48664355	C	T		0.466	Missense_Mutation	NM_022911.2	SLC26A6	p.Gly676Asp	MODERATE	N	N	P	P	D	PASS	8.29E-06	0	0	0.000116	0	0	0	0	.
G10*	chr3	48667109	48667109	G	A	COSM12261	0.163	Missense_Mutation	NM_022911.2	SLC26A6	p.Thr522Met	MODERATE	D	D	D	D	D	PASS	1.68E-05	0	0	0.000232	0	0	0	0	.
G11	chr1	205901160	205901160	G	T		0.414	Missense_Mutation	NM_134325.2	SLC26A9	p.Thr127Asn	MODERATE	.	.	.	.	.	PASS	6.64E-05	0	0	0.000925	0	0	0	0	0.00318
G14*	chr1	205899025	205899025	C	T	COSM45768	0.320	Missense_Mutation	NM_134325.2	SLC26A9	p.Val238Ile	MODERATE	.	.	.	.	.	PASS	4.24E-05	0	0.000173	0	0.00015	1.52E-05	7.01E-05	0.00231	.
G07*	chr15	45803375	45803375	A	G		0.464	Missense_Mutation	NM_013309.5	SLC30A4	p.Ser167Pro	MODERATE	D	N	B	B	T	.	.	.	.	.	.	.	.	.	.
G14*	chr17	70845856	70845856	C	T		0.444	Missense_Mutation	NM_001159770.1	SLC39A11	p.Arg180Gln	MODERATE	N	N	B	B	T	PASS	2.50E-05	0	0	0.000232	0	1.51E-05	0	0	.
G18*	chr12	105322218	105322218	A	C		0.202	Missense_Mutation	NM_032148.3	SLC41A2	p.Leu30Val	MODERATE	D	N	B	B	T	.	.	.	.	.	.	.	.	.	.
G14*	chr12	105280158	105280158	C	G	.	0.426	Missense_Mutation	NM_032148.3	SLC41A2	p.Val314Leu	MODERATE															
G14*	chr2	40392000	40392000	T	A		0.419	Missense_Mutation	NM_021097.2	SLC8A1	p.Glu721Asp	MODERATE	D	N	B	B	T	.	.	.	.	.	.	.	.	.	.
G18*	chr17	33738928	33738928	G	A		0.417	Missense_Mutation	NM_001289009.1	SLFN12	p.Thr389Met	MODERATE	N	N	B	B	T	PASS	0.0001019	0	0	0.001173	0	3.05E-05	0	0	0.00455
G07*	chr17	33738538	33738538	G	C		0.394	Missense_Mutation	NM_001289009.1	SLFN12	p.Pro519Arg	MODERATE	N	D	D	D	D	.	.	.	.	.	.	.	.	.	.
G07*	chr19	11150137	11150137	A	G		0.516	Missense_Mutation	NM_001128849.1	SMARCA4	p.Ile1392Val	MODERATE	N	N	B	B	T	PASS	2.65E-05	0.000156	0	0.000187	0	0	0	0	0.00091
G09	chr18	2697038	2697038	A	G		0.390	Missense_Mutation	NM_015295.2	SMCHD1	p.Tyr350Cys	MODERATE	D	D	D	D	D	.	.	.	.	.	.	.	.	.	.
G15	chr16	11348876	11348876	AGTGCTCC	A		0.124	Indel	NM_003745.1	SOCS1	p.Cys146fs	HIGH	.	.	.	.	.	.	.	.	.	.	.	.	.	.	.
G14*	chr2	39222453	39222453	G	A		0.439	Missense_Mutation	NM_005633.3	SOS1	p.Pro1053Ser	MODERATE	D	D	D	D	D	.	.	.	.	.	.	.	.	.	.
G15	chr5	147477531	147477531	C	G		0.209	Missense_Mutation	NM_001127698.1	SPINK5	p.Asn328Lys	MODERATE	N	D	D	D	D	.	.	.	.	.	.	.	.	.	.
G05*	chr5	147503405	147503405	C	T	COSM46203	0.389	Missense_Mutation	NM_001127698.1	SPINK5	p.Arg850Cys	MODERATE	N	N	D	P	D	PASS	7.49E-05	0	0	0.000698	0	4.52E-05	0	0	0.00091
G07*	chr5	147465987	147465987	A	G		0.282	Missense_Mutation	NM_001127698.1	SPINK5	p.Lys101Arg	MODERATE	N	N	B	B	T	.	.	.	.	.	.	.	.	.	.
G07*	chr7	75912331	75912331	C	T	.	0.182	Missense_Mutation	NM_001291831.1	SRRM3	p.Arg549Cys	MODERATE															
G06	chr2	191851674	191851674	C	T		0.383	Splice_Site	NM_007315.3	STAT1		HIGH	D	.	.	.	.	.	.	.	.	.	.	.	.	.	.
G15	chr17	40475060	40475060	CCTT	C	COSM31886	0.100	Indel	NM_139276.2	STAT3	p.Glu616del	MODERATE	.	.	.	.	.	.	.	.	.	.	.	.	.	.	.
G04	chr17	40456416	40456416	C	T		0.639	Missense_Mutation	NM_001288718.1	STAT5A	p.Thr409Met	MODERATE	D	D	D	P	D	PASS	0.000198	0	0	0.002775	0	0	0	0	0.00227
G02	chr17	40359729	40359729	T	G	COSM17165	0.583	Missense_Mutation	NM_012448.3	STAT5B	p.Asn642His	MODERATE	D	D	D	D	T	.	.	.	.	.	.	.	.	.	.
G03	chr17	40375561	40375561	G	A		0.485	Missense_Mutation	NM_012448.3	STAT5B	p.Ala130Val	MODERATE	N	N	B	B	T	PASS	0.0002142	0	0	0.003004	0	0	0	0	0.00864
G01	chr1	172520665	172520665	C	T		0.446	Missense_Mutation	NM_016227.3	SUCO	p.Arg221Cys	MODERATE	.	.	.	.	.	PASS	0.0001263	9.92E-05	0.000178	0	0	0.000184	0	0.00223	.
G01	chr1	172558304	172558304	C	T		0.199	Missense_Mutation	NM_016227.3	SUCO	p.Thr839Met	MODERATE	.	.	.	.	.	PASS	2.48E-05	0	0.000174	0	0	1.50E-05	0	0.00221	.
G18*	chr1	172559045	172559045	A	G	.	0.109	Missense_Mutation	NM_016227.3	SUCO	p.Tyr1086Cys	MODERATE															
G14*	chr19	19130027	19130027	G	A	COSM29297	0.143	Nonsense_Mutation	NM_001321698.1	SUGP2	p.Arg595*	HIGH															
G07*	chr14	21821918	21821918	T	C		0.336	Missense_Mutation	NM_007192.3	SUPT16H	p.Tyr955Cys	MODERATE	D	N	B	B	T	PASS	8.25E-06	0	0	0	0	0	6.10E-05	0	.
G06	chr17	30293169	30293169	G	T		0.189	Missense_Mutation	NM_015355.3	SUZ12	p.Leu153Phe	MODERATE	D	N	B	B	T	.	.	.	.	.	.	.	.	.	.
G07*	chr10	71175934	71175934	G	A	COSM47571	0.620	Missense_Mutation	NM_001057.2	TACR2	p.Thr49Met	MODERATE	N	N	B	B	T	PASS	0.0001896	0.001636	8.64E-05	0	0	7.50E-05	0	0.00111	.
G10*	chr12	11183042	11183042	C	A	.	0.163	Missense_Mutation	NM_176885.2	TAS2R31	p.Arg298Met	MODERATE															
G13	chr9	100193303	100193303	G	A		0.459	Missense_Mutation	NM_014290.2	TDRD7	p.Arg99His	MODERATE	D	N	P	B	T	PASS	3.30E-05	9.62E-05	0	0.000347	0	0	0	0	0.00045
G14*	chr9	100235879	100235879	C	T	.	0.193	Missense_Mutation	NM_014290.2	TDRD7	p.Arg684Cys	MODERATE															
G14*	chr9	27157963	27157963	G	A	.	0.444	Missense_Mutation	NM_000459.4	TEK	p.Ala63Thr	MODERATE															
G18*	chr10	70333006	70333006	A	G		0.205	Missense_Mutation	NM_030625.2	TET1	p.Lys304Arg	MODERATE	N	N	B	B	T	.	.	.	.	.	.	.	.	.	.
G08	chr4	106197167	106197167	C	T	COSM42014	0.469	Nonsense_Mutation	NM_001127208.2	TET2	p.Gln1834*	HIGH	D	.	.	.	.	.	.	.	.	.	.	.	.	.	.
G08	chr4	106158509	106158509	G	GT		0.299	Indel	NM_017628.4	TET2	p.Lys1138fs	HIGH	.	.	.	.	.	.	.	.	.	.	.	.	.	.	.
G15	chr4	106196285	106196285	C	T	COSM17249	0.155	Nonsense_Mutation	NM_001127208.2	TET2	p.Gln1540*	HIGH	D	.	.	.	.	.	.	.	.	.	.	.	.	.	.

G10*	chr2	137814193	137814193	G	A		0.114	Missense_Mutation	NM_001316349.1	THSD7B	p.Val115Met	MODERATE	N	N	B	B	T	.	.	.	.	.	.	.	.	.	.
G05*	chr14	105063354	105063354	A	G	.	0.132	Missense_Mutation	NM_001286389.1	TMEM179	p.Ile139Thr	MODERATE															
G14*	chrX	34675117	34675117	C	A		0.493	Missense_Mutation	NM_031442.3	TMEM47	p.Glu10Asp	MODERATE	D	N	B	B	T	.	.	.	.	.	.	.	.	.	.
G16	chr9	100328223	100328223	G	T		0.769	Missense_Mutation	NM_001166116.1	TMOD1	p.Asp238Tyr	MODERATE	D	D	D	D	D	.	.	.	.	.	.	.	.	.	.
G08	chr17	7577060	7577060	CCTTT	C	COSM1480(	0.367	Indel	NM_000546.5	TP53	p.Lys292fs	HIGH	.	.	.	.	.	.	.	.	.	.	.	.	.	.	.
G01	chr17	7577562	7577562	C	CTGTT		0.305	Indel	NM_000546.5	TP53	p.Ser240fs	HIGH	.	.	.	.	.	.	.	.	.	.	.	.	.	.	.
G02	chr17	7578253	7578253	C	A	COSM2557ξ	0.729	Missense_Mutation	NM_000546.5	TP53	p.Gly199Val	MODERATE	D	D	D	D	D	.	.	.	.	.	.	.	.	.	.
G07*	chr17	7577559	7577559	G	A	COSM1081Z	0.633	Missense_Mutation	NM_000546.5	TP53	p.Ser241Phe	MODERATE	A	D	D	D	D	.	.	.	.	.	.	.	.	.	.
G18*	chr17	7578291	7578291	T	G	COSM3239Z	0.109	Splice_Site	NM_000546.5	TP53		HIGH	D	.	.	.	.	.	.	.	.	.	.	.	.	.	.
G16	chr8	141231575	141231575	C	T		0.457	Missense_Mutation	NM_031466.7	TRAPPC9	p.Asp945Asn	MODERATE	D	N	D	P	T	PASS	0.0001071	0	0	0.001502	0	0	0	0	0.00545
G18*	chr8	140743452	140743452	G	A	COSM1455I	0.205	Missense_Mutation	NM_031466.7	TRAPPC9	p.Ser1198Leu	MODERATE	D	N	B	B	T	PASS	8.54E-06	0	0	0.000118	0	0	0	0	.
G12*	chr1	231299470	231299470	AGCCGCCC	A	.	0.114	Indel	NM_001004342.3	TRIM67	p.Pro258_Pro	MODERATE															
G07*	chr22	45683191	45683191	T	G		0.637	Missense_Mutation	NM_006953.3	UPK3A	p.Leu116Arg	MODERATE	D	N	D	D	D	.	.	.	.	.	.	.	.	.	.
G07*	chr2	203760810	203760810	G	A		0.284	Missense_Mutation	NM_018256.3	WDR12	p.Ala196Val	MODERATE	D	D	D	P	D	.	.	.	.	.	.	.	.	.	.
G07*	chr6	170070666	170070666	T	A		0.649	Splice_Site	NM_182552.4	WDR27	p.Glu152Val	MODERATE	D	D	D	P	D	.	.	.	.	.	.	.	.	.	.
G07*	chr1	241815913	241815913	T	A		0.393	Missense_Mutation	NM_144625.4	WDR64	p.Leu43Ile	MODERATE	.	.	.	.	.	.	.	.	.	.	.	.	.	.	.
G10*	chr4	44631430	44631430	G	C		0.425	Missense_Mutation	NM_182592.2	YIPF7	p.Thr163Ser	MODERATE	D	.	.	.	.	.	.	.	.	.	.	.	.	.	.
G07*	chr7	150027968	150027968	G	A		0.546	Missense_Mutation	NM_138434.2	ZBED6CL	p.Ala159Thr	MODERATE	N	N	B	B	T	.	.	.	.	.	.	.	.	.	.
G08	chr18	45567020	45567020	G	T		0.139	Missense_Mutation	NM_001039360.2	ZBTB7C	p.Asp153Glu	MODERATE	N	N	B	B	T	.	.	.	.	.	.	.	.	.	.
G08	chr18	45567028	45567028	C	T		0.101	Missense_Mutation	NM_001039360.2	ZBTB7C	p.Glu151Lys	MODERATE	D	N	B	B	T	.	.	.	.	.	.	.	.	.	.
G13	chr18	45567029	45567029	A	C		0.121	Missense_Mutation	NM_001039360.2	ZBTB7C	p.Asp150Glu	MODERATE	N	N	B	B	T	PASS	0.0003399	0.000482	8.70E-05	0.000116	0.00091	0.000392	0.000123	0.00111	.
G10*	chr18	45555734	45555734	C	T	COSM9886G	0.396	Missense_Mutation	NM_001039360.2	ZBTB7C	p.Arg586Gln	MODERATE	N	N	B	B	T	.	.	.	.	.	.	.	.	.	.
G18*	chr8	77766798	77766798	G	A	COSM5645ξ	0.141	Missense_Mutation	NM_024721.4	ZFHX4	p.Met2547Ile	MODERATE	D	N	B	B	D	.	.	.	.	.	.	.	.	.	.
G14*	chr8	77761809	77761809	T	C		0.371	Missense_Mutation	NM_024721.4	ZFHX4	p.Val1236Ala	MODERATE	D	N	P	B	T	.	.	.	.	.	.	.	.	.	.
G10*	chr8	77690583	77690583	G	A	COSM4899Z	0.278	Missense_Mutation	NM_024721.4	ZFHX4	p.Arg1078Gln	MODERATE	D	D	D	D	D	.	.	.	.	.	.	.	.	.	.
G18*	chr9	109690611	109690611	C	T	.	0.130	Missense_Mutation	NM_021224.4	ZNF462	p.Thr1473Me	MODERATE															
G10*	chr19	52443882	52443882	G	A	.	0.245	Missense_Mutation	NM_001031721.3	ZNF613	p.Ser52Asn	MODERATE															
G07*	chr19	21476647	21476647	TTAAAAGC	T	.	0.417	Indel	NM_021269.2	ZNF708	p.Ser346_Phe	MODERATE															
G04	chr19	58190172	58190172	T	C		0.450	Missense_Mutation	NM_152677.2	ZSCAN4	p.Cys401Arg	MODERATE	N	D	D	D	D	.	.	.	.	.	.	.	.	.	.
G07*	chr19	58190166	58190166	C	T		0.463	Missense_Mutation	NM_152677.2	ZSCAN4	p.Pro399Ser	MODERATE	N	N	B	B	T	.	.	.	.	.	.	.	.	.	.
G18*	chr1	78034049	78034049	C	T	.	0.190	Missense_Mutation	NM_015534.5	ZZZ3	p.Glu812Lys	MODERATE															

* Somatic mutations of whole exome sequencing were included.
