# Supplementary material for: Characteristic gene alterations in primary gastrointestinal T- and NK-cell lymphomas
Source: Leukemia. 2019 Jan 23;33(7):1797–832. doi: 10.1038/s41375-018-0309-4 (PMC6755973; doi:10.1038/s41375-018-0309-4)
Supplement: Supplementary file 21 — Supplementary table 7 [file 41375_2018_309_MOESM21_ESM.pdf]

Supplementary Table 7. non-GI-TNKL Somatic Mutations

Patient ID	Chromosome	Start	End	Ref	Alt	Cosmic	Variant allele frequency	Mutation type	Transcription ID	Gene	Amino acid change	SnpEff Impact	MutationTaster_pred	PROVEAN_pred	Polyphen2_HDIV_pred	Polyphen2_HVAR_pred	SIFT_pred	FILTER_ExAC	AF_TOTAL	AF_AFR	AF_AMR	AF_EAS	AF_FIN	AF_NFE	AF_SAS	AF_OTH	AF_KoreaRef
N46	chr16	1389534	1389534	T	C		0.462750716	Missense_Mutation	NM_003933.4	BAIAP3	p.Leu148Pro	MODERA	N	D	P	B	T	.	.	.	.	.	.	.	.	.	.
N26	chr16	1394080	1394080	C	T		0.213114754	Missense_Mutation	NM_003933.4	BAIAP3	p.Arg517Cys	MODERA	D	D	D	P	D	PASS	1.68E-05	0	0	0.000233	0	0	0	0	0.001364
N41	chr16	1397585	1397585	C	T		0.540772532	Missense_Mutation	NM_003933.4	BAIAP3	p.Arg990Cys	MODERA	N	D	B	B	T	PASS	0.0002481	0	0	0.003168	0	3.35E-05	0	0	0.006818
N64	chr7	2962827	2962827	G	A	COSM550	0.732758621	Missense_Mutation	NM_001324281.1	CARD11	p.Ser694Leu	MODERA	D	N	B	B	T	PASS	0.00034744	0	0	0.004682	0	0	6.07E-05	0	0.002727
N31	chr7	2969678	2969678	G	T		0.2	Missense_Mutation	NM_001324281.1	CARD11	p.Ala534Asp	MODERA	N	N	B	B	T	PASS	0.00015672	0	0	0.002197	0	0	0	0	.
N31	chr7	2969679	2969679	C	T		0.198863636	Missense_Mutation	NM_001324281.1	CARD11	p.Ala534Thr	MODERA	N	N	B	B	T	PASS	0.00015673	0	0	0.002197	0	0	0	0	.
N48	chr7	2976741	2976741	C	T		0.46969697	Missense_Mutation	NM_001324281.1	CARD11	p.Arg424Gln	MODERA	N	N	B	B	T	PASS	4.95E-05	0	0	0.000463	0	1.50E-05	6.06E-05	0	.
N14	chr1	3412514	3412514	C	G		0.456834532	Missense_Mutation	NM_001409.3	MEGF6	p.Asp1271His	MODERA	D	D	B	B	T	.	.	.	.	.	.	.	.	.	.
N44	chr1	3425702	3425702	C	T		0.455078125	Missense_Mutation	NM_001409.3	MEGF6	p.Asp489Asn	MODERA	N	N	P	B	T	PASS	0.00011109	0	0	0.000233	0	6.93E-05	0.000219	0	0.000455
N11	chr20	3654494	3654494	C	T		0.231578947	Missense_Mutation	NM_025220.4	ADAM33	p.Arg268His	MODERA	N	N	B	B	T	.	.	.	.	.	.	.	.	.	.
N76	chr16	3820912	3820912	G	T		0.545454545	Missense_Mutation	NM_004380.2	CREBBP	p.Pro847Thr	MODERA	D	N	B	B	T	PASS	6.59E-05	0	0	0.000925	0	0	0	0	0.000455
N52	chr16	3900710	3900710	G	A	COSM481	0.603773585	Missense_Mutation	NM_004380.2	CREBBP	p.Ser129Leu	MODERA	D	N	D	P	D	.	.	.	.	.	.	.	.	.	.
N76	chr9	5069922	5069922	CAG	C		0.236641221	Indel	NM_001322194.1	JAK2	p.Asp505fs	HIGH	.	.	.	.	.	.	.	.	.	.	.	.	.	.	.
N76	chr9	5069928	5069928	A	C		0.262411348	Missense_Mutation	NM_001322194.1	JAK2	p.Lys506Thr	MODERA	D	N	D	P	T	.	.	.	.	.	.	.	.	.	.
N76	chr9	5069930	5069930	T	C		0.262068966	Missense_Mutation	NM_001322194.1	JAK2	p.Ser507Pro	MODERA	D	D	D	D	D	.	.	.	.	.	.	.	.	.	.
N76	chr9	5069933	5069933	AACCTT	A		0.259493671	Indel	NM_001322194.1	JAK2	p.Asn508fs	HIGH	.	.	.	.	.	.	.	.	.	.	.	.	.	.	.
N76	chr9	5069939	5069939	C	CA		0.272151899	Indel	NM_001322194.1	JAK2	p.Leu510fs	HIGH	.	.	.	.	.	.	.	.	.	.	.	.	.	.	.
N76	chr9	5069941	5069941	AGT	A		0.275449102	Indel	NM_001322194.1	JAK2	p.Val511fs	HIGH	.	.	.	.	.	.	.	.	.	.	.	.	.	.	.
N76	chr9	5069948	5069948	A	G		0.300578035	Missense_Mutation	NM_001322194.1	JAK2	p.Arg513Gly	MODERA	D	D	D	D	D	.	.	.	.	.	.	.	.	.	.
N76	chr9	5069950	5069950	AAC	A		0.287958115	Indel	NM_001322194.1	JAK2	p.Thr514fs	HIGH	.	.	.	.	.	.	.	.	.	.	.	.	.	.	.
N76	chr9	5069954	5069954	A	G		0.287958115	Missense_Mutation	NM_001322194.1	JAK2	p.Asn515Asp	MODERA	D	N	B	B	T	.	.	.	.	.	.	.	.	.	.
N76	chr9	5069955	5069955	A	T		0.286458333	Missense_Mutation	NM_001322194.1	JAK2	p.Asn515Ile	MODERA	D	D	D	P	T	.	.	.	.	.	.	.	.	.	.
N66	chr18	5394782	5394782	T	C		0.504761905	Missense_Mutation	NM_012307.3	EPB41L3	p.Gln1055Arg	MODERA	D	N	D	D	D	PASS	9.07E-05	0	0	0.001271	0	0	0	0	0.006364
N22	chr18	5410605	5410605	C	T	COSM288	0.488372093	Missense_Mutation	NM_012307.3	EPB41L3	p.Arg694His	MODERA	D	N	D	P	D	PASS	0.00012404	0.000193	0	0.001393	0	0	6.09E-05	0	0.002727
N40	chr18	5416200	5416200	C	T	COSM363	0.302564103	Missense_Mutation	NM_012307.3	EPB41L3	p.Gly562Arg	MODERA	D	N	B	B	T	PASS	8.24E-06	0	0	0	0	0	6.06E-05	0	.
N40	chr19	5832431	5832431	A	C		0.296296296	Missense_Mutation	NM_000150.2	FUT6	p.Phe50Val	MODERA	N	N	B	B	T	.	.	.	.	.	.	.	.	.	.
N70	chr17	7479956	7479956	A	G		0.482084691	Splice_Site	NM_001416.3	EIF4A1	p.Ile154Val	MODERA	D	N	B	B	T	.	.	.	.	.	.	.	.	.	.
N52	chr17	7576857	7576857	A	C	COSM116	0.111111111	Missense_Mutation	NM_000546.5	TP53	p.Leu330Arg	MODERA	D	D	.	.	.	.	.	.	.	.	.	.	.	.	.
N55	chr17	7576880	7576880	TGGTTTCT	T		0.196721311	Indel	NM_000546.5	TP53	p.Ser314fs	HIGH	.	.	.	.	.	.	.	.	.	.	.	.	.	.	.
N68	chr17	7577534	7577534	C	A	COSM108	0.189655172	Missense_Mutation	NM_000546.5	TP53	p.Arg249Ser	MODERA	D	D	D	D	D	.	.	.	.	.	.	.	.	.	.
N46	chr17	7578534	7578534	C	G	COSM175	0.839506173	Missense_Mutation	NM_000546.5	TP53	p.Lys132Asn	MODERA	D	D	D	D	D	.	.	.	.	.	.	.	.	.	.
N12	chr17	8044540	8044540	C	T		0.503496503	Missense_Mutation	NM_002616.2	PER1	p.Ser1240Asn	MODERA	N	N	B	B	T	PASS	1.65E-05	0	0	0.000231	0	0	0	0	.
N46	chr17	8048254	8048254	GC	G		0.238738739	Indel	NM_002616.2	PER1	p.Ala759fs	HIGH	.	.	.	.	.	.	.	.	.	.	.	.	.	.	.
N52	chr17	8159874	8159874	G	A		0.615384615	Missense_Mutation	NM_012393.2	PFAS	p.Arg285Gln	MODERA	N	N	B	B	T	PASS	8.25E-05	0	0	0.00081	0	4.50E-05	0	0	0.000909
N51	chr17	8172118	8172118	T	C		0.592592593	Missense_Mutation	NM_012393.2	PFAS	p.Leu1217Pro	MODERA	D	D	P	P	D	PASS	2.58E-05	0	0	0.000359	0	0	0	0	0.001818
N43	chr8	8185660	8185660	C	T		0.370629371	Missense_Mutation	NM_001080826.2	SGK223	p.Asp878Asn	MODERA	D	N	B	B	D	PASS	2.49E-05	0	0	0.000232	0	1.50E-05	0	0	0.000455
N22	chr8	8234837	8234837	C	T		0.452196382	Missense_Mutation	NM_001080826.2	SGK223	p.Ser361Asn	MODERA	N	N	B	B	T	.	.	.	.	.	.	.	.	.	.
N51	chr8	8238926	8238926	A	G		0.486486486	Splice_Site	NM_001080826.2	SGK223		HIGH	D	.	.	.	.	PASS	7.73E-05	0	0	0.000929	0	1.53E-05	0	0	0.003636
N28	chr9	8338935	8338935	A	C		0.270072993	Missense_Mutation	NM_002839.3	PTPRD	p.Val1789Gly	MODERA	D	D	D	D	D	.	.	.	.	.	.	.	.	.	.
N36	chr1	8419868	8419868	GCTCCTT	G	COSM368	0.470588235	Indel	NM_001042681.1	RERE	p.Lys1190_Gl	MODERA	.	.	.	.	.	PASS	0.00401718	0.008532	0.005893	0.007005	0.000641	0.003363	0.002287	0.078014	.
N12	chr3	8579053	8579053	T	C		0.448979592	Missense_Mutation	NM_014583.3	LMCD1	p.Ile105Thr	MODERA	D	N	P	B	T	PASS	3.31E-05	0.000288	8.64E-05	0	0	0	0	0.001111	.
N31	chr3	8590329	8590329	C	T	COSM282	0.495967742	Missense_Mutation	NM_014583.3	LMCD1	p.Arg155Cys	MODERA	D	D	D	D	D	PASS	0.00034619	0	8.64E-05	0.004278	0	1.50E-05	0.000182	0.001101	0.008182
N69	chr3	8590329	8590329	C	T	COSM282	0.517799353	Missense_Mutation	NM_014583.3	LMCD1	p.Arg155Cys	MODERA	D	D	D	D	D	PASS	0.00034619	0	8.64E-05	0.004278	0	1.50E-05	0.000182	0.001101	0.008182
N28	chrX	10181963	10181963	C	T		0.979020979	Missense_Mutation	NM_001830.3	CLCN4	p.Pro607Ser	MODERA	D	N	B	B	T	PASS	0.00016646	0	0	0.002153	0	0	0	0	0.001818
N40	chr17	10351820	10351820	T	C		0.204301075	Missense_Mutation	NM_017533.2	MYH4	p.Thr1517Ala	MODERA	D	D	P	P	D	.	.	.	.	.	.	.	.	.	.
N11	chr17	10352331	10352331	T	G		0.289156627	Missense_Mutation	NM_017533.2	MYH4	p.Glu1405Asp	MODERA	D	N	D	D	D	.	.	.	.	.	.	.	.	.	.
N75	chr19	10472598	10472598	C	T	COSM473	0.456790123	Missense_Mutation	NM_003331.4	TYK2	p.Val603Met	MODERA	N	N	D	D	D	PASS	0.0002813	0.000194	0.000346	0.00081	0	0.000256	0.000242	0.004415	0.002727
N64	chr19	10472964	10472964	G	A		0.509247842	Splice_Site	NM_003331.4	TYK2	p.Arg549Cys	MODERA	N	D	D	P	D	PASS	0.00011596	0	0	0.001505	0	1.51E-05	0	0	0.002727
N68	chr16	10995975	10995975	C	G		0.533333333	Missense_Mutation	NM_001286402.1	CIITA	p.Pro189Ala	MODERA	N	N	B	B	T	.	.	.	.	.	.	.	.	.	.
N31	chr19	11094865	11094865	G	A		0.264637002	Missense_Mutation	NM_001128849.1	SMARCA	p.Arg13Gln	MODERA	D	N	P	B	D	PASS	8.47E-06	0	0	0	0	1.54E-05	0	0	.
N68	chr19	11095973	11095973	G	A		0.226666667	Missense_Mutation	NM_001128849.1	SMARCA	p.Gly83Ser	MODERA	D	D	B	B	T	PASS	1.66E-05	9.68E-05	8.66E-05	0	0	0	0	0.001111	.
N51	chr19	11097111	11097111	A	T		0.3375	Missense_Mutation	NM_001128849.1	SMARCA	p.Gln201Leu	MODERA	D	N	P	D	T	PASS	0.00040264	0	0	0.006831	0	0	0.000115	0	0.004545
N11	chr19	11123691	11123691	A	G		0.225806452	Missense_Mutation	NM_001128849.1	SMARCA	p.Met781Val	MODERA	D	D	D	D	D	.	.	.	.	.	.	.	.	.	.
N22	chr16	11348988	11348988	G	T	COSM158	0.105882353	Missense_Mutation	NM_003745.1	SOCS1	p.Ser116Arg	MODERA	D	D	D	D	D	.	.	.	.	.	.	.	.	.	.
N11	chr16	11349150	11349150	GGCGT	G		0.268292683	Indel	NM_003745.1	SOCS1	p.His61fs	HIGH	.	.	.	.	.	.	.	.	.	.	.	.	.	.	.
N28	chr16	11349214	11349214	GGCCGCG	G		0.145748988	Indel	NM_003745.1	SOCS1	p.Ala16fs	HIGH	.	.	.	.	.	.	.	.	.	.	.	.	.	.	.
N40	chr19	11489104	11489104	A	AAATGGTTG		0.102941176	Indel	NM_000121.3	EPOR	p.Glu362fs	HIGH	.	.	.	.	.	.	.	.	.	.	.	.	.	.	.
N69	chr12	11506654	11506654	C	CTGTTGCCTCCTTG		0.991440799	Indel	NM_005039.3	PRB1	p.Asn127_Arg	MODERA	.	.	.	.	.	.	.	.	.	.	.	.	.	.	.
N43	chr12	11506862	11506862	C	G		0.438596491	Missense_Mutation	NM_005039.3	PRB1	p.Gly59Arg	MODERA	N	D	B	B	T	.	.	.	.	.	.	.	.	.	.
N26	chr19	14584220	14584220	C	T		0.426229508	Missense_Mutation	NM_000955.2	PTGER1	p.Val305Met	MODERA	N	N	D	D	D	.	.	.	.	.	.	.	.	.	.
N76	chr18	14803745	14803745	A	G	COSM152	0.159663866	Missense_Mutation	NM_001145029.1	ANKRD3	p.Thr736Ala	MODERA	N	N	B	B	D	.	.	.	.	.	.	.	.	.	0.003182
N66	chr16	15808856	15808856	T	C		0.527542373	Missense_Mutation	NM_001040114.1	MYH11	p.Asn1906Ser	MODERA	D	N	B	B	T	PASS	0.0003383	0	0	0.00463	0	0	6.06E-05	0	0.005
N48	chr16	15815346	15815346	C	A		0.473684211	Missense_Mutation	NM_001040114.1	MYH11	p.Arg1511Leu	MODERA	D	D	D	D	D	.	.	.	.	.	.	.	.	.	.
N68	chr16	15831439	15831439	C	G		0.6	Missense_Mutation	NM_001040114.1	MYH11	p.Glu1061Gln	MODERA	D	N	D	D	D	.	.	.	.	.	.	.	.	.	.
N73	chr22	19178928	19178928	C	T		0.468365554	Missense_Mutation	NM_007098.3	CLTCL1	p.Cys1404Tyr	MODERA	N	N	B	B	T	.	.	.	.	.	.	.	.	.	0.000455
N52	chr22	19184017	19184017	G	A		0.489361702	Missense_Mutation	NM_007098.3	CLTCL1	p.Arg1342Cys	MODERA	D	D	D	D	D	PASS	7.47E-05	0.000307	0.000173	0	0	1.50E-05	0.000182	0.002237	.
N73	chr22	19188995	19188995	G	C		0.475806452	Missense_Mutation	NM_007098.3	CLTCL1	p.Arg1204Gly	MODERA	D	D	P	P	D	PASS	0.00017349	0	0	0.001082	0	0	0	0	0.000909
N28	chr9	19785967	19785967	C	T		0.297709924	Missense_Mutation	NM_020344.3	SLC24A2	p.Val300Ile	MODERA	D	N	B	B	T	PASS	5.77E-05	0.000192	0	0	0	5.99E-05	6.06E-05	0	.
N14	chr14	21108943	21108943	G	C		0.456140351	Missense_Mutation	NM_001001968.1	OR6S1	p.Ala303Gly	MODERA	N	D	P	B	D	.	.	.	.	.	.	.	.	.	.
N46	chr17	21318949	21318949	G	A		0.18131031	Missense_Mutation	NM_021012.4	KCNJ12	p.Gly99Ser	MODERA	D	D	D	D	D	PASS	1.65E-05	9.62E-05	0	0.000116	0	0	0	0	0.000455
N46	chr17	21319156	21319156	G	A		0.206451613	Missense_Mutation	NM_021012.4	KCNJ12	p.Val168Met	MODERA	D	N	P	B	T	PASS	0.00012377	9.64E-05	0	0.000926	0	3.00E-05	0.000242	0	.
N51	chr14	21854022	21854022	G	T	COSM296	0.151515152	Missense_Mutation	NM_001170629.1	CHD8	p.Pro2499His	MODERA	N	N	B	B	T	.	.	.	.	.	.	.	.	.	.
N36	chr14	21860015	21860015	CCTT	C	COSM202	0.492401216	Indel	NM_001170629.1	CHD8	p.Lys2287del	MODERA	.	.	.	.	.	PASS	4.98E-05	0	0	0.000696	0	0	0	0	.
N55	chr14	21881093	21881093	A	C		0.145454545	Missense_Mutation	NM_001170629.1	CHD8	p.Ser735Arg	MODERA	D	N	P	P	T	.	.	.	.	.	.	.	.	.	.
N55	chr14	21881094	21881094	C	CTTTATTTT		0.145454545	Indel	NM_001170629.1	CHD8	p.Ser735fs	HIGH	.	.	.	.	.	.	.	.	.	.	.	.	.	.	.

N11	chr9	21971120	21971120	G	A	COSM124	0.740384615	Nonsense_Mutation	NM_001195132.1	CDKN2A	p.Arg80*	HIGH	A	D	D	B	T	.	.	.	.	.	.	.	.	.	.
N40	chr9	21971186	21971186	G	A	COSM124	0.31292517	Nonsense_Mutation	NM_001195132.1	CDKN2A	p.Arg58*	HIGH	A	D	P	B	D	.	.	.	.	.	.	.	.	.	.
N75	chr7	22186573	22186573	C	T		0.159235669	Nonsense_Mutation	NM_012294.3	RAPGEF5	p.Trp497*	HIGH	A	.	.	.	.	.	.	.	.	.	.	.	.	.	.
N46	chr22	23627261	23627261	C	T		0.535603715	Missense_Mutation	NM_004327.3	BCR	p.Thr760Met	MODERA	D	N	P	B	D	PASS	4.99E-05	0	8.72E-05	0.000579	0	0	0	0.001121	0.002727
N70	chr12	25398284	25398284	C	G	COSM114	0.411371237	Missense_Mutation	NM_033360.3	KRAS	p.Gly12Ala	MODERA	D	D	P	P	D	.	.	.	.	.	.	.	.	.	.
N48	chr2	25457243	25457243	G	A	COSM116	0.488095238	Missense_Mutation	NM_022552.4	DNMT3A	p.Arg882Cys	MODERA	D	D	D	D	D	PASS	0.00035508	0.000677	8.66E-05	0.000347	0	0.000391	0.000364	0.001106	.
N51	chr2	25457243	25457243	G	T	COSM438	0.177631579	Missense_Mutation	NM_022552.4	DNMT3A	p.Arg882Ser	MODERA	D	D	D	P	D	.	.	.	.	.	.	.	.	.	.
N14	chr22	26231363	26231363	T	C		0.447368421	Missense_Mutation	NM_001318245.1	MYO18B	p.Leu1055Pro	MODERA	D	D	D	D	D	PASS	8.28E-05	0	0	0.001159	0	0	0	0	0.004091
N14	chr22	26422632	26422632	G	A	COSM444	0.418032787	Missense_Mutation	NM_001318245.1	MYO18B	p.Arg2232Glr	MODERA	N	.	.	.	.	PASS	1.68E-05	0	0	0	0	1.52E-05	6.11E-05	0	.
N12	chr1	27088744	27088744	A	T		0.108695652	Missense_Mutation	NM_006015.4	ARID1A	p.Met785Leu	MODERA	D	N	P	P	D	.	.	.	.	.	.	.	.	.	.
N75	chr1	27101342	27101342	G	T	COSM514	0.256097561	Nonsense_Mutation	NM_006015.4	ARID1A	p.Glu1542*	HIGH	A	.	.	.	.	.	.	.	.	.	.	.	.	.	.
N70	chr1	27106410	27106410	GC	G		0.263392857	Indel	NM_006015.4	ARID1A	p.Leu2008fs	HIGH	.	.	.	.	.	.	.	.	.	.	.	.	.	.	.
N40	chr1	27106661	27106661	G	A		0.376811594	Nonsense_Mutation	NM_006015.4	ARID1A	p.Trp2091*	HIGH	D	.	.	.	.	.	.	.	.	.	.	.	.	.	.
N23	chr9	27204927	27204927	G	A		0.392857143	Missense_Mutation	NM_000459.4	TEK	p.Gly743Glu	MODERA	D	N	B	B	T	PASS	8.24E-06	0	0	0	0	1.50E-05	0	0	.
N14	chr1	27701275	27701275	A	G		0.526315789	Missense_Mutation	NM_003665.3	FCN3	p.Leu12Pro	MODERA	N	N	D	P	D	.	.	.	.	.	.	.	.	.	.
N55	chr18	28909942	28909942	C	G		0.545454545	Missense_Mutation	NM_001942.3	DSG1	p.Pro154Ala	MODERA	D	D	B	B	T	PASS	7.42E-05	0	0	0.001042	0	0	0	0	.
N11	chr18	28925968	28925968	A	C		0.324324324	Missense_Mutation	NM_001942.3	DSG1	p.Glu636Ala	MODERA	D	N	B	B	T	.	.	.	.	.	.	.	.	.	.
N55	chr18	28934408	28934408	C	T		0.4375	Missense_Mutation	NM_001942.3	DSG1	p.Thr750Ile	MODERA	D	D	D	D	D	PASS	1.65E-05	0	0	0.000231	0	0	0	0	0.001818
N31	chr18	28935092	28935092	G	A		0.438356164	Missense_Mutation	NM_001942.3	DSG1	p.Gly978Asp	MODERA	N	N	D	P	T	.	.	.	.	.	.	.	.	.	.
N26	chr22	29091171	29091171	A	T		0.105263158	Missense_Mutation	NM_001005735.1	CHEK2	p.Ile483Asn	MODERA	D	D	D	D	D	.	.	.	.	.	.	.	.	.	.
N26	chr22	29091172	29091172	T	TTTCTC		0.105263158	Indel	NM_001005735.1	CHEK2	p.Ile483fs	HIGH	.	.	.	.	.	.	.	.	.	.	.	.	.	.	.
N22	chr22	29091846	29091846	G	A	COSM400	0.374193548	Splice_Site	NM_001005735.1	CHEK2	p.His414Tyr	MODERA	D	N	P	B	D	PASS	0.00048923	0	0	0.003815	0	4.61E-05	0.001272	0	0.002727
N55	chr22	29091846	29091846	G	A	COSM400	0.425	Splice_Site	NM_001005735.1	CHEK2	p.His414Tyr	MODERA	D	N	P	B	D	PASS	0.00048923	0	0	0.003815	0	4.61E-05	0.001272	0	0.002727
N36	chr22	29130702	29130702	C	T		0.437810945	Missense_Mutation	NM_001005735.1	CHEK2	p.Arg3Gln	MODERA	D	N	B	B	T	PASS	1.67E-05	0	0	0.000233	0	0	0	0	.
N28	chr17	29654830	29654830	A	G		0.188976378	Missense_Mutation	NM_001042492.2	NF1	p.Asn1861Ser	MODERA	D	D	D	D	D	.	.	.	.	.	.	.	.	.	.
N70	chr14	30108044	30108044	G	A		0.342541436	Nonsense_Mutation	NM_001330069.1	PRKD1	p.Arg263*	HIGH	A	.	.	.	.	PASS	8.24E-06	0	0	0	0	1.50E-05	0	0	.
N43	chr12	31237908	31237908	GGAA	G		0.472222222	Indel	NM_001257144.1	DDX11	p.Glu166del	MODERA	.	.	.	.	.	PASS	0.00030235	0.000185	0.000198	0.0026	0	9.65E-05	0.000197	0.002033	.
N52	chr18	31241640	31241640	C	T		0.444444444	Missense_Mutation	NM_030632.2	ASXL3	p.Thr139Ile	MODERA	D	N	P	P	D	PASS	9.15E-06	0	0	0.000126	0	0	0	0	0.000909
N75	chr12	31242060	31242060	G	A		0.586538462	Missense_Mutation	NM_001257144.1	DDX11	p.Arg256Gln	MODERA	D	N	D	P	D	PASS	2.47E-05	0	0.000259	0	0	0	0	0.003304	.
N14	chr12	31244701	31244701	G	A		0.330827068	Missense_Mutation	NM_001257144.1	DDX11	p.Ala380Thr	MODERA	D	D	P	P	D	PASS	8.48E-05	0	0	0.001172	0	0	0	0	0.000909
N52	chr2	31401445	31401445	G	C		0.104166667	Splice_Site	NM_001145122.1	CAPN14	p.Leu571Val	MODERA	N	N	P	B	T	.	.	.	.	.	.	.	.	.	.
N11	chr3	32995907	32995907	C	CT		0.209090909	Indel	NM_005508.4	CCR4	p.Cys332fs	HIGH	.	.	.	.	.	.	.	.	.	.	.	.	.	.	.
N48	chr6	33137842	33137842	C	CACA		0.128205128	Indel	NM_080680.2.6	COL11A2	p.Leu1206_Gl	MODERA	.	.	.	.	.	.	.	.	.	.	.	.	.	.	.
N48	chr6	33137845	33137845	GT	G		0.12195122	Indel	NM_080680.2.6	COL11A2	p.Asn1205fs	HIGH	.	.	.	.	.	.	.	.	.	.	.	.	.	.	.
N31	chr1	33998650	33998650	C	T		0.458333333	Splice_Site	NM_001281956.1	CSMD2	p.Glu3391Lys	MODERA	D	N	B	B	T	PASS	8.65E-06	0	0	0.000118	0	0	0	0	.
N12	chr1	34082569	34082569	C	T		0.195121951	Missense_Mutation	NM_001281956.1	CSMD2	p.Ala1985Thr	MODERA	D	N	P	B	D	PASS	6.60E-05	0	0	0.000693	0	3.00E-05	0	0	0.000455
N22	chr1	34087830	34087830	G	A		0.114155251	Missense_Mutation	NM_001281956.1	CSMD2	p.Leu1922Phe	MODERA	D	D	D	D	D	.	.	.	.	.	.	.	.	.	.
N48	chr1	34128645	34128645	C	A		0.409090909	Missense_Mutation	NM_001281956.1	CSMD2	p.Arg1367Leu	MODERA	D	D	D	P	D	.	.	.	.	.	.	.	.	.	.
N38	chr1	34166245	34166245	C	T	COSM129	0.190930788	Missense_Mutation	NM_001281956.1	CSMD2	p.Arg1202His	MODERA	D	N	D	P	T	PASS	0.00013185	0.000385	0.000173	0.000116	0	0.000105	0.000121	0.002203	.
N73	chr1	34190251	34190251	T	C		0.476470588	Missense_Mutation	NM_001281956.1	CSMD2	p.Gln917Arg	MODERA	D	N	B	B	T	PASS	2.47E-05	0	0	0.000347	0	0	0	0	0.000909
N64	chr1	34204792	34204792	C	T	COSM131	0.507293355	Missense_Mutation	NM_001281956.1	CSMD2	p.Val773Ile	MODERA	N	N	B	B	T	PASS	2.50E-05	0	8.74E-05	0.000116	0	1.51E-05	0	0.001136	.
N14	chr10	34625160	34625160	T	A	COSM438	0.517482517	Missense_Mutation	NM_019619.3	PARD3	p.Thr861Ser	MODERA	D	N	P	P	T	PASS	0.000256	0	8.70E-05	0.003471	0	0	0	0.001104	0.006364
N11	chr10	34739357	34739357	C	A		0.234042553	Missense_Mutation	NM_019619.3	PARD3	p.Ser201Ile	MODERA	D	N	D	D	T	.	.	.	.	.	.	.	.	.	.
N51	chr1	35332663	35332663	G	A		0.531914894	Missense_Mutation	NM_001080418.2	DLGAP3	p.Leu903Phe	MODERA	D	D	P	P	T	PASS	1.65E-05	0	0	0.000231	0	0	0	0	.
N68	chr1	35370171	35370171	C	T	COSM168	0.365384615	Missense_Mutation	NM_001080418.2	DLGAP3	p.Gly272Ser	MODERA	N	N	B	B	T	PASS	1.65E-05	0.000193	0	0	0	0	0	0	.
N51	chr19	35823825	35823825	C	T		0.549295775	Splice_Site	NM_001771.3	CD22	p.Ser137Phe	MODERA	D	D	D	D	D	PASS	0.00011645	0	0	0.00162	0	0	0	0	0.001364
N66	chr19	35829242	35829242	T	C	COSM319	0.558685446	Missense_Mutation	NM_001771.3	CD22	p.Ile386Thr	MODERA	N	N	B	B	D	PASS	0.00020592	0	0	0.002889	0	0	0	0	0.009545
N46	chr19	35831802	35831802	C	T		0.439688716	Missense_Mutation	NM_001771.3	CD22	p.Thr423Ile	MODERA	N	N	B	B	T	.	.	.	.	.	.	.	.	.	.
N12	chr19	35837104	35837104	C	T	COSM119	0.5	Missense_Mutation	NM_001771.3	CD22	p.Thr793Met	MODERA	N	N	P	B	T	PASS	0.00021447	9.66E-05	0	0	0.000151	0.00018	0.000728	0	.
N26	chr19	36211411	36211411	G	GCTTTTTCC		0.102564103	Indel	NM_014727.2	KMT2B	p.Glu388fs	HIGH	.	.	.	.	.	.	.	.	.	.	.	.	.	.	.
N23	chr19	36212549	36212549	A	G		0.459459459	Missense_Mutation	NM_014727.2	KMT2B	p.Gln767Arg	MODERA	N	N	B	B	T	PASS	8.18E-05	0	0	0.001139	0	0	0	0	0.000455
N76	chr19	36223712	36223712	C	T		0.477099237	Missense_Mutation	NM_014727.2	KMT2B	p.Pro2088Ser	MODERA	D	N	D	D	D	PASS	0.00014317	0	0	0.001892	0	0	0	0	0.001364
N52	chr22	36697100	36697100	T	G		0.405405405	Missense_Mutation	NM_002473.5	MYH9	p.Met879Leu	MODERA	N	N	B	B	T	PASS	0.00028467	0	0.000173	0.003499	0	0	6.07E-05	0.002247	0.006818
N23	chr4	37440569	37440569	G	A		0.123287671	Missense_Mutation	NM_001144990.1	NWD2	p.Glu285Lys	MODERA	D	N	B	B	T	.	.	.	.	.	.	.	.	.	.
N22	chr4	37445504	37445504	T	G		0.515283843	Missense_Mutation	NM_001144990.1	NWD2	p.Ser632Ala	MODERA	N	N	B	B	D	.	.	.	.	.	.	.	.	.	0.000909
N60	chr4	37445609	37445609	A	G		0.674876847	Missense_Mutation	NM_001144990.1	NWD2	p.Met667Val	MODERA	D	N	B	B	T	.	.	.	.	.	.	.	.	.	.
N68	chr4	37445826	37445826	T	G		0.195121951	Missense_Mutation	NM_001144990.1	NWD2	p.Leu739Arg	MODERA	D	N	D	D	T	.	.	.	.	.	.	.	.	.	.
N73	chr4	37446353	37446353	C	A		0.115252785	Missense_Mutation	NM_001144990.1	NWD2	p.Gln915Lys	MODERA	D	N	D	D	T	.	.	.	.	.	.	.	.	.	.
N60	chr4	37447530	37447530	C	T		0.105263158	Missense_Mutation	NM_001144990.1	NWD2	p.Thr1307Ile	MODERA	D	N	D	D	T	.	.	.	.	.	.	.	.	.	.
N60	chr4	37447532	37447532	G	T		0.106382979	Missense_Mutation	NM_001144990.1	NWD2	p.Ala1308Ser	MODERA	D	N	D	D	T	.	.	.	.	.	.	.	.	.	.
N14	chr8	38105235	38105235	C	T	COSM605	0.384615385	Missense_Mutation	NM_001164232.1	DDHD2	p.Ser377Leu	MODERA	N	N	B	B	T	PASS	0.0005096	0.000101	0.000869	0.000116	0	0.000703	6.76E-05	0.011364	0.000455
N11	chr5	38486084	38486084	T	G		0.207920792	Splice_Site	NM_001127671.1	LIFR		HIGH	D	.	.	.	.	.	.	.	.	.	.	.	.	.	.
N40	chr17	38711067	38711067	C	T	COSM570	0.236111111	Nonsense_Mutation	NM_001838.3	CCR7	p.Trp355*	HIGH	D	.	.	.	.	.	.	.	.	.	.	.	.	.	.
N70	chr17	38711460	38711460	T	C		0.482394366	Missense_Mutation	NM_001838.3	CCR7	p.Gln224Arg	MODERA	N	N	B	B	T	PASS	8.26E-06	0	0	0.000116	0	0	0	0	.
N51	chr20	39766423	39766423	C	T	COSM570	0.102272727	Missense_Mutation	NM_002660.2	PLCG1	p.Arg48Trp	MODERA	D	D	D	D	D	.	.	.	.	.	.	.	.	.	.
N28	chr20	39766463	39766463	C	T	COSM465	0.492481203	Missense_Mutation	NM_002660.2	PLCG1	p.Thr61Met	MODERA	D	N	P	B	T	PASS	0.0003268	0	0	0.004084	0	0	0	0	0.003182
N52	chr20	39792584	39792584	C	T	COSM373	0.186046512	Missense_Mutation	NM_002660.2	PLCG1	p.Ser345Phe	MODERA	D	D	D	D	D	.	.	.	.	.	.	.	.	.	.
N23	chr20	39801169	39801169	G	A	COSM215	0.548387097	Missense_Mutation	NM_002660.2	PLCG1	p.Arg1005Glr	MODERA	D	N	P	B	T	PASS	8.25E-06	0	0	0	0	1.50E-05	0	0	.
N28	chr20	39802390	39802390	G	C	COSM570	0.146496815	Missense_Mutation	NM_002660.2	PLCG1	p.Asp1165His	MODERA	D	D	D	D	D	.	.	.	.	.	.	.	.	.	.
N70	chr8	39806735	39806735	A	C		0.539923954	Missense_Mutation	NM_194294.2	IDO2	p.Glu30Asp	MODERA	N	N	B	B	T	.	.	.	.	.	.	.	.	.	.
N12	chr8	39871170	39871170	CTCAGAG	C		0.137254902	Indel	NM_194294.2	IDO2	p.Thr285_Gly	HIGH	.	.	.	.	.	.	.	.	.	.	.	.	.	.	.
N28	chr15	39883534	39883534	C	G		0.452554745	Missense_Mutation	NM_003246.3	THBS1	p.Ala799Gly	MODERA	N	N	P	B	T	.	.	.	.	.	.	.	.	.	0.000909
N70	chrX	39923086	39923086	G	A	COSM594	0.387931034	Nonsense_Mutation	NM_001123385.1	BCOR	p.Gln1208*	HIGH	A	.	.	.	.	.	.	.	.	.	.	.	.	.	.
N51	chrX	39930366	39930366	C	T		0.962962963	Missense_Mutation	NM_001123385.1	BCOR	p.Gly1033Asp	MODERA	D	N	B	B	T	.	.	.	.	.	.	.	.	.	.
N70	chrX	39931683	39931683	G	T	COSM336	0.457746479	Nonsense_Mutation	NM_001123385.1	BCOR	p.Tyr972*	HIGH	A	.	.	.	.	.	.	.	.	.	.	.	.	.	.
N60	chr17	40260078	40260078	G	T		0.5	Missense_Mutation	NM_024119.2	DHX58	p.His243Asn	MODERA	N	D	B	B	D	.	.	.	.	.	.	.	.	.	.
N51	chr17	40362212	40362212	G	C		0.119205298	Missense_Mutation	NM_012448.3	STAT5B	p.Thr628Ser	MODERA	D	D	D	D	D	PASS	8.24E-06	0	0	0	0	1.50E-05	0	0	.
N55	chr17	40462610	40462610	C	T		0.184782609	Nonsense_Mutation	NM_001288718.1	STAT5A	p.Arg770*	HIGH	D	.	.	.	.	.	.	.	.	.	.	.	.	.	.
N40	chr17	40474482	40474482	T	A	COSM115	0.407563025	Missense_Mutation	NM_139276.2	STAT3	p.Tyr640Phe	MODERA	D	D	D	D	T	PASS	1.65E-05	0	0	0	0	1.50E-05	6.06E-05	0	.

N66	chr17	40475063	40475063	T	C	COSM318	0.372881356	Missense_Mutation	NM_139276.2	STAT3	p.Glu616Gly	MODERA	D	D	P	B	D	.	.	.	.	.	.	.	.	.	.
N31	chr17	40475070	40475070	T	G	COSM406	0.638709677	Missense_Mutation	NM_139276.2	STAT3	p.Ser614Arg	MODERA	D	D	D	D	T	.	.	.	.	.	.	.	.	.	.
N64	chr2	40656553	40656553	C	T		0.297504798	Missense_Mutation	NM_021097.2	SLC8A1	p.Glu290Lys	MODERA	D	N	P	P	T	.	.	.	.	.	.	.	.	.	.
N55	chr13	41133788	41133788	TGTCACA(	T		0.136363636	Indel	NM_002015.3	FOXO1	p.Leu602_As]	MODERA	.	.	.	.	.	.	.	.	.	.	.	.	.	.	.
N36	chr13	41134396	41134396	T	C		0.487804878	Missense_Mutation	NM_002015.3	FOXO1	p.Asn411Ser	MODERA	N	N	B	B	T	PASS	1.65E-05	0	0	0.000231	0	0	0	0	.
N64	chrX	41204567	41204567	A	AG		0.681818182	Indel	NM_001356.4	DDX3X	p.Glu388fs	HIGH	.	.	.	.	.	.	.	.	.	.	.	.	.	.	.
N22	chrX	41205590	41205590	G	T		0.10738255	Splice_Site	NM_001356.4	DDX3X	p.Arg475Leu	MODERA	D	D	P	B	D	.	.	.	.	.	.	.	.	.	.
N73	chrX	41205629	41205629	G	A	COSM417	0.284210526	Missense_Mutation	NM_001356.4	DDX3X	p.Arg488His	MODERA	D	D	P	B	D	.	.	.	.	.	.	.	.	.	.
N22	chr13	41240050	41240050	C	CGCG		0.325	Indel	NM_002015.3	FOXO1	p.Ala100dup	MODERA	.	.	.	.	.	.	.	.	.	.	.	.	.	.	.
N41	chrX	41333035	41333035	C	G		0.989924433	Missense_Mutation	NM_022567.2	NYX	p.Pro110Arg	MODERA	N	D	P	B	T	.	.	.	.	.	.	.	.	.	.
N31	chr22	41533700	41533700	A	G		0.580645161	Missense_Mutation	NM_001429.3	EP300	p.Met556Val	MODERA	N	N	P	B	D	PASS	0.00012355	0	0	0.000693	0	3.00E-05	0.000424	0	0.001364
N52	chr19	41596401	41596401	A	G		0.482758621	Missense_Mutation	NM_000766.4	CYP2A13	p.Lys196Glu	MODERA	N	N	B	B	T	PASS	8.24E-06	0	0	0.000116	0	0	0	0	.
N22	chr19	41599640	41599640	G	A		0.450549451	Missense_Mutation	NM_000766.4	CYP2A13	p.Gly313Ser	MODERA	N	D	D	P	T	PASS	9.06E-05	0	0	0.001156	0	0	6.06E-05	0	0.001818
N40	chr15	41961235	41961235	G	A		0.48372093	Missense_Mutation	NM_001164273.1	MGA	p.Cys48Tyr	MODERA	N	D	D	P	D	.	.	.	.	.	.	.	.	.	.
N43	chr15	42032392	42032392	C	T		0.417475728	Missense_Mutation	NM_001164273.1	MGA	p.Arg1526Trp	MODERA	D	D	D	D	D	PASS	0.00011751	0	0.000703	0	0.000761	1.52E-05	0	0.009029	.
N22	chr15	42059392	42059392	C	T	COSM147	0.407407407	Missense_Mutation	NM_001164273.1	MGA	p.Arg3038Trp	MODERA	D	N	D	P	D	PASS	0.00035673	0	8.65E-05	0.00441	0	3.00E-05	0.000121	0.001114	0.005909
N55	chr15	42059392	42059392	C	T	COSM147	0.455882353	Missense_Mutation	NM_001164273.1	MGA	p.Arg3038Trp	MODERA	D	N	D	P	D	PASS	0.00035673	0	8.65E-05	0.00441	0	3.00E-05	0.000121	0.001114	0.005909
N14	chr18	42643449	42643449	C	A		0.5625	Missense_Mutation	NM_015559.2	SETBP1	p.Pro1526Gln	MODERA	N	N	P	P	D	.	.	.	.	.	.	.	.	.	.
N66	chr6	43164564	43164564	C	T	COSM588	0.46090535	Missense_Mutation	NM_015089.3	CUL9	p.Arg923Cys	MODERA	D	N	D	P	D	PASS	0.00022249	0.000192	0	0.001965	0	0.00012	0	0	0.002273
N73	chr6	43164564	43164564	C	T	COSM588	0.491620112	Missense_Mutation	NM_015089.3	CUL9	p.Arg923Cys	MODERA	D	N	D	P	D	PASS	0.00022249	0.000192	0	0.001965	0	0.00012	0	0	0.002273
N75	chr6	43164564	43164564	C	T	COSM588	0.699588477	Missense_Mutation	NM_015089.3	CUL9	p.Arg923Cys	MODERA	D	N	D	P	D	PASS	0.00022249	0.000192	0	0.001965	0	0.00012	0	0	0.002273
N75	chr6	43189458	43189458	G	T		0.222797927	Missense_Mutation	NM_015089.3	CUL9	p.Trp2263Leu	MODERA	D	D	D	D	D	.	.	.	.	.	.	.	.	.	.
N46	chr7	43484930	43484930	C	A		0.478225144	Missense_Mutation	NM_015052.4	HECW1	p.Ser720Tyr	MODERA	D	D	D	D	D	.	.	.	.	.	.	.	.	.	.
N46	chr7	43635607	43635607	T	G		0.419753086	Missense_Mutation	NM_004760.2	STK17A	p.Ile105Arg	MODERA	D	D	D	D	D	.	.	.	.	.	.	.	.	.	.
N26	chrX	44929432	44929432	T	TTGTATG		0.111111111	Indel	NM_001291415.1	KDM6A	p.Leu896_Ası	MODERA	.	.	.	.	.	.	.	.	.	.	.	.	.	.	.
N51	chrX	44929432	44929432	T	TTGTATG		0.103448276	Indel	NM_001291415.1	KDM6A	p.Leu896_Ası	MODERA	.	.	.	.	.	.	.	.	.	.	.	.	.	.	.
N23	chr18	45566487	45566487	T	C		0.444444444	Missense_Mutation	NM_001039360.2	ZBTB7C	p.Tyr331Cys	MODERA	D	N	D	P	D	.	.	.	.	.	.	.	.	.	.
N11	chr18	45567020	45567020	G	C	COSM287	0.11	Missense_Mutation	NM_001039360.2	ZBTB7C	p.Asp153Glu	MODERA	N	N	B	B	T	.	.	.	.	.	.	.	.	.	.
N23	chr18	45567029	45567029	A	C		0.113207547	Missense_Mutation	NM_001039360.2	ZBTB7C	p.Asp150Glu	MODERA	N	N	B	B	T	PASS	0.00033989	0.000482	8.70E-05	0.000116	0.000913	0.000392	0.000123	0.001111	.
N52	chr18	45567029	45567029	A	C		0.114754098	Missense_Mutation	NM_001039360.2	ZBTB7C	p.Asp150Glu	MODERA	N	N	B	B	T	PASS	0.00033989	0.000482	8.70E-05	0.000116	0.000913	0.000392	0.000123	0.001111	.
N60	chr18	45567029	45567029	A	C		0.141025641	Missense_Mutation	NM_001039360.2	ZBTB7C	p.Asp150Glu	MODERA	N	N	B	B	T	PASS	0.00033989	0.000482	8.70E-05	0.000116	0.000913	0.000392	0.000123	0.001111	.
N68	chr18	45567029	45567029	A	C		0.176470588	Missense_Mutation	NM_001039360.2	ZBTB7C	p.Asp150Glu	MODERA	N	N	B	B	T	PASS	0.00033989	0.000482	8.70E-05	0.000116	0.000913	0.000392	0.000123	0.001111	.
N60	chr18	45567078	45567078	C	T		0.151515152	Missense_Mutation	NM_001039360.2	ZBTB7C	p.Gly134Glu	MODERA	N	N	B	B	T	PASS	8.32E-06	0	0	0	0	1.51E-05	0	0	.
N55	chr11	45937339	45937339	C	T		0.229508197	Missense_Mutation	NM_057174.2	PEX16	p.Val92Met	MODERA	D	N	D	P	D	PASS	2.48E-05	0	0	0	0	0	0.000182	0	.
N43	chr12	46243935	46243935	G	A		0.529411765	Missense_Mutation	NM_152641.2	ARID2	p.Val677Met	MODERA	D	N	B	B	D	PASS	0.00012359	0	0	0.001738	0	0	0	0	0.007273
N23	chr3	47163727	47163727	C	T		0.416666667	Missense_Mutation	NM_014159.6	SETD2	p.Ser800Asn	MODERA	N	N	B	B	T	.	.	.	.	.	.	.	.	.	0.000909
N26	chr3	47163843	47163843	C	T	COSM158	0.487804878	Missense_Mutation	NM_014159.6	SETD2	p.Met761Ile	MODERA	N	N	B	B	T	PASS	0.00032195	0	0	0.004044	0	0	0.000242	0	0.009545
N36	chr20	47266704	47266704	T	C		0.503311258	Missense_Mutation	NM_020820.3	PREX1	p.Lys953Arg	MODERA	D	N	B	B	D	PASS	6.61E-05	0	0	0.000694	0	3.01E-05	0	0	0.005
N40	chr6	47847631	47847631	G	A	COSM216	0.251908397	Missense_Mutation	NM_001013732.3	PTCHD4	p.Arg317Trp	MODERA	D	D	P	B	D	PASS	0.00012007	0	0.001836	0.000486	0	4.71E-05	0	0.007853	0.001364
N46	chr3	48663771	48663771	C	A		0.652061856	Missense_Mutation	NM_022911.2	SLC26A6	p.Gln715His	MODERA	D	D	D	D	D	PASS	4.98E-05	0	0	0.000696	0	0	0	0	0.001364
N11	chr3	48667109	48667109	G	A	COSM122	0.504901961	Missense_Mutation	NM_022911.2	SLC26A6	p.Thr522Met	MODERA	D	D	D	D	D	PASS	1.68E-05	0	0	0.000232	0	0	0	0	.
N11	chr3	48667533	48667533	C	T		0.415929204	Missense_Mutation	NM_022911.2	SLC26A6	p.Asp462Asn	MODERA	D	D	D	D	D	PASS	2.50E-05	0	0	0.000232	0	0	6.06E-05	0	.
N22	chrX	48678628	48678628	G	C		0.185714286	Missense_Mutation	NM_001321225.1	HDAC6	p.Gly782Ala	MODERA	D	N	D	D	T	.	.	.	.	.	.	.	.	.	.
N11	chr3	49400019	49400019	G	C	COSM460	0.296	Missense_Mutation	NM_001313941.1	RHOA	p.Phe106Leu	MODERA	D	D	B	P	D	.	.	.	.	.	.	.	.	.	.
N26	chr3	49412973	49412973	C	A	COSM784	0.118421053	Missense_Mutation	NM_001313941.1	RHOA	p.Gly17Val	MODERA	D	D	D	D	D	.	.	.	.	.	.	.	.	.	.
N36	chr3	49412973	49412973	C	A	COSM784	0.183266932	Missense_Mutation	NM_001313941.1	RHOA	p.Gly17Val	MODERA	D	D	D	D	D	.	.	.	.	.	.	.	.	.	.
N46	chr22	50658411	50658411	G	A		0.499139415	Missense_Mutation	NM_020461.3	TUBGCP6	p.Ser1381Phe	MODERA	N	N	.	.	T	PASS	8.40E-06	9.83E-05	0	0	0	0	0	0	.
N76	chr22	50659237	50659237	C	T		0.465288035	Missense_Mutation	NM_020461.3	TUBGCP6	p.Arg1184Glr	MODERA	D	N	D	P	T	PASS	5.77E-05	9.63E-05	0	0	0	0	0.000363	0	.
N48	chr22	50660035	50660035	A	G		0.526315789	Missense_Mutation	NM_020461.3	TUBGCP6	p.Leu918Pro	MODERA	N	D	D	D	D	PASS	9.66E-06	0	0	0.000123	0	0	0	0	0.001364
N12	chr3	52812983	52812983	G	A		0.179487179	Missense_Mutation	NM_002215.3	ITIH1	p.Gly111Arg	MODERA	D	D	D	D	D	PASS	2.47E-05	0	0	0.000116	0	1.50E-05	6.06E-05	0	.
N70	chr3	52819084	52819084	C	T		0.544444444	Missense_Mutation	NM_002215.3	ITIH1	p.Pro478Ser	MODERA	D	D	D	D	D	PASS	6.59E-05	0	0	0.000925	0	0	0	0	0.001818
N40	chr19	55346534	55346534	G	C	COSM374	0.140350877	Splice_Site	NM_012314.5	KIR2DS4	p.Gly12Ala	MODERA	.	.	.	.	.	.	.	.	.	.	.	.	.	.	0.000455
N41	chr19	55501464	55501464	C	T	COSM397	0.239263804	Missense_Mutation	NM_001174081.1	NLRP2	p.Thr814Met	MODERA	N	N	B	B	T	PASS	1.65E-05	9.61E-05	0	0	0	1.50E-05	0	0	.
N52	chr19	55501919	55501919	G	T		0.390243902	Missense_Mutation	NM_001174081.1	NLRP2	p.Val863Leu	MODERA	N	N	B	B	T	PASS	3.29E-05	0	0	0.000116	0	3.00E-05	6.06E-05	0	0.001364
N52	chr4	55564642	55564642	G	A	COSM327	0.210526316	Splice_Site	NM_000222.2	KIT	p.Arg177His	MODERA	D	N	D	B	T	.	.	.	.	.	.	.	.	.	.
N28	chr4	55594068	55594068	G	A	COSM594	0.507246377	Missense_Mutation	NM_000222.2	KIT	p.Met618Ile	MODERA	D	N	B	B	T	PASS	2.47E-05	0	0	0.000347	0	0	0	0	0.000909
N43	chr3	57357210	57357210	G	A	COSM576	0.476923077	Missense_Mutation	NM_001291661.1	DNAH12	p.Pro2546Leu	MODERA	D	D	D	P	D	PASS	0.00051379	0	0	0	0	0.000101	0.001393	0	0.000455
N23	chr3	57368874	57368874	G	T		0.116504854	Missense_Mutation	NM_001291661.1	DNAH12	p.Leu2400Ile	MODERA	N	.	.	.	.	.	.	.	.	.	.	.	.	.	.
N52	chr3	57394145	57394145	G	T		0.506329114	Missense_Mutation	NM_001291661.1	DNAH12	p.Asn2050Lys	MODERA	N	D	D	D	D	.	.	.	.	.	.	.	.	.	.
N68	chr13	58207201	58207201	A	C		0.138888889	Missense_Mutation	NM_001040429.2	PCDH17	p.Asp174Ala	MODERA	D	D	B	B	D	.	.	.	.	.	.	.	.	.	.
N44	chr15	60297959	60297959	C	A		0.492505353	Missense_Mutation	NM_012182.2	FOXB1	p.Thr266Lys	MODERA	D	D	P	B	D	.	.	.	.	.	.	.	.	.	.
N69	chr3	61989047	61989047	A	G		0.467213115	Missense_Mutation	NM_002841.3	PTPRG	p.Tyr132Cys	MODERA	D	D	D	D	D	PASS	2.48E-05	0	0	0	0	3.00E-05	6.08E-05	0	.
N41	chr3	62189247	62189247	G	A		0.185714286	Missense_Mutation	NM_002841.3	PTPRG	p.Arg593Gln	MODERA	D	N	P	B	D	PASS	4.59E-05	0	0	0	0	0	0.000126	0	.
N40	chr11	64428354	64428354	A	C		0.485294118	Missense_Mutation	NM_015080.3	NRXN2	p.Cys686Gly	MODERA	D	D	D	D	D	.	.	.	.	.	.	.	.	.	.
N64	chr11	64480571	64480571	T	C		0.451923077	Missense_Mutation	NM_015080.3	NRXN2	p.Thr201Ala	MODERA	N	N	B	B	T	.	.	.	.	.	.	.	.	.	0.000455
N64	chrX	64949420	64949420	C	T		0.663636364	Nonsense_Mutation	NM_002444.2	MSN	p.Gln105*	HIGH	A	.	.	.	.	.	.	.	.	.	.	.	.	.	.
N76	chrX	64950957	64950957	TTGTCTTT	T		0.131313131	Indel	NM_002444.2	MSN	p.Val157fs	HIGH	.	.	.	.	.	.	.	.	.	.	.	.	.	.	.
N76	chrX	64955137	64955137	CT	C		0.258064516	Indel	NM_002444.2	MSN	p.Phe269fs	HIGH	.	.	.	.	.	.	.	.	.	.	.	.	.	.	.
N66	chrX	64959000	64959000	G	T		0.286384977	Nonsense_Mutation	NM_002444.2	MSN	p.Glu505*	HIGH	A	.	.	.	.	.	.	.	.	.	.	.	.	.	.
N11	chr3	65342238	65342238	T	A		0.27826087	Missense_Mutation	NM_001033057.1	MAGI1	p.Arg1402Trp	MODERA	D	N	D	P	D	.	.	.	.	.	.	.	.	.	.
N40	chr3	65365010	65365010	C	T		0.345498783	Missense_Mutation	NM_001033057.1	MAGI1	p.Arg974Gln	MODERA	D	.	.	.	.	PASS	0.00386386	0.000109	0	0.002823	0.024781	0.003669	0.001095	0	0.007727
N38	chr5	67576362	67576362	A	T		0.506493506	Missense_Mutation	NM_181523.2	PIK3R1	p.Gln214Leu	MODERA	D	N	B	B	T	PASS	8.30E-06	0	0	0.000116	0	0	0	0	0.000455
N55	chr5	67588117	67588117	C	CATTAGAACTAAA		0.125	Indel	NM_181523.2	PIK3R1	p.Val317fs	HIGH	.	.	.	.	.	.	.	.	.	.	.	.	.	.	.
N55	chr16	70363321	70363321	A	C		0.193548387	Splice_Site	NM_007242.5	DDX19B	p.Asp245Ala	MODERA	D	D	D	D	D	.	.	.	.	.	.	.	.	.	.
N55	chr16	70363322	70363322	T	A		0.1875	Splice_Site	NM_007242.5	DDX19B	p.Asp245Glu	MODERA	D	D	D	D	D	.	.	.	.	.	.	.	.	.	.
N46	chr10	70404762	70404762	G	C		0.130044843	Missense_Mutation	NM_030625.2	TET1	p.Gly759Ala	MODERA	D	N	D	D	D	.	.	.	.	.	.	.	.	.	.
N40	chr10	70405010	70405010	A	G		0.513513514	Missense_Mutation	NM_030625.2	TET1	p.Ile842Val	MODERA	N	N	B	B	T	PASS	0.00024728	0	0	0.003467	0	0	0	0	0.006364
N68	chr10	70405010	70405010	A	G		0.285714286	Missense_Mutation	NM_030625.2	TET1	p.Ile842Val	MODERA	N	N	B	B	T	PASS	0.00024728	0	0	0.003467	0	0	0	0	0.006364
N70	chr10	70412305	70412305	C	A		0.205426357	Missense_Mutation	NM_030625.2	TET1	p.Thr1472Asr	MODERA	D	D	D	D	D	PASS	8.27E-06	0	0	0	0	1.50E-05	0	0	.

N44	chr8	71069365	71069365	T	G		0.437086093	Missense_Mutation	NM_001321703.1	NCOA2	p.Asn412Thr	MODERA	D	N	B	B	T	PASS	0.00061088	0	0	0.007683	0	0	0.000445	0	0.004091
N11	chr8	71075034	71075034	C	T		0.180722892	Nonsense_Mutation	NM_001321703.1	NCOA2	p.Trp296*	HIGH	A	.	.	.	.	.	.	.	.	.	.	.	.	.	.
N41	chr17	71282314	71282314	G	A		0.190751445	Missense_Mutation	NM_012121.4	CDC42EP	p.Ser109Leu	MODERA	D	N	D	D	T	PASS	1.66E-05	9.71E-05	0	0	0	1.51E-05	0	0	.
N28	chr4	71390617	71390617	G	A		0.449438202	Missense_Mutation	NM_212557.3	AMTN	p.Gly78Asp	MODERA	N	D	D	D	D	PASS	9.10E-05	0	0	0.001282	0	0	0	0	0.002273
N66	chr1	74819792	74819792	T	A		0.498039216	Missense_Mutation	NM_001112808.2	FPGT-TNI	p.Leu500Met	MODERA	.	.	.	.	.	.	.	.	.	.	.	.	.	.	.
N68	chr17	76212102	76212102	G	A		0.101123596	Missense_Mutation	NM_001012271.1	BIRC5	p.Gly93Arg	MODERA	N	N	P	B	T	PASS	9.77E-06	0.00011	0	0	0	0	0	0	.
N26	chr17	76212105	76212105	C	T	COSM338	0.141509434	Missense_Mutation	NM_001012271.1	BIRC5	p.Arg94Trp	MODERA	N	N	P	B	D	PASS	2.97E-05	0	0	0	0	5.17E-05	0	0	.
N48	chr17	76212112	76212112	C	CCT		0.134615385	Indel	NM_001012271.1	BIRC5	p.Arg97fs	HIGH	.	.	.	.	.	.	.	.	.	.	.	.	.	.	.
N73	chr10	76719805	76719805	ACTC	A		0.129777778	Indel	NM_012330.3	KAT6B	p.Leu235del	MODERA	.	.	.	.	.	.	.	.	.	.	.	.	.	.	.
N23	chr10	76781848	76781848	C	G		0.107142857	Missense_Mutation	NM_012330.3	KAT6B	p.Asp1077Glu	MODERA	N	N	B	B	T	.	.	.	.	.	.	.	.	.	.
N41	chr10	76789083	76789083	G	A		0.505952381	Missense_Mutation	NM_012330.3	KAT6B	p.Glu1501Lys	MODERA	D	N	P	B	D	.	.	.	.	.	.	.	.	.	.
N52	chr10	76789083	76789083	G	A		0.348214286	Missense_Mutation	NM_012330.3	KAT6B	p.Glu1501Lys	MODERA	D	N	P	B	D	.	.	.	.	.	.	.	.	.	.
N23	chr3	77530253	77530253	C	T		0.2	Missense_Mutation	NM_001290040.1	ROBO2	p.Arg184Cys	MODERA	.	D	D	D	D	PASS	8.30E-06	0	8.69E-05	0	0	0	0	0.001111	.
N68	chr8	77618720	77618720	GCAGAAC	G		0.113636364	Indel	NM_024721.4	ZFHX4	p.Met802_As	MODERA	.	.	.	.	.	.	.	.	.	.	.	.	.	.	.
N11	chr3	77684174	77684174	G	A	COSM122	0.603174603	Missense_Mutation	NM_001290040.1	ROBO2	p.Arg1370Gln	MODERA	.	N	D	P	T	PASS	0.00037469	0.000103	0	0.004787	0	4.52E-05	0	0	0.001364
N46	chr3	77684174	77684174	G	A	COSM122	0.264957265	Missense_Mutation	NM_001290040.1	ROBO2	p.Arg1370Gln	MODERA	.	N	D	P	T	PASS	0.00037469	0.000103	0	0.004787	0	4.52E-05	0	0	0.001364
N73	chr8	77754941	77754941	G	C		0.502173913	Missense_Mutation	NM_024721.4	ZFHX4	p.Glu1149Gln	MODERA	N	N	B	B	T	PASS	3.99E-05	0	0	0.001462	0	0	0	0	0.000909
N51	chr8	77766298	77766298	G	A		0.436781609	Missense_Mutation	NM_024721.4	ZFHX4	p.Ala2381Thr	MODERA	D	N	B	B	D	PASS	0.00012431	0	0	0.001631	0	0	6.06E-05	0	0.000909
N55	chr8	77775646	77775646	T	TTGCTTTTTCTCA		0.15942029	Indel	NM_024721.4	ZFHX4	p.Val3232_V	MODERA	.	.	.	.	.	.	.	.	.	.	.	.	.	.	.
N36	chr7	77789464	77789464	G	T		0.474576271	Missense_Mutation	NM_012301.3	MAGI2	p.Pro908His	MODERA	D	N	D	P	T	PASS	9.92E-05	0	0	0.00104	0	4.51E-05	0	0	0.002727
N43	chr17	78059859	78059859	G	A	COSM453	0.391666667	Missense_Mutation	NM_017950.3	CCDC40	p.Glu765Lys	MODERA	D	D	D	P	T	PASS	0.00016608	0	0	0.002204	0	1.50E-05	0	0	0.005
N64	chr17	78064005	78064005	C	T		0.598784195	Missense_Mutation	NM_001243342.1	CCDC40	p.Thr967Met	MODERA	N	N	.	.	D	.	.	.	.	.	.	.	.	.	.
N68	chr17	78064139	78064139	C	CGAA		0.263157895	Indel	NM_001243342.1	CCDC40	p.Arg1012dup	MODERA	.	.	.	.	.	.	.	.	.	.	.	.	.	.	.
N51	chr6	79655029	79655029	C	T		0.461538462	Missense_Mutation	NM_017934.5	PHIP	p.Val1606Ile	MODERA	N	N	B	B	T	PASS	1.65E-05	0	0	0.000231	0	0	0	0	.
N76	chr11	82443648	82443648	G	C		0.451467269	Missense_Mutation	NM_175885.3	FAM181B	p.Pro375Arg	MODERA	N	N	P	P	D	PASS	2.02E-05	0	0	0.000247	0	0	0	0	0.000455
N28	chr1	86304317	86304317	G	A		0.438596491	Missense_Mutation	NM_152890.5	COL24A1	p.Pro1211Leu	MODERA	.	.	.	.	.	PASS	0.00026524	0.000204	0	0.003257	0	3.00E-05	0	0	0.008182
N38	chr1	86426944	86426944	T	G		0.566091954	Missense_Mutation	NM_152890.5	COL24A1	p.Lys850Asn	MODERA	.	.	.	.	.	PASS	4.99E-05	0	0	0.000697	0	0	0	0	0.000909
N60	chr1	86590929	86590929	T	A		0.157575758	Nonsense_Mutation	NM_152890.5	COL24A1	p.Lys364*	HIGH	.	.	.	.	.	.	.	.	.	.	.	.	.	.	.
N11	chr1	86590983	86590983	T	A		0.205357143	Missense_Mutation	NM_152890.5	COL24A1	p.Asn346Tyr	MODERA	.	.	.	.	.	.	.	.	.	.	.	.	.	.	0.000455
N68	chr1	86591332	86591332	T	G	COSM441	0.2	Missense_Mutation	NM_152890.5	COL24A1	p.Glu229Asp	MODERA	.	.	.	.	.	.	.	.	.	.	.	.	.	.	.
N31	chr16	87921784	87921784	C	T		0.529411765	Missense_Mutation	NM_001739.1	CA5A	p.Arg290Gln	MODERA	D	D	D	D	D	PASS	5.77E-05	0	8.64E-05	0.000116	0	1.50E-05	0.000182	0.001104	0.000909
N48	chr15	88472435	88472435	G	A	COSM201	0.53968254	Splice_Site	NM_001012338.2	NTRK3	p.Thr707Met	MODERA	D	D	D	P	D	PASS	3.30E-05	0	0	0.000231	0	0	0.000121	0	.
N36	chr4	88957405	88957405	A	G		0.525	Missense_Mutation	NM_000297.3	PKD2	p.Tyr248Cys	MODERA	D	D	D	D	D	PASS	9.11E-05	9.76E-05	0	0.00116	0	0	0	0	0.001818
N75	chr4	88957405	88957405	A	G		0.41509434	Missense_Mutation	NM_000297.3	PKD2	p.Tyr248Cys	MODERA	D	D	D	D	D	PASS	9.11E-05	9.76E-05	0	0.00116	0	0	0	0	0.001818
N48	chr15	90176198	90176198	G	C		0.515151515	Missense_Mutation	NM_198525.2	KIF7	p.Asp916Glu	MODERA	D	D	B	B	D	PASS	8.54E-06	0	0	0.000118	0	0	0	0	.
N70	chr15	90185484	90185484	G	A	COSM434	0.606965174	Missense_Mutation	NM_198525.2	KIF7	p.Arg782Trp	MODERA	N	D	D	P	D	PASS	1.74E-05	0	0	0	0	3.17E-05	0	0	.
N36	chr15	90631837	90631837	C	G	COSM133	0.210843373	Missense_Mutation	NM_002168.3	IDH2	p.Arg172Ser	MODERA	D	D	D	D	D	.	.	.	.	.	.	.	.	.	.
N73	chr15	90631929	90631929	T	G	COSM594	0.481124498	Missense_Mutation	NM_002168.3	IDH2	p.Ile142Leu	MODERA	D	N	P	P	D	PASS	8.24E-06	0	0	0.000116	0	0	0	0	0.000909
N11	chr10	90771840	90771840	T	C		0.3	Splice_Site	NM_000043.5	FAS		HIGH	D	.	.	.	.	.	.	.	.	.	.	.	.	.	.
N64	chr10	90773124	90773124	G	T		0.234899329	Nonsense_Mutation	NM_001320619.1	FAS	p.Ter198Leue	HIGH	D	D	D	D	D	.	.	.	.	.	.	.	.	.	.
N40	chr10	94821816	94821816	A	G		0.50728863	Missense_Mutation	NM_183374.2	CYP26C1	p.Lys85Arg	MODERA	D	N	P	D	T	.	.	.	.	.	.	.	.	.	.
N48	chr10	94828149	94828149	C	A		0.403508772	Missense_Mutation	NM_183374.2	CYP26C1	p.Arg422Ser	MODERA	N	N	B	B	T	PASS	3.63E-05	0	0	0.000358	0	0	0	0	0.000909
N36	chr7	94898699	94898699	G	T		0.525252525	Missense_Mutation	NM_001166160.1	PPP1R9A	p.Gly1002Trp	MODERA	D	N	P	B	D	PASS	6.80E-05	0	0	0.000964	0	0	0	0	0.003182
N36	chr8	95871760	95871760	T	C		0.484615385	Missense_Mutation	NM_017864.3	INTS8	p.Ile659Thr	MODERA	D	D	B	B	D	.	.	.	.	.	.	.	.	.	.
N70	chr14	99640672	99640672	G	A	COSM137	0.328621908	Missense_Mutation	NM_138576.3	BCL11B	p.Ala834Val	MODERA	D	D	D	D	D	.	.	.	.	.	.	.	.	.	.
N41	chr14	99641990	99641990	A	T		0.277777778	Missense_Mutation	NM_138576.3	BCL11B	p.Phe395Ile	MODERA	D	D	D	D	D	.	.	.	.	.	.	.	.	.	.
N26	chr9	100194348	100194348	A	G		0.527027027	Missense_Mutation	NM_014290.2	TDRD7	p.Asn131Asp	MODERA	N	N	B	B	T	PASS	0.00041224	0	0	0.00578	0	0	0	0	0.008636
N43	chr9	100194348	100194348	A	G		0.483146067	Missense_Mutation	NM_014290.2	TDRD7	p.Asn131Asp	MODERA	N	N	B	B	T	PASS	0.00041224	0	0	0.00578	0	0	0	0	0.008636
N22	chr9	100240748	100240748	C	T	COSM118	0.434042553	Missense_Mutation	NM_014290.2	TDRD7	p.Leu732Phe	MODERA	D	D	D	D	D	PASS	3.30E-05	0	0	0.000116	0	0	0.000182	0	.
N70	chr9	100245445	100245445	G	T		0.178423237	Missense_Mutation	NM_014290.2	TDRD7	p.Lys909Asn	MODERA	N	N	B	B	T	.	.	.	.	.	.	.	.	.	.
N73	chr15	101529581	101529581	C	A		0.48696845	Missense_Mutation	NM_024652.4	LRRK1	p.Pro247His	MODERA	N	N	B	B	D	.	.	.	.	.	.	.	.	.	.
N66	chr15	101593206	101593206	A	G	COSM989	0.497524752	Missense_Mutation	NM_024652.4	LRRK1	p.Ile1257Val	MODERA	D	N	D	P	T	PASS	2.52E-05	0	0	0.000351	0	0	0	0	0.002273
N31	chr8	101716537	101716537	G	A		0.290456432	Missense_Mutation	NM_002568.3	PABPC1	p.Pro634Ser	MODERA	D	N	B	B	D	.	.	.	.	.	.	.	.	.	.
N69	chr8	101719224	101719224	T	TGGATGAGGTCTG		0.102515723	Indel	NM_002568.3	PABPC1	p.Pro446_Phe	MODERA	.	.	.	.	.	.	.	.	.	.	.	.	.	.	.
N64	chr4	103537684	103537684	C	T	COSM442	0.43358396	Missense_Mutation	NM_003998.3	NFKB1	p.Ser948Leu	MODERA	N	N	B	B	D	PASS	8.24E-06	0	0	0	0	0	6.06E-05	0	.
N38	chr14	105243058	105243058	G	C		0.578849722	Missense_Mutation	NM_001014431.1	AKT1	p.Ile75Met	MODERA	D	N	D	D	D	PASS	1.66E-05	0	0	0.000232	0	0	0	0	0.001364
N68	chr4	106155299	106155299	A	C		0.163265306	Missense_Mutation	NM_001127208.2	TET2	p.Lys67Thr	MODERA	N	N	D	P	D	.	.	.	.	.	.	.	.	.	.
N66	chr4	106157521	106157521	G	A		0.452282158	Missense_Mutation	NM_001127208.2	TET2	p.Glu808Lys	MODERA	N	N	B	B	T	.	.	.	.	.	.	.	.	.	.
N40	chr4	106157548	106157548	T	TC		0.345454545	Indel	NM_001127208.2	TET2	p.Tyr819fs	HIGH	.	.	.	.	.	.	.	.	.	.	.	.	.	.	.
N51	chr4	106164085	106164085	G	T	COSM438	0.168	Splice_Site	NM_001127208.2	TET2		HIGH	D	.	.	.	.	.	.	.	.	.	.	.	.	.	.
N14	chr4	106180796	106180796	G	T		0.147286822	Missense_Mutation	NM_001127208.2	TET2	p.Gly1275Val	MODERA	D	D	D	D	D	.	.	.	.	.	.	.	.	.	.
N48	chr4	106180838	106180838	G	A	COSM871	0.282352941	Missense_Mutation	NM_001127208.2	TET2	p.Cys1289Tyr	MODERA	D	D	D	D	D	.	.	.	.	.	.	.	.	.	.
N43	chr4	106180841	106180841	C	G		0.121212121	Nonsense_Mutation	NM_001127208.2	TET2	p.Ser1290*	HIGH	A	.	.	.	.	.	.	.	.	.	.	.	.	.	.
N11	chr4	106180895	106180895	A	C		0.265957447	Splice_Site	NM_001127208.2	TET2	p.Lys1308Thr	MODERA	D	D	D	D	D	.	.	.	.	.	.	.	.	.	.
N44	chr4	106190830	106190830	G	A	COSM438	0.16374269	Splice_Site	NM_001127208.2	TET2	p.Gly1370Arg	MODERA	D	D	D	D	D	.	.	.	.	.	.	.	.	.	.
N38	chr4	106190836	106190836	A	C		0.342592593	Splice_Site	NM_001127208.2	TET2	p.Thr1372Pro	MODERA	D	D	D	D	D	.	.	.	.	.	.	.	.	.	.
N51	chr4	106190854	106190854	T	C		0.283185841	Splice_Site	NM_001127208.2	TET2	p.Cys1378Arg	MODERA	D	D	D	D	D	.	.	.	.	.	.	.	.	.	.
N41	chr4	106190861	106190861	A	G	COSM227	0.160583942	Splice_Site	NM_001127208.2	TET2	p.His1380Arg	MODERA	D	D	D	D	D	.	.	.	.	.	.	.	.	.	0.000455
N36	chr4	106194063	106194063	A	T	COSM594	0.192982456	Nonsense_Mutation	NM_001127208.2	TET2	p.Lys1509*	HIGH	D	.	.	.	.	.	.	.	.	.	.	.	.	.	.
N36	chr4	106196345	106196345	T	TA	COSM171	0.237288136	Indel	NM_001127208.2	TET2	p.Tyr1560fs	HIGH	.	.	.	.	.	.	.	.	.	.	.	.	.	.	.
N69	chr4	106196349	106196349	C	T		0.211890244	Missense_Mutation	NM_001127208.2	TET2	p.Ser1561Phe	MODERA	N	D	P	B	D	.	.	.	.	.	.	.	.	.	.
N41	chr4	106197242	106197242	AT	A		0.285171103	Indel	NM_001127208.2	TET2	p.Ile1859fs	HIGH	.	.	.	.	.	.	.	.	.	.	.	.	.	.	.
N44	chr7	106508095	106508095	C	G		0.41106383	Missense_Mutation	NM_001282426.1	PIK3CG	p.Ala30Gly	MODERA	N	N	B	B	T	PASS	0.00011921	0	0	0.001406	0	0	0.000123	0	0.000455
N26	chr7	106509049	106509049	G	C		0.458333333	Missense_Mutation	NM_001282426.1	PIK3CG	p.Ser348Thr	MODERA	N	N	B	B	T	.	.	.	.	.	.	.	.	.	.
N46	chr6	106536269	106536269	C	CGG		0.408088235	Indel	NM_001198.3	PRDM1	p.Glu80fs	HIGH	.	.	.	.	.	.	.	.	.	.	.	.	.	.	.
N46	chr6	106553748	106553748	CAAGATC	C		0.288273616	Indel	NM_001198.3	PRDM1	p.Lys572fs	HIGH	.	.	.	.	.	.	.	.	.	.	.	.	.	.	.
N14	chr6	106554373	106554373	A	G		0.4140625	Splice_Site	NM_001198.3	PRDM1	p.Gln634Arg	MODERA	D	N	B	B	D	PASS	0.00012391	0	0	0.001625	0	1.50E-05	0	0	0.004545
N52	chr6	106727593	106727593	C	T		0.443181818	Splice_Site	NM_001286106.1	ATG5	p.Val141Ile	MODERA	D	N	P	P	T	PASS	8.28E-06	0	0	0.000116	0	0	0	0	0.000455
N66	chrX	106840700	106840700	C	T		0.869791667	Missense_Mutation	NM_032428.1	FRMPD3	p.Leu564Phe	MODERA	N	N	.	.	D	PASS	8.52E-05	0	0	0.001081	0	0	0	0	0.000455

N11	chrX	106845626	106845626	T	C		0.111111111	Missense_Mutation	NM_032428.1	FRMPD3	p.Tyr1486His	MODERA	N	N	.	.	T	.	.	.	.	.	.	.	.	.	.
N11	chrX	106845627	106845627	A	AG		0.111111111	Indel	NM_032428.1	FRMPD3	p.Tyr1486fs	HIGH	.	.	.	.	.	.	.	.	.	.	.	.	.	.	.
N11	chrX	106845631	106845631	CCA	C		0.105263158	Indel	NM_032428.1	FRMPD3	p.Gln1488fs	HIGH	.	.	.	.	.	.	.	.	.	.	.	.	.	.	.
N22	chr6	106968078	106968078	G	A		0.533834586	Missense_Mutation	NM_001624.3	AIM1	p.Val591Ile	MODERA	N	N	P	B	D	PASS	9.07E-05	0.000192	8.66E-05	0.000578	0	1.50E-05	0.000121	0.001104	0.002727
N66	chr6	106968078	106968078	G	A		0.213836478	Missense_Mutation	NM_001624.3	AIM1	p.Val591Ile	MODERA	N	N	P	B	D	PASS	9.07E-05	0.000192	8.66E-05	0.000578	0	1.50E-05	0.000121	0.001104	0.002727
N70	chr6	106968078	106968078	G	A		0.29739777	Missense_Mutation	NM_001624.3	AIM1	p.Val591Ile	MODERA	N	N	P	B	D	PASS	9.07E-05	0.000192	8.66E-05	0.000578	0	1.50E-05	0.000121	0.001104	0.002727
N46	chr6	106992685	106992685	T	G		0.519417476	Missense_Mutation	NM_001624.3	AIM1	p.Ile1322Arg	MODERA	D	D	B	B	T	PASS	1.66E-05	0	0	0.000233	0	0	0	0	0.000455
N64	chr7	107799939	107799939	T	G		0.111111111	Missense_Mutation	NM_001037132.2	NRCAM	p.Lys1216Gln	MODERA	D	D	P	B	D	.	.	.	.	.	.	.	.	.	.
N48	chr11	108119802	108119802	C	T		0.486486486	Missense_Mutation	NM_000051.3	ATM	p.Ser403Leu	MODERA	N	N	B	B	T	PASS	8.26E-06	0	0	0.000116	0	0	0	0	0.000455
N60	chr11	108119802	108119802	C	T		0.543209877	Missense_Mutation	NM_000051.3	ATM	p.Ser403Leu	MODERA	N	N	B	B	T	PASS	8.26E-06	0	0	0.000116	0	0	0	0	0.000455
N38	chr11	108122760	108122760	T	C		0.393939394	Splice_Site	NM_000051.3	ATM		HIGH	D	.	.	.	.	.	.	.	.	.	.	.	.	.	.
N51	chr11	108139269	108139269	G	A		0.504587156	Missense_Mutation	NM_000051.3	ATM	p.Arg924Gln	MODERA	D	N	P	B	T	PASS	9.88E-05	0	0	0.000578	0	0	0.000424	0	0.001818
N38	chr11	108159769	108159769	A	AT		0.208333333	Indel	NM_000051.3	ATM	p.Ile1393fs	HIGH	.	.	.	.	.	.	.	.	.	.	.	.	.	.	.
N70	chr13	110859057	110859057	C	A		0.169312169	Missense_Mutation	NM_001845.5	COL4A1	p.Met271Ile	MODERA	N	N	B	B	T	.	.	.	.	.	.	.	.	.	.
N55	chr12	111885509	111885509	T	C		0.142857143	Missense_Mutation	NM_005475.2	SH2B3	p.Leu429Pro	MODERA	D	D	D	P	D	.	.	.	.	.	.	.	.	.	.
N11	chr6	111982968	111982968	G	A	COSM570	0.256637168	Nonsense_Mutation	NM_002037.5	FYN	p.Gln530*	HIGH	D	.	.	.	.	.	.	.	.	.	.	.	.	.	.
N40	chr6	112024238	112024238	C	T		0.307317073	Splice_Site	NM_002037.5	FYN		HIGH	D	.	.	.	.	.	.	.	.	.	.	.	.	.	.
N28	chr9	112082485	112082485	G	A		0.455882353	Missense_Mutation	NM_019114.4	EPB41L4E	p.Ala81Val	MODERA	N	N	B	B	T	.	.	.	.	.	.	.	.	.	.
N68	chr5	112103035	112103035	G	C		0.365079365	Missense_Mutation	NM_000038.5	APC	p.Val124Leu	MODERA	N	N	B	B	T	PASS	8.24E-06	0	0	0.000116	0	0	0	0	.
N52	chr6	112671328	112671328	T	C		0.555555556	Missense_Mutation	NM_001013734.2	RFPL4B	p.Cys140Arg	MODERA	N	N	B	B	T	PASS	8.25E-06	0	0	0.000116	0	0	0	0	.
N70	chr8	113256668	113256668	A	C		0.379591837	Missense_Mutation	NM_198123.1	CSMD3	p.Cys3453Gly	MODERA	D	D	D	D	D	.	.	.	.	.	.	.	.	.	.
N52	chr8	113332159	113332159	AT	A		0.173913043	Indel	NM_198123.1	CSMD3	p.Ile2406fs	HIGH	.	.	.	.	.	.	.	.	.	.	.	.	.	.	.
N43	chr8	113349030	113349030	C	A		0.430769231	Missense_Mutation	NM_198123.1	CSMD3	p.Met2290Ile	MODERA	D	N	B	B	T	.	.	.	.	.	.	.	.	.	0.000455
N28	chr8	113358435	113358435	A	T		0.131944444	Nonsense_Mutation	NM_198123.1	CSMD3	p.Cys2111*	HIGH	A	.	.	.	.	.	.	.	.	.	.	.	.	.	.
N22	chr8	113418798	113418798	G	A		0.35042735	Missense_Mutation	NM_198123.1	CSMD3	p.Pro1922Ser	MODERA	D	D	D	D	D	.	.	.	.	.	.	.	.	.	.
N11	chr8	113418945	113418945	T	G		0.425925926	Missense_Mutation	NM_198123.1	CSMD3	p.Thr1873Pro	MODERA	D	D	D	P	D	.	.	.	.	.	.	.	.	.	.
N60	chr8	113420600	113420600	C	T		0.136986301	Missense_Mutation	NM_198123.1	CSMD3	p.Arg1851Glr	MODERA	D	D	D	B	T	PASS	0.00010716	0	0.00104	0	0	1.50E-05	0	0.013274	.
N23	chr8	113662432	113662432	C	T	COSM136	0.153846154	Missense_Mutation	NM_198123.1	CSMD3	p.Glu1051Lys	MODERA	D	D	D	P	D	PASS	8.24E-06	0	8.64E-05	0	0	0	0	0.001101	.
N23	chr8	113678533	113678533	C	T	COSM454	0.192982456	Missense_Mutation	NM_198123.1	CSMD3	p.Gly930Glu	MODERA	D	D	D	D	D	.	.	.	.	.	.	.	.	.	.
N73	chr8	113702147	113702147	A	G		0.496392496	Missense_Mutation	NM_198123.1	CSMD3	p.Ile702Thr	MODERA	D	D	D	D	D	.	.	.	.	.	.	.	.	.	.
N60	chr1	117064531	117064531	T	TAG		0.107692308	Indel	NM_001779.2	CD58	p.Asn235fs	HIGH	.	.	.	.	.	.	.	.	.	.	.	.	.	.	.
N60	chr1	117064533	117064533	A	AG		0.101449275	Indel	NM_001779.2	CD58	p.Met234fs	HIGH	.	.	.	.	.	.	.	.	.	.	.	.	.	.	.
N60	chr1	117064561	117064561	C	G		0.111111111	Missense_Mutation	NM_001779.2	CD58	p.Val225Leu	MODERA	.	.	.	.	.	.	.	.	.	.	.	.	.	.	.
N69	chr1	117078622	117078622	G	C		0.350847458	Nonsense_Mutation	NM_001779.2	CD58	p.Ser198*	HIGH	.	.	.	.	.	.	.	.	.	.	.	.	.	.	.
N23	chr11	117265691	117265691	A	ATCT		0.125	Indel	NM_014956.4	CEP164	p.Lys939delin	MODERA	.	.	.	.	.	.	.	.	.	.	.	.	.	.	.
N23	chr11	117265695	117265695	CAGA	C		0.135135135	Indel	NM_014956.4	CEP164	p.Arg941del	MODERA	.	.	.	.	.	.	.	.	.	.	.	.	.	.	.
N23	chr11	117265701	117265701	G	GACATCTTTA		0.142857143	Indel	NM_014956.4	CEP164	p.Leu942_Ala	MODERA	.	.	.	.	.	.	.	.	.	.	.	.	.	.	.
N22	chr11	117779492	117779492	C	T		0.486772487	Missense_Mutation	NM_001077263.2	TMPRSS1	p.Glu373Lys	MODERA	D	N	D	P	T	.	.	.	.	.	.	.	.	.	.
N76	chr11	118307391	118307391	C	A		0.494897959	Missense_Mutation	NM_001197104.1	KMT2A	p.Pro55His	MODERA	N	N	B	B	D	.	.	.	.	.	.	.	.	.	.
N48	chr11	118392008	118392008	C	T		0.492307692	Missense_Mutation	NM_001197104.1	KMT2A	p.Pro3840Leu	MODERA	D	D	D	B	D	PASS	8.24E-06	0	0	0.000116	0	0	0	0	.
N43	chr10	120803610	120803610	T	TGTTAATATCTTAT		0.148648649	Indel	NM_003750.2	EIF3A	p.Lys901fs	HIGH	.	.	.	.	.	.	.	.	.	.	.	.	.	.	.
N48	chr10	120803610	120803610	T	TGTTAATATCTTAT		0.110429448	Indel	NM_003750.2	EIF3A	p.Lys901fs	HIGH	.	.	.	.	.	.	.	.	.	.	.	.	.	.	.
N23	chr10	120803610	120803610	T	TGTTAATATCTTA		0.111111111	Indel	NM_003750.2	EIF3A	p.Lys901delin	MODERA	.	.	.	.	.	.	.	.	.	.	.	.	.	.	.
N69	chr10	120818770	120818770	G	T		0.313787639	Nonsense_Mutation	NM_003750.2	EIF3A	p.Ser528*	HIGH	A	.	.	.	.	.	.	.	.	.	.	.	.	.	.
N75	chr10	120833042	120833042	C	T		0.545977011	Missense_Mutation	NM_003750.2	EIF3A	p.Met96Ile	MODERA	N	N	B	B	D	PASS	0.00033862	0	0	0.004743	0	0	0	0	0.001818
N11	chrX	122319682	122319682	A	C		0.46	Splice_Site	NM_000828.4	GRIA3		HIGH	D	.	.	.	.	.	.	.	.	.	.	.	.	.	.
N38	chr5	127595389	127595389	G	A	COSM143	0.146596859	Missense_Mutation	NM_001999.3	FBN2	p.Arg2833Cys	MODERA	D	D	D	P	D	.	.	.	.	.	.	.	.	.	.
N52	chr5	127625639	127625639	A	G		0.474576271	Splice_Site	NM_001999.3	FBN2	p.Val2149Ala	MODERA	D	N	B	B	T	PASS	0.00015295	0	0	0.00209	0	0	0	0	0.005
N36	chr5	127782294	127782294	C	T		0.562211982	Missense_Mutation	NM_001999.3	FBN2	p.Asp278Asn	MODERA	D	D	D	D	T	.	.	.	.	.	.	.	.	.	.
N28	chrX	129147386	129147386	T	A		0.163157895	Missense_Mutation	NM_001184772.2	BCORL1	p.Val213Glu	MODERA	N	N	P	B	T	.	.	.	.	.	.	.	.	.	.
N28	chr2	133489315	133489315	G	A	COSM356	0.330275229	Missense_Mutation	NM_207363.2	NCKAP5	p.Ser1813Phe	MODERA	N	D	D	P	D	PASS	3.53E-05	0.000212	0	0.000117	0	1.55E-05	0	0	.
N64	chr9	133911614	133911614	C	T		0.567385445	Missense_Mutation	NM_006059.3	LAMC3	p.Arg291Trp	MODERA	N	D	D	D	T	PASS	4.28E-05	0	8.71E-05	0.000118	0	3.14E-05	0	0.001139	0.000455
N66	chr9	133927943	133927943	C	G		0.398550725	Missense_Mutation	NM_006059.3	LAMC3	p.Pro566Ala	MODERA	N	N	B	B	T	PASS	4.13E-05	0	0	0.000579	0	0	0	0	0.002273
N11	chr9	133928001	133928001	C	A		0.568421053	Missense_Mutation	NM_006059.3	LAMC3	p.Ser585Tyr	MODERA	D	D	P	P	D	PASS	8.35E-06	0	0	0.000116	0	0	0	0	0.001364
N43	chr9	133948110	133948110	G	A		0.546961326	Missense_Mutation	NM_006059.3	LAMC3	p.Arg1102His	MODERA	N	N	B	B	T	.	.	.	.	.	.	.	.	.	.
N51	chr9	133960947	133960947	C	T		0.477777778	Missense_Mutation	NM_006059.3	LAMC3	p.Ala1356Val	MODERA	N	D	D	P	D	PASS	1.66E-05	0	0	0	0	3.03E-05	0	0	.
N52	chr11	134079055	134079055	G	C		0.618421053	Missense_Mutation	NM_015261.2	NCAPD3	p.His260Asp	MODERA	N	N	B	B	T	PASS	0.00037129	0	0	0.005201	0	0	0	0	0.002273
N28	chr7	136700642	136700642	G	A	COSM484	0.244094488	Missense_Mutation	NM_000739.2	CHRM2	p.Val344Met	MODERA	D	N	B	B	T	PASS	2.48E-05	0	0	0	0	3.00E-05	0	0	.
N22	chr6	136913602	136913602	A	G		0.552419355	Missense_Mutation	NM_005923.3	MAP3K5	p.Val1010Ala	MODERA	N	N	B	B	T	PASS	0.0003546	0	0	0.004853	0	0	0	0	0.004091
N60	chr6	138199765	138199765	T	C		0.252459016	Missense_Mutation	NM_001270507.1	TNFAIP3	p.Phe395Leu	MODERA	D	N	P	B	D	.	.	.	.	.	.	.	.	.	0.000455
N51	chr9	139390631	139390631	C	T	COSM287	0.106666667	Nonsense_Mutation	NM_017617.4	NOTCH1	p.Trp2520*	HIGH	D	.	.	.	.	.	.	.	.	.	.	.	.	.	.
N60	chr9	139391424	139391424	G	A		0.458511548	Missense_Mutation	NM_017617.4	NOTCH1	p.Ala2256Val	MODERA	D	N	D	D	T	PASS	9.37E-06	0	9.36E-05	0	0	0	0	0.00134	.
N41	chr9	139391840	139391840	G	T		0.365814696	Missense_Mutation	NM_017617.4	NOTCH1	p.Asn2117Lys	MODERA	D	D	P	P	D	PASS	2.60E-05	0	0	0.000357	0	0	0	0	0.000909
N38	chr4	140270639	140270639	C	T		0.601941748	Missense_Mutation	NM_057175.3	NAA15	p.Arg239Cys	MODERA	D	D	D	D	D	PASS	9.78E-06	0	0	0	0	1.80E-05	0	0	.
N23	chr5	140432581	140432581	C	T		0.23255814	Missense_Mutation	NM_013340.3	PCDHB1	p.Ser509Leu	MODERA	D	D	P	P	D	.	.	.	.	.	.	.	.	.	.
N40	chr5	140432646	140432646	G	C		0.468965517	Missense_Mutation	NM_013340.3	PCDHB1	p.Val531Leu	MODERA	N	N	B	B	T	PASS	0.00012362	0	0	0.001733	0	0	0	0	0.003636
N64	chr7	140624440	140624440	C	T		0.551724138	Missense_Mutation	NM_004333.4	BRAF	p.Asp22Asn	MODERA	D	N	P	P	T	PASS	9.55E-05	0	0	0	0	0	0.000136	0	0.003636
N41	chr8	140743395	140743395	C	T		0.447257384	Missense_Mutation	NM_031466.7	TRAPPC9	p.Arg1217Glr	MODERA	N	N	B	B	T	PASS	0.00046813	0.00088	8.71E-05	0.004998	0	1.52E-05	6.07E-05	0.001147	0.003636
N43	chr5	140784066	140784066	C	T		0.405660377	Missense_Mutation	NM_018921.2	PCDHGA9	p.Ala516Val	MODERA	.	.	.	.	.	PASS	9.94E-05	0	0	0.001391	0	0	0	0	0.005
N55	chr5	140784679	140784679	C	A		0.153846154	Missense_Mutation	NM_018921.2	PCDHGA9	p.His720Gln	MODERA	.	.	.	.	.	PASS	2.48E-05	0	0	0	0	4.50E-05	0	0	.
N76	chr3	141206031	141206031	G	A		0.418918919	Missense_Mutation	NM_001303246.1	RASA2	p.Val36Met	MODERA	D	N	P	P	D	PASS	1.81E-05	0	0	0.000202	0	0	0	0	0.000455
N68	chr7	141719089	141719089	G	A		0.19047619	Missense_Mutation	NM_004668.2	MGAM	p.Val140Ile	MODERA	N	N	B	B	T	.	.	.	.	.	.	.	.	.	.
N11	chr4	147561276	147561276	CCAACCG	C		0.115384615	Indel	NM_004575.2	POU4F2	p.Gln183_Pro	MODERA	.	.	.	.	.	.	.	.	.	.	.	.	.	.	.
N22	chr4	147561899	147561899	G	A		0.515679443	Missense_Mutation	NM_004575.2	POU4F2	p.Arg390His	MODERA	D	D	D	D	D	.	.	.	.	.	.	.	.	.	.
N44	chr3	154146428	154146428	A	G		0.437037037	Missense_Mutation	NM_001038705.1	GPR149	p.Met326Thr	MODERA	D	N	P	B	D	PASS	6.79E-05	0	0	0.000233	0	0	0.000407	0	0.000909
N23	chr1	154408569	154408569	T	C	COSM220	0.70212766	Missense_Mutation	NM_000565.3	IL6R	p.Met311Thr	MODERA	.	.	.	.	.	PASS	5.15E-05	0	0	0.000707	0	0	0	0	0.002273
N52	chr4	156638421	156638421	T	A		0.637931034	Splice_Site	NM_000856.5	GUCY1A3	p.Asp561Glu	MODERA	D	N	B	B	T	.	.	.	.	.	.	.	.	.	.
N60	chr6	157469851	157469851	T	C		0.261744966	Missense_Mutation	NM_020732.3	ARID1B	p.Met882Thr	MODERA	D	D	P	B	D	.	.	.	.	.	.	.	.	.	.
N52	chr6	157525017	157525017	C	T	COSM385	0.405940594	Missense_Mutation	NM_020732.3	ARID1B	p.Arg1638Cys	MODERA	D	D	D	D	D	.	.	.	.	.	.	.	.	.	.

N40	chr6	157528016	157528016	G	A		0.515873016	Missense_Mutation	NM_020732.3	ARID1B	p.Arg1914Glr	MODERA	D	N	D	P	T	PASS	8.24E-06	9.63E-05	0	0	0	0	0	0	0.000455
N55	chr1	157665940	157665940	G	C		0.346938776	Missense_Mutation	NM_001320333.1	FCRL3	p.Ala341Gly	MODERA	.	.	.	.	.	.	.	.	.	.	.	.	.	.	.
N70	chr1	157666085	157666085	G	A	COSM244	0.578723404	Missense_Mutation	NM_001320333.1	FCRL3	p.Arg293Trp	MODERA	.	.	.	.	.	PASS	0.00024746	0.00029	0	0.00104	0.000151	0.00024	6.06E-05	0	0.003636
N60	chr1	158064154	158064154	G	A	COSM198	0.527210884	Missense_Mutation	NM_018240.6	KIRREL	p.Asp591Asn	MODERA	.	.	.	.	.	PASS	0.00022513	0	0	0.002776	0	3.53E-05	0	0	0.004545
N48	chr1	158064804	158064804	C	T	COSM402	0.321428571	Missense_Mutation	NM_018240.6	KIRREL	p.Ala723Val	MODERA	.	.	.	.	.	.	.	.	.	.	.	.	.	.	0.001818
N14	chr6	159642717	159642717	G	A	COSM120	0.457831325	Missense_Mutation	NM_032532.2	FNDC1	p.Arg252Gln	MODERA	N	N	D	D	T	PASS	1.66E-05	0	0	0	0.000151	1.50E-05	0	0	.
N28	chr6	159660816	159660816	C	T		0.472222222	Missense_Mutation	NM_032532.2	FNDC1	p.Thr1483Ile	MODERA	D	N	B	B	D	.	.	.	.	.	.	.	.	.	.
N51	chr1	161496342	161496342	G	C		0.363636364	Missense_Mutation	NM_002155.4	HSPA6	p.Asp632His	MODERA	.	.	.	.	.	PASS	1.94E-05	0	0	0.000241	0	0	0	0	0.000455
N31	chr4	165118825	165118825	G	C		0.216346154	Missense_Mutation	NM_012403.1	ANP32C	p.Asn13Lys	MODERA	.	.	.	.	.	.	.	.	.	.	.	.	.	.	.
N28	chr4	169292905	169292905	G	A		0.455696203	Missense_Mutation	NM_001012967.2	DDX60L	p.Arg1596Cys	MODERA	N	D	B	B	T	PASS	0.00024977	0	0	0.002896	0	6.68E-05	0	0	0.002273
N23	chr4	169315740	169315740	C	T		0.409638554	Missense_Mutation	NM_001012967.2	DDX60L	p.Arg1229Glr	MODERA	N	N	B	B	T	PASS	0.00036456	0.003574	0.000692	0	0	1.50E-05	0	0.008909	.
N76	chr6	170064322	170064322	T	C	COSM285	0.473317865	Missense_Mutation	NM_182552.4	WDR27	p.Arg282Gly	MODERA	N	D	B	B	T	PASS	0.00037945	0	0	0.005108	0	1.51E-05	0	0	0.007273
N75	chr1	172548378	172548378	C	T		0.209150327	Missense_Mutation	NM_016227.3	SUCO	p.Ser641Phe	MODERA	.	.	.	.	.	.	.	.	.	.	.	.	.	.	.
N76	chr5	176638846	176638846	A	G		0.529891304	Missense_Mutation	NM_022455.4	NSD1	p.Asn1149Ser	MODERA	N	N	B	B	T	PASS	4.95E-05	0	0	0.000463	0	3.00E-05	0	0	.
N60	chr5	176694670	176694670	A	G		0.523809524	Missense_Mutation	NM_022455.4	NSD1	p.Lys1752Glu	MODERA	D	D	D	D	D	.	.	.	.	.	.	.	.	.	.
N52	chr5	176722221	176722221	G	A		0.472222222	Missense_Mutation	NM_022455.4	NSD1	p.Val2618Ile	MODERA	N	N	B	B	T	PASS	0.00012366	0	8.64E-05	0.001272	0	1.50E-05	0.000121	0.001104	0.005
N64	chr4	177046390	177046390	G	A		0.274	Missense_Mutation	NM_170710.4	WDR17	p.Arg249His	MODERA	D	N	D	P	D	PASS	2.47E-05	0.000288	0	0	0	0	0	0	.
N11	chr4	177089798	177089798	G	T	COSM693	0.223214286	Splice_Site	NM_170710.4	WDR17		HIGH	N	.	.	.	.	.	.	.	.	.	.	.	.	.	.
N55	chr5	180030262	180030262	G	A	COSM490	0.153846154	Missense_Mutation	NM_182925.4	FLT4	p.Ser1341Leu	MODERA	N	N	B	B	T	PASS	8.50E-06	0	0	0	0	1.55E-05	0	0	0.000455
N70	chr2	186653723	186653723	A	C		0.455882353	Missense_Mutation	NM_173651.3	FSIP2	p.Gln620His	MODERA	N	D	.	.	T	.	.	.	.	.	.	.	.	.	0.000455
N48	chr2	186655266	186655266	A	C		0.393617021	Missense_Mutation	NM_173651.3	FSIP2	p.Ser1135Arg	MODERA	N	N	.	.	T	.	.	.	.	.	.	.	.	.	.
N69	chr2	186655924	186655924	C	T		0.380952381	Missense_Mutation	NM_173651.3	FSIP2	p.Ala1354Val	MODERA	N	N	.	.	T	PASS	7.10E-05	0	0	0	0	0	0.000131	0	.
N12	chr2	186665961	186665961	G	T		0.12195122	Missense_Mutation	NM_173651.3	FSIP2	p.Leu3976Phe	MODERA	N	D	.	.	D	.	.	.	.	.	.	.	.	.	.
N75	chr2	186666353	186666353	C	G		0.461538462	Missense_Mutation	NM_173651.3	FSIP2	p.Pro4107Arg	MODERA	N	D	.	.	D	.	.	.	.	.	.	.	.	.	.
N40	chr2	186666824	186666824	G	A		0.5125	Missense_Mutation	NM_173651.3	FSIP2	p.Ser4264Asn	MODERA	N	N	.	.	D	.	.	.	.	.	.	.	.	.	.
N69	chr2	186666859	186666859	G	A		0.282142857	Missense_Mutation	NM_173651.3	FSIP2	p.Asp4276As	MODERA	N	D	.	.	D	.	.	.	.	.	.	.	.	.	.
N23	chr2	186672712	186672712	A	T		0.162790698	Missense_Mutation	NM_173651.3	FSIP2	p.Ser6227Cys	MODERA	N	D	.	.	D	.	.	.	.	.	.	.	.	.	.
N70	chr2	186673101	186673101	G	T		0.221698113	Missense_Mutation	NM_173651.3	FSIP2	p.Leu6356Phe	MODERA	N	D	.	.	D	.	.	.	.	.	.	.	.	.	.
N70	chr2	186673119	186673119	G	T		0.231884058	Missense_Mutation	NM_173651.3	FSIP2	p.Leu6362Phe	MODERA	N	D	.	.	T	.	.	.	.	.	.	.	.	.	.
N46	chr1	200635460	200635460	G	C		0.491071429	Missense_Mutation	NM_001031725.5	DDX59	p.Leu137Val	MODERA	.	.	.	.	.	PASS	0.0001977	0	0	0.002773	0	0	0	0	0.004091
N48	chr2	201459998	201459998	G	A	COSM369	0.471428571	Missense_Mutation	NM_001159.3	AOX1	p.Arg36Gln	MODERA	N	N	B	B	T	PASS	8.48E-06	0	0	0.000116	0	0	0	0	.
N31	chr2	202134329	202134329	G	A	COSM335	0.522123894	Splice_Site	NM_001228.4	CASP8		HIGH	N	.	.	.	.	PASS	3.04E-05	0	0	0.000281	0	0	0	0	0.000455
N68	chr1	204236624	204236624	G	A		0.41025641	Missense_Mutation	NM_014935.4	PLEKHA6	p.Arg87Cys	MODERA	.	.	.	.	.	PASS	9.06E-05	0	0	0.001271	0	0	0	0	0.000909
N73	chr1	205901160	205901160	G	T		0.482728843	Missense_Mutation	NM_134325.2	SLC26A9	p.Thr127Asn	MODERA	.	.	.	.	.	PASS	6.64E-05	0	0	0.000925	0	0	0	0	0.003182
N22	chr1	212873042	212873042	G	C		0.212121212	Missense_Mutation	NM_018664.2	BATF3	p.Asn21Lys	MODERA	.	.	.	.	.	.	.	.	.	.	.	.	.	.	.
N60	chr1	237619984	237619984	G	A		0.61746988	Missense_Mutation	NM_001035.2	RYR2	p.Glu521Lys	MODERA	.	.	.	.	.	PASS	9.12E-05	0	0	0.001277	0	0	0	0	0.002273
N64	chr1	237780587	237780587	T	C		0.363636364	Splice_Site	NM_001035.2	RYR2	p.Met1906Thr	MODERA	.	.	.	.	.	PASS	3.95E-05	0	0	0.000587	0	0	0	0	.
N68	chr1	237801763	237801763	A	G		0.188679245	Missense_Mutation	NM_001035.2	RYR2	p.Asp2300Gly	MODERA	.	.	.	.	.	.	.	.	.	.	.	.	.	.	.
N55	chr1	241261966	241261966	C	G		0.272727273	Splice_Site	NM_002924.5	RGS7	p.Gly59Arg	MODERA	.	.	.	.	.	.	.	.	.	.	.	.	.	.	.
N41	chr1	241946599	241946599	G	A	COSM134	0.187878788	Missense_Mutation	NM_144625.4	WDR64	p.Arg864His	MODERA	.	.	.	.	.	PASS	0.00041288	0.000674	0.000519	0.003824	0	4.51E-05	6.06E-05	0.006637	0.003182
N51	chr1	241946599	241946599	G	A	COSM134	0.555555556	Missense_Mutation	NM_144625.4	WDR64	p.Arg864His	MODERA	.	.	.	.	.	PASS	0.00041288	0.000674	0.000519	0.003824	0	4.51E-05	6.06E-05	0.006637	0.003182
