# Supplementary material for: Characteristic gene alterations in primary gastrointestinal T- and NK-cell lymphomas
Source: Leukemia. 2019 Jan 23;33(7):1797–832. doi: 10.1038/s41375-018-0309-4 (PMC6755973; doi:10.1038/s41375-018-0309-4)
Supplement: Supplementary file 22 — Supplementary table 8 [file 41375_2018_309_MOESM22_ESM.pdf]

Supplementary Table 8. Univariate and multivariate cox analysis for overall survival of systemic mature T and NK cell lymphomas

Variables	Category	Univariate analysis				Multivariate analysis			
		P value	HR	95.0% CI		P value	HR	95.0% CI	
				Lower	Upper			Lower	Upper
Sex	Male vs Female	0.905	1.03	0.66	1.6				
Age	≥60 yrs vs <60yrs	**0.002**	1.95	1.27	3	0.007	2.241	1.246	4.031
Anatomical location	GI vs non-GI	**0.009**	2.04	1.2	3.49	0.565	0.541	0.067	4.393
Subtypes	AITL vs ITCL-NOS	0.902	0.925	0.268	3.191	0.24	0.184	0.011	3.102
	ALCL, ALK- vs ITCL-NOS	0.572	1.431	0.412	4.962	0.653	0.524	0.031	8.757
	ALCL, ALK+ vs ITCL-NOS	**0.019**	0.116	0.019	0.701	0.054	0.038	0.001	1.052
	EATL vs ITCL-NOS	0.791	1.214	0.29	5.091	0.805	1.263	0.198	8.068
	ENKTL vs ITCL-NOS	0.093	0.348	0.102	1.191	0.144	0.155	0.013	1.893
	PTCL-NOS vs ITCL-NOS	0.3	0.532	0.162	1.753	0.221	0.176	0.011	2.845
EBV in tumor	Positive vs negative	0.765	0.93	0.57	1.51				
Ann-Arbor stage	III-IV vs I-II	**0.007**	2.13	1.23	3.69	0.134	1.858	0.826	4.182
**KCNB2 protein**	**Low vs High**	**0.031**	**1.66**	**1.05**	**2.62**	***0.078***	**1.638**	**0.946**	**2.835**

AITL, angioimmunoblastic T cell lymphoma; ALCL, anaplastic large cell lymphoma; ALK-, ALK-negative; ALK+, ALK-positive;
MEITL, monomorphic epitheliotropic intestinal T-cell lymphoma; ENKTL, extranodal NK/T-cell lymphoma of nasal type;
PTCL-NOS, peripheral T cell lymphoma, not otherwise specified; ITCL-NOS, intestinal T cell lymphoma, not otherwise specified;
HR, hazard ratio; CI, confidence interval
