# Supplementary material for: Characteristic gene alterations in primary gastrointestinal T- and NK-cell lymphomas
Source: Leukemia. 2019 Jan 23;33(7):1797–832. doi: 10.1038/s41375-018-0309-4 (PMC6755973; doi:10.1038/s41375-018-0309-4)
Supplement: Supplementary file 23 — Supplementary table 9 [file 41375_2018_309_MOESM23_ESM.pdf]

Supplementary Table 9. Primer sets used for Sanger sequencing

GENE	REF	ALT	SAMPLE	X20	FASTA	PRIMER
KCNB2 -1	C	A	G05	CTTAGAAGAAACCCCCTCCCAG	GCCTCTCACTTGCAGATGAAGTTCCCAACCGACCTCCCAGGGACAGAAGAG<br>CACCAAAGAGCTAGGGGCC<br>CCCCGTTTCTAACTCTATCCAGAGAGAAAGGACCTGCTGCCAGGGATGGCA	LEFT PRIMER ; CGCAGCACATCAGTACCATC 59.07<br>RIGHT PRIMER ; AAGTCTTCGTCGTCACCAGT 58.97 (199bp)
KCNB2 -1	C	A	G18			
KCNB2 -2	C	T	G14	TCATGCGCATCCTCAGGATCCTGAAACTC	AGGATTTGCTGACTTGGACTTTTCTCTTTTCCAGATCCTGGCCATCGTGTCTA<br>TCCTGTTCATTGTGCTT<br>TCCACCATTGCTTTGTCTCTCAATACGCTGCCGGAGCTGCAGGAAACGGACG	LEFT PRIMER ; TGATTTGCTGGCCATCTTGC 59.47<br>RIGHT PRIMER ; GTGAAACCCAGAGACTGCAG 58.48 (174bp)
KCNB2 -2	G	T	G16			
JAK3 -1	A	G	G03	GCTGACCCC<br>GGGGTCAGC	GAAACAGACCAGAGAGGGTGGGTCACTTGCCCAAGGTCACACAGCAAGTTA<br>AAGGTACAAGCTGGGCTCT<br>GTGAGGCCTCCGCAGAATCTGTCCCTCGCCCCCACCATAATGTCACTCCTAC	LEFT PRIMER ; GCTATCCTTGACCTGCCAGT 59.46<br>RIGHT PRIMER ; GTTGTTAACCTGCAGACCCC 58.76 (197bp)
JAK3 -1	A	G	G13			
JAK3 -2	C	T	G04	CTCCCGAGC<br>GCTCGGGAG	GGCAGGAGACCAGGGTGCAAGTGTGGAAGGGAAGTTCATTGGAAGCTTGAG<br>CAAGGGCCTTGAATGCCAG	LEFT PRIMER ; CAATGTCTCTGCCCGGAAG 58.23<br>RIGHT PRIMER ; GGTGAGCACTGAGGGAATGA 59.38 (197bp)
JAK3 -3	G	A	G09	GCTCGCTGC<br>GCAGCGAGC	GGAGTTCGAGACCAGCCTGACCAACATGGAGAAACCCCGTCTCTACTAAAA<br>ATACAAAATTAGCCGGGCG<br>TGGTGGTGCATGCCTGTAATCCCAGCTACTCGGGAGGCTGAGGCAGGAGAA	LEFT PRIMER ; ACCTTCCCCAGTCATTCCTG 59.00<br>RIGHT PRIMER ; GGTGGGAAGAACAGCCTAGA 58.72 (16bp)
JAK3 -3	G	A	G16			
JAK1 -1	G	A	G16	GAAGGCAGA	CTCTGACTGTTATCTCAGTGAGTGCTTATGCCTGTGAGGTAGGTACACCATT<br>GTTACCATCTCCATTCACTGCTGAGGATCTTGAGGCATAAGAGGTCAAGTAA	LEFT PRIMER ; AGCCTCAGACTGATGGATGT 58.12<br>RIGHT PRIMER ; ATTACCCAGGACAGAGTGCC 59.09 (217bp)
JAK1 -2	C	T	G03	CTCACGGGC	CACGAAGCCCAATGAGATACGTGCCACTATTTAGCCACATTCAGTGATGAG<br>GGAGCTGGAGCCAAGGGGCACCTGTGATGTGCCTAAGTCACACAGATGATT	LEFT PRIMER ; TCACACAGATGATTGGCAGC 58.54<br>RIGHT PRIMER ; CTGAGCTTGATGAATGGGCC 58.97 (240bp)
JAK1 -3	C	A	G10	GTAGCTCAGTTGTTTGGCA	TGTACTCTGAGGCCGAGTAGTGTCCACTGAAGTGGACTGTCGTAAGGGGAT<br>GAAGGAGAGGACCGTGCCACAGACCAGGTTCCAGACATGGCTATTTTTATG<br>CCTACAGATATCATGGTGGAAGAGTTTGTGGAAGGGGGTCCTCTGGATCTCT	LEFT PRIMER ; CACAGACCAGGTTCCAGACA 59.24<br>RIGHT PRIMER ; ACTCTGGTTTCTGGTGGGAC 59.23 (215bp)
	T	G	G16			
JAK1 -4	C	A	G16	GAAACTCAG	CTTGTGCAGATATATATTATTCAGTGTGTAGATTATTTCAAGCATGAAATTC<br>GTGTCTTCTCTGGAAAATCCCGACTTGAGTACTGTTACAAAGTCAAGACGAT	LEFT PRIMER ; TGCATGTCTAGTGAGCAGTGA 59.10<br>RIGHT PRIMER ; CCAAGGAAGAGAAAGCCAGC 58.83 (205bp)
